# Dual role of benzophenone enables a fast and scalable C-4 selective alkylation of pyridines in flow†

**DOI:** 10.1039/d2sc04990b

**Published:** 2022-10-10

**Authors:** Jesús Sanjosé-Orduna, Rodrigo C. Silva, Fabian Raymenants, Bente Reus, Jannik Thaens, Kleber T. de Oliveira, Timothy Noël

**Affiliations:** Flow Chemistry Group, Van’t Hoff Institute for Molecular Sciences (HIMS), University of Amsterdam Science Park 904 1098 XH Amsterdam The Netherlands t.noel@uva.nl https://www.noelresearchgroup.com/; Departamento de Química, Universidade Federal de São Carlos SP 13565-905 Brazil

## Abstract

The efficient C-4 selective modification of pyridines is a major challenge for the synthetic community. Current strategies are plagued with at least one drawback regarding functional group-tolerant electronic activation of the heteroarene, mild generation of the required alkyl radicals, regioselectivity, safety and/or scalability. Herein, we describe a fast, safe and scalable flow process which allows preparation of said C-4 alkylated pyridines. The process involves a photochemical hydrogen atom transfer (HAT) event to generate the carbon-centered radicals needed to alkylate the C-2 blocked pyridine. In a two-step streamlined flow process, this light-mediated alkylation step is combined with a nearly instantaneous inline removal of the blocking group. Notably, cheap benzophenone plays a dual role in the pyridine alkylation mechanism by activating the hydrocarbon feedstock reagents *via* a HAT mechanism, and by acting as a benign, terminal oxidant. The key role of benzophenone in the operative reaction mechanism has also been revealed through a combination of experimental and computational studies.

## Abstract 2

A synergistic combination of a photochemical strategy with microfluidic techniques allowed a fast Minisci-type reaction, which could also be scaled up to gram scale. Mechanistic studies provided insight into the key role of benzophenone.

## Graphical abstract

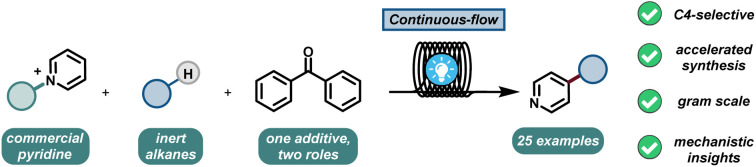

## Introduction

Pyridines are privileged scaffolds and their incorporation into biologically-active molecules can dramatically improve their potency.^1^ Hence, synthetic tools that can selectively edit this moiety are highly desired to enable the streamlined synthesis of various pharmaceuticals and agrochemicals.^2^

**Fig. 1:**
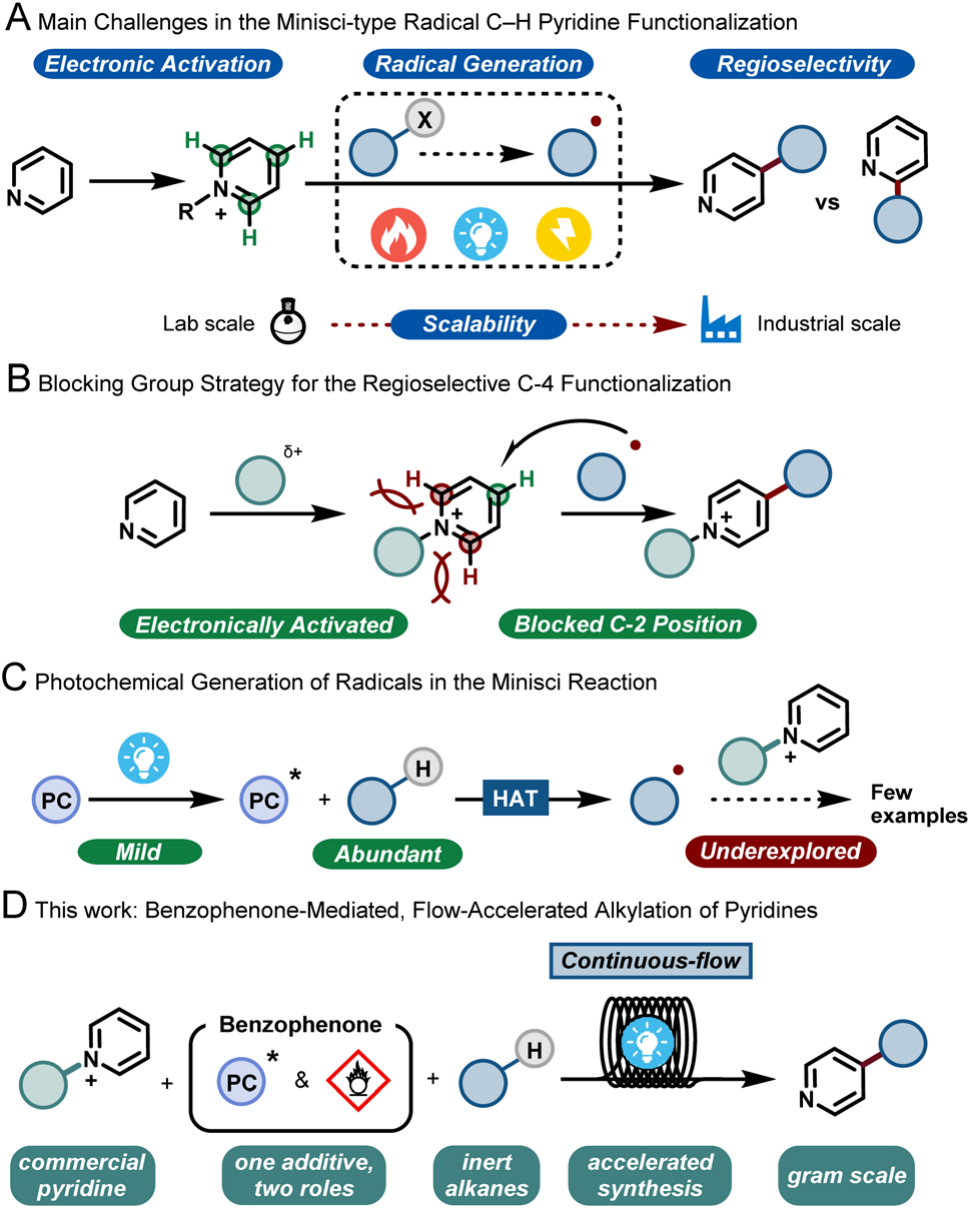
C-4 selective alkylation of pyridines. (A) Main challenges in the Minisci-type pyridine functionalization reactions. (B) Blocking group strategy for the C-4 regioselective functionalization. (C) Photochemical generation of alkyl radicals in the Minisci reaction. (D) A benzophenone-mediated, flow-accelerated alkylation of blocked pyridines (this work).

In the 1970s, Minisci and co-workers reported upon the thermal generation of alkyl radicals *via* decarboxylation of carboxylic acids using silver salts. These nucleophilic radicals were subsequently exploited to alkylate various heteroarenes, including pyridines, under strongly acidic and oxidative conditions.^3^ More than 50 years later, this strategy, coined as the Minisci reaction, still stands and has evolved into a key method for the modification of heteroarenes.^4^ However, between academic discovery and practical use in the pharmaceutical and agrochemical industry, there remain many roadblocks for the widespread implementation of the Minisci reaction. These challenges include (i) a functional-group tolerant electrophilic activation of the basic heteroarene, (ii) a reliable and mild generation of the alkyl radicals; (iii) the poor regioselectivity of the radical addition to the aromatic ring^5^ leading to various byproducts and (iv) the safe and challenging scale up to relevant quantities for process chemists (Fig. 1A). In recent years, the use of bulky, C-2 blocking groups has proven to be very effective to enable both regioselectivity towards the C-4 position and efficient electrophilic activation of the pyridine moiety (Fig. 1B).^6^

With respect to the generation of radicals, since the initial thermal decarboxylation methodology developed by Minisci, several milder strategies have been discovered. As a prime example, photocatalysis has emerged in the past decade as a powerful tool for the efficient formation of open-shell reactive intermediates.^7^ Among the different radical precursors available, the direct activation of ubiquitous C–H bonds is perhaps one of the most desirable synthetic targets, as it bypasses the need for lengthy pre-functionalization steps. The homolytic cleavage of such inert C–H bonds *via* hydrogen atom transfer (HAT) has recently been shown to be a very powerful approach to enable both early and late stage functionalization of hydroalkanes.^8^

However, the coupling of the HAT-generated radical with activated pyridines is so far an underdeveloped strategy (Fig. 1C). Few examples are reported in the literature, usually requiring excess of hydrocarbons, inert conditions, transition metals and/or very extended reaction times, making such strategies less practical. Consequently, in order to make the transition from academic discovery to a scalable process, synthetic methodologies are urgently needed that use cheap and readily-available starting materials, involve few and non-toxic additives, require short reaction times and are scalable.

Given the experience of our group in developing multidisciplinary synthetic approaches,^9^ we wondered if the aforementioned issues could be simultaneously solved by developing a continuous-flow synthetic strategy. Flow technology has been shown to uniquely pair with photochemistry, by effectively reducing the reaction times, by improving reaction selectivity due to excellent mass and photon transfer characteristics, and by enabling facile scale up.^10^ Moreover, using such a flow strategy would also enable us to combine both the photochemical C-4 functionalization of the pyridine and the subsequent removal of the C-2 blocking group, ultimately delivering an operationally simple process that should ensure the preparation of large quantities of C-4 alkylated pyridines using hydrocarbons as cheap and abundantly available coupling partners (Fig. 1D).

## Discussion

With this blueprint in mind, we commenced our investigations by testing a model system consisting of a commercially available C2-blocked pyridine 1,^6*h*^ cyclohexane 2a, tetrabutylammonium decatungstate (TBADT) as the HAT photocatalyst^11^ and stoichiometric amounts of a terminal oxidant, *i.e.* (NH_4_)_2_S_2_O_8_. We subjected the initial reaction mixture under air in a continuous-flow microreactor (ID = 0.8 mm, 3.3 mL) and exposed it to UV-A light irradiation (*λ* = 365 nm, 60 W input power) (See ESI† for details). Under these conditions, we could observe the regioselective C-4 alkylation of 1 albeit with a low yield for 3a (Table 1, entry 1). In addition, precipitation of the photocatalyst and the oxidant in the reaction coil was observed, which would lead to clogging problems over time.^12^ We wondered if the highly-charged nature of TBADT could be an issue under these conditions, so we decided to switch to a simpler organic HAT mediator, such as benzophenone (BP1).^13,14^ Although similar low yields were obtained (Table 1, entry 2), a completely homogeneous solution was observed during the entire experiment.

**Table 1:** Optimization of reaction conditions for the C–C bond-forming step

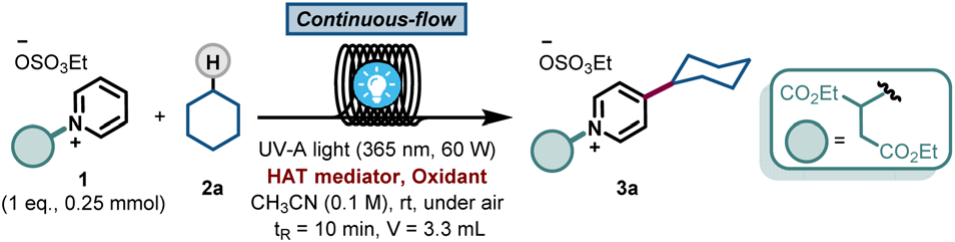				
Entry	2a (eq.)	HAT mediator	Oxidant	3a^a^ [%]
1	10	TBADT (4 mol%)	(NH_4_)_2_S_2_O_8_ (2 eq.)	24^b^
2	10	BP1 (20 mol%)	(NH_4_)_2_S_2_O_8_ (2 eq.)	28^b^
3	20	BP1 (20 mol%)	Oxygen	0
4	10	BP1 (20 mol%)	None	13
5	10	BP1 (1 eq.)		63
6	10	BP2 (1 eq.)		36
7	10	BP3 (1 eq.)		52
8	10	BP4 (1 eq.)		55
9	2.5	BP1 (1.5 eq.)		70
10	2.5	BP1 (1.5 eq.)		27^c^
11	2.5	None		0
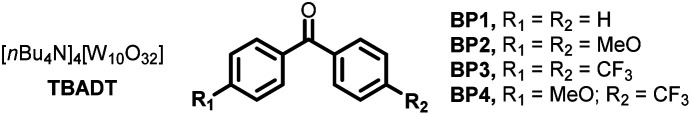				

a Yields determined by ^1^H NMR spectroscopy using 1,3,5-trimethoxybenzene as external standard.

b Ammonium persulfate required a CH_3_CN : H_2_O (1 : 1) mixture as solvent for solubility issues.

c In batch (See ESI for further details).

After selecting BP1 as the HAT mediator, different oxidants and solvents were screened (see ESI† for details). Unexpectedly, when using oxygen as oxidant, the reaction was completely suppressed (Table 1, entry 3). Notably, a blank experiment revealed another interesting observation: without the addition of any external oxidant, we observed the formation of 3a in 13% yield using 20 mol% of BP1 (Table 1, entry 4). This finding, in combination with entry 3, prompted us to question if benzophenone could also act as a terminal oxidant. When repeating the reaction with a stoichiometric amount of BP1, the product 3a was obtained in 63% yield, demonstrating the ability of BP1 to not only engage in the C(sp^3^)–H activation event, but also to serve as terminal oxidant (Table 1, entry 5). Next, we evaluated a diverse set of substituted benzophenones. Electron-rich (BP2, Table 1, entry 6), electron-poor (BP3, Table 1, entry 7) and a push–pull system (BP4, Table 1, entry 8) were subjected to the reaction conditions, but none of them outperformed the non-substituted and commercially available BP1 as mediator. Finally, increasing the amount of BP1 to 1.5 equivalents allowed us to decrease the equivalents of the hydroalkane 2a to 2.5 equivalents, delivering the target compound 3a in 70% yield (Table 1, entry 9).

This result was obtained without any special precautions during the reaction preparation, including the use of wet reagents/solvents and working under air, making our reaction protocol particularly user-friendly. Interestingly, when repeating the reaction under conventional batch conditions, the yield for the benzophenone-mediated alkyl–pyridyl coupling dropped to 27% (Table 1, entry 10), highlighting the importance of an efficient irradiation of the reaction mixture under microfluidic conditions. No reactivity was observed when the reaction was performed without BP1 (Table 1, entry 11). Having found proper reaction conditions for the alkyl–pyridyl bond-forming reaction, we next aimed to remove the C2-blocking group attached to the pyridine moiety. To do so, we merged the reaction stream exiting the photochemical flow reactor with a dichloromethane solution containing the organic base 1,8-diazabicyclo[5.4.0]undec-7-ene (DBU). In only 20 min overall residence time, we could perform both the photomediated alkylation and the removal of the blocking group without the need for intermediate isolation. In addition, this operationally simple protocol also allowed us to scale up the process without re-optimization of the reaction conditions, which often plagues conventional batch scale up procedures. Simply by continuously pumping starting materials into the reactor assembly, we could scale up the reaction to a gram-scale, with even slightly higher reaction yields (74% isolated yield for 4a, 91% purity, 210 mg hour^−1^).

**Fig. 2:**
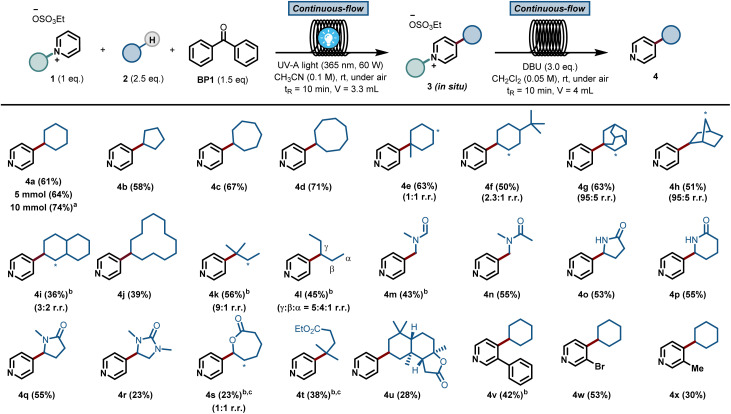
Substrate scope of the benzophenone-mediated alkylation of pyridines with subsequent deprotection step. Reaction conditions: 0.5 mmol of 1, 1.25 mmol. of 2, 0.75 mmol. of BP1 in CH_3_CN (3 mL) and using 1.5 mmol of DBU in 5 mL of CH_2_Cl_2_ for the deprotection step. Isolated yield in brackets. * Indicates the position of the minor regioisomer formed; (a) 10 mmol scale, (b) 10 equiv. of 2; (c) 1 h of residence time in the first step.

The scope of pyridines that can be C-4 alkylated using our protocol is illustrated in Fig. 2. First, a diverse set of hydrocarbons bearing strong, non-activated C(sp^3^)–H bonds (4a–4l) were subjected to the photochemical reaction conditions. Benzophenone was able to cleave the C(sp^3^)–H bond in various cyclic alkane feedstocks, such as cyclopentane (4b), cycloheptane (4c), cyclooctane (4d), adamantane (4g), norbornane (4h), decalin (4i) and cyclododecane (4j) and the ensuing nucleophilic radical was subsequently coupled with the C2-blocked pyridine.

Also, linear alkanes, such as isopentane (4k) and *n*-pentane (4l), were selectively activated by benzophenone at their most hydridic C(sp^3^)–H bonds. Various amides, including dimethylformamide (DMF, 4m) and dimethylacetamide (DMA, 4n), could be activated α-to-N C(sp^3^)–H. Furthermore, 5 and 6-membered lactams (4o–4r), lactones (4s) and esters (4t) were compatible with our HAT-induced Minisci protocol. Notably, the sesquiterpene lactone natural product sclareolide (4u) could be regioselectively pyridinylated, demonstrating the potential of this protocol to act as a late-stage functionalization method. Finally, various C-3 substituted pyridines (4v–4x) were also compatible with the reaction conditions, giving rise to the corresponding C-4 alkylated compounds in good isolated yields. Unfortunately, alkanes bearing cyclic amines, cyclic ethers, aromatic rings or halogens gave rise to either low yields or complex reaction mixtures (see ESI† for details).

**Fig. 3:**
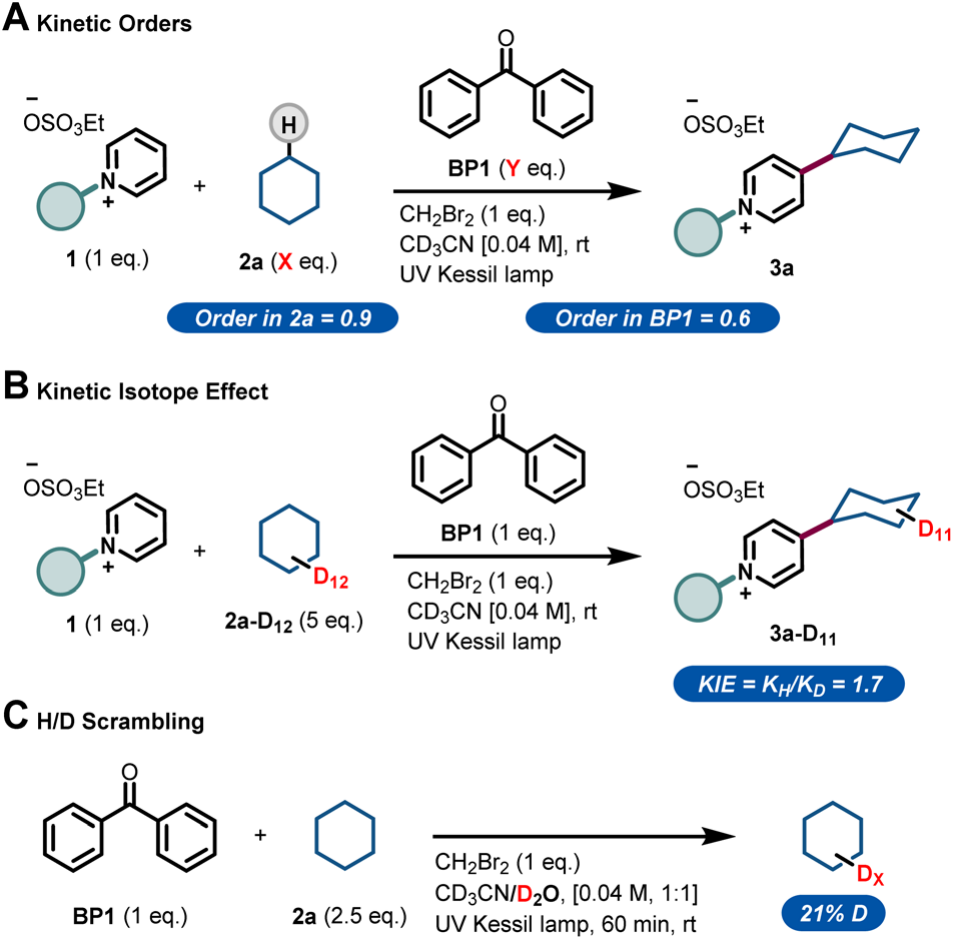
Experimental mechanistic investigations. (A) Kinetic orders. (B) Kinetic Isotope Effect (KIE). (C) H/D scrambling.

Next, we sought to investigate the mechanism of this process and, in particular, to elucidate the role of benzophenone. Using the initial rates method we could extract the experimental reaction rates at different concentrations (Fig. 3A).^15^ From these kinetic investigations, the reaction order of cyclohexane was determined to be 0.9, suggesting a first-order dependence regarding the hydrocarbon concentration under these conditions.

Interestingly, the kinetic order of BP1 was measured to be 0.6. This fractional order suggests that the role of benzophenone is more complex, and implies that BP1 might be involved in different elementary steps through the entire mechanistic scenario. Similarly, to get further insights about the initial C(sp^3^)–H bond cleavage of cyclohexane, we examined the kinetic isotope effect (KIE) (Fig. 3B). To do that, we compared the initial rates of both a standard reaction and a reaction using deuterated cyclohexane (2a-d_12_). A KIE of 1.7 was determined, which suggests that the C(sp^3^)–H bond cleavage of the alkane might be involved in the rate-determining step.^16^ Further H/D scrambling experiments demonstrated the reversibility of the process under the described reaction conditions, as we observed partial deuteration of cyclohexane when repeating the reaction using D_2_O as cosolvent (Fig. 3C).

**Fig. 4:**
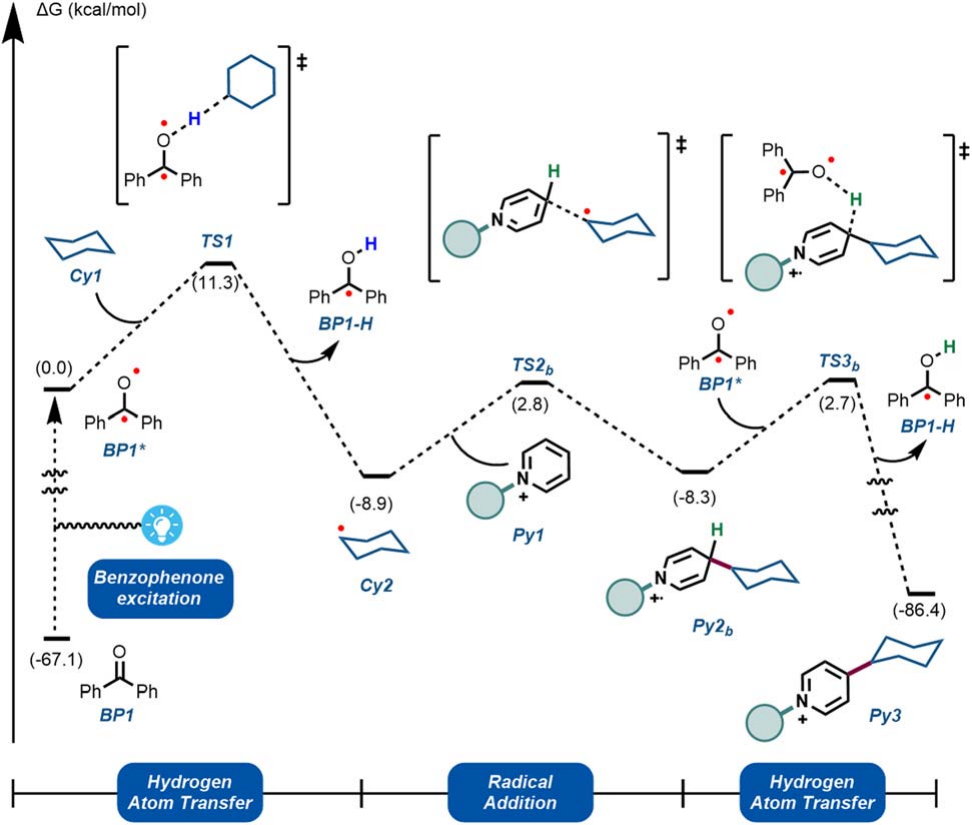
Computational mechanistic investigations. Calculations were performed at the ωB97X-D/6-31+G(d,p)/CPCM (acetonitrile) level of theory. See ESI† for computational details.

To further understand the experimentally-obtained mechanistic insights, we decided to simulate a plausible scenario that could explain this dual role of the benzophenone using density functional theory (DFT) calculations. The obtained qualitative reaction profile with the corresponding energies is depicted in Fig. 4 (See ESI† for further computational details). The reaction kicks off with UVA-light photoexcitation of the ground state benzophenone BP1 to its triplet state BP1*. This highly electrophilic species, regarded as the 0.0 in the energy profile, is responsible for the homolytic cleavage of a C(sp^3^)–H bond in the alkyl partner Cy1, through TS1 at 11.3 kcal mol^−1^. This low energetic barrier is in agreement with the absence of a primary KIE (Fig. 3B). The ensuing carbon-centered radical Cy2 resides in an endergonic position (−8.9 kcal mol^−1^) relative to BP1*. The reversibility of this step was observed experimentally (Fig. 3C), demonstrating that the reverse energetic barrier (20.2 kcal mol^−1^) can be overcome at room temperature under the described reaction conditions.

Subsequently, species Cy2 can engage in a radical addition at the only electronically and sterically accessible position of Py1, through TS2b. Given that the reversibility of TS1 was demonstrated experimentally under these conditions, we can assume that the energetic barrier for TS2b (11.7 kcal mol^−1^) is also within reach. Finally, we simulated the re-aromatization of the reduced Py2b by a second molecule of BP1*via a second hydrogen atom transfer event in TS3b. The energetic barrier for this last step from Cy2 (11.0 kcal mol^−1^) is comparable to TS2b and to TS1, showcasing that all the elementary steps are feasible at room temperature. This demonstrates that BP1* can participate not only in the first HAT reaction, but also in the terminal oxidation of Py2b, thus explaining the fractional kinetic order observed experimentally (Fig. 3A).

**Fig. 5:**
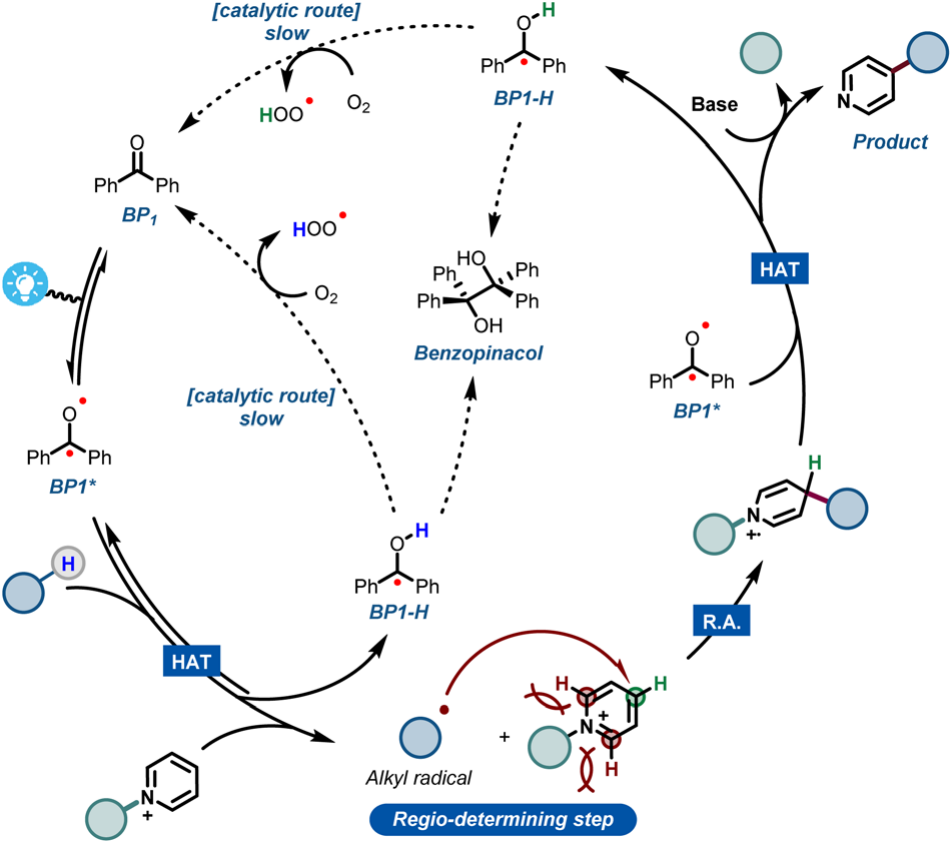
Plausible mechanistic hypothesis for the benzophenone-mediated photoalkylation of pyridines.

Combining all experimental and computational evidence, we can portray a plausible reaction mechanism for the benzophenone-enabled photomediated C4-alkylation of pyridines (Fig. 5). A photoexcited ketone in the triplet state BP1* is responsible for the cleavage of the strong C(sp^3^)–H bond *via* HAT, giving rise to a nucleophilic alkyl radical and concomitant formation of BP1-H. It should be noted that this protonated species can be re-oxidized to the original benzophenone under an oxygen atmosphere, leading to a catalytic pathway.^14*f*^ However, this reoxidation pathway is particularly slow as shown by our experiments, making it less practical for scale up (see ESI†). The generated alkyl radical is subsequently added to the activated pyridine, establishing the targeted alkyl–pyridyl bond. Finally, the ensuing radical cation has to be rearomatized. Notably, this is the specific step that often requires harsh, external oxidants in many Minisci reaction protocols. However, under our set of reaction conditions, this step can be carried out mildly using a second molecule of BP1*.

It is worth noting that oxygen is also capable of engaging into this oxidation step, but again requiring quite long reaction times. The second molecule of reduced BP1-H can be re-oxidized in the same fashion by oxygen, closing what happens to be a rather slow, catalytic cycle.

Additionally, during our optimizations with BP1, we observed the formation of small quantities of the dimerized benzopinacol derivative. This evidence also corroborates with the proposed radical BP1-H and with the need for more than 1 eq. of BP1, thus suggesting a competition between the re-oxidation of BP1-H by O_2_ and its radical recombination (Fig. 5). Finally, the removal of the blocking group using a non-nucleophilic organic base gives rise to the desired C-4 alkylated pyridine.

## Conclusions

In conclusion, we have developed an efficient, safe and scalable two-step flow protocol to enable the C-4 selective alkylation of pyridines. The uninterrupted process involves a benzophenone-induced HAT generation of radicals, a subsequent alkylation of a C-2 blocked pyridine and the ultimate removal of the blocking group, requiring only 20 min of reaction time in total. The scope of this transformation is broad, and the targeted pyridines can be alkylated with a variety of activated and non-activated hydrocarbon feedstocks. Finally, a combined experimental and computational mechanistic investigation was carried out to elucidate the reaction mechanism and to highlight the role of benzophenone as HAT reagent and mild oxidant.

## Data availability

Experimental details, used materials, sample preparation and analytical data (NMR) for the compounds 4a–4x. All the raw computational data is available on the ioChem-BD^17^ repository and can be freely accessed at https://doi.org/10.19061/iochem-bd-6-156.

## Author contributions

J. S.-O. conceived the idea for this work. J. S.-O. and J. T. carried out initial optimization studies. R. C. S. developed the refined reaction conditions and the substrate scope, with the help of B. R. and J. S.-O. carried out the experimental and computational mechanistic studies. F. R. and B. R. studied the effect of oxygen in the reaction. T. N. provided direction for the scientific strategy. J. S.-O. and T.N. wrote the manuscript with input from all the authors.

## Conflicts of interest

There are no conflicts to declare.

## Supplementary Material

SC-013-D2SC04990B-s001
