# Supplementary material for: Dual role of benzophenone enables a fast and scalable C-4 selective alkylation of pyridines in flow
Source: Chem Sci. 2022 Oct 10;13(42):12527–32. doi: 10.1039/d2sc04990b (PMC9629060; doi:10.1039/d2sc04990b)
Supplement: SC-013-D2SC04990B-s001 [file SC-013-D2SC04990B-s001.pdf]

Electronic Supplementary Material (ESI) for Chemical Science.
This journal is © The Royal Society of Chemistry 2022

SUPPORTING INFORMATION

Dual Role of Benzophenone Enables a Fast and Scalable C-4 Selective Alkylation of Pyridines in Flow

Jesús Sanjosé-Orduna+, Rodrigo C. Silva+, Fabian Raymenants, Bente Reus, Jannik Thaens, Kleber T. de Oliveira and Timothy Noël*

Table of Contents

1. GENERAL INFORMATION 3
2. SETUP OF THE REACTION IN FLOW 4
3. REACTION OPTIMIZATION 6
3.1. Photocatalyst and oxidant screening 7
3.2. Equivalents of Benzophenone 8
3.3. Benzophenones screening 9
3.4. Solvent screening 10
3.5. Residence time optimization 11
3.6. Concentration optimization 12
3.7. H-Donor and photocatalyst equivalent correlation 13
3.8. Optimization of the residence time for the deprotection step in the telescoped setup 14
4 SCOPE LIMITATIONS 15
5 SYNTHETIC PROCEDURES 15
5.1 Synthesis of pyridinium carboxylate salt: 15
5.2 Synthesis of substituted pyridinium salts (1a-1d): 15
5.3 General procedure A: Photoflow and Fed-batch deprotection 16
5.4 General Procedure B: Telescope setup: 16
6 EXPERIMENTAL DATA 18
7 EXPERIMENTAL MECHANISTIC STUDIES 27
7.2 ON-OFF experiment 28
7.3 Kinetic Orders 28
7.4 Kinetic Isotope Effect 31
7.5 H/D scrambling 32
7.6 Oxygen Effect in Flow 33
7.7 Oxygen Effect in Batch 35
8 COMPUTATIONAL MECHANISTIC STUDIES 36
8.1 Overall Free Energy Profiles 37
8.2 Cartesian Coordinates and Absolute Energies 38
9 NMR DATA 58
10 REFERENCES 82

1. GENERAL INFORMATION

Reagents and solvents were bought from Sigma Aldrich, TCI and fluorochem and were used as received. Technical solvents were bought from VWR International and were used as received. All capillary tubing and microfluidic fittings were purchased from IDEX Health & Science. Syringe pumps were purchased from Chemix Inc. model Fusion 200 Touch. The crude products were purified by flash column chromatography on silica gel (P60, SILICYCLE). TLC analysis was performed using silica on aluminum foils TLC plates (F254, Supleco Sigma Aldrich) with visualization under ultraviolet light (254 nm). Organic solutions were concentrated under reduced pressure on a Büchi rotary evaporator (in vacuo at 40 ºC, ~20 mbar). The regioisomeric and diastereomeric ratios were determined by ^{1}H NMR analysis of the crude reaction mixture through the integration of diagnostic signals. ^{1}H (400 MHz), ^{13}C (100 MHz) spectra were recorded on ambient temperature using a Bruker-Avance 400. ^{1}H NMR spectra were reported in parts per million (ppm) downfield relative to $CDCl_3$ (7.26 ppm) and ^{13}C NMR spectra were reported in ppm relative to $CDCl_3$ (77.00 ppm). NMR spectra used the following abbreviations to describe the multiplicity: s (singlet), d (doublet), t (triplet), q (quartet), p (pentet), h (hextet), hept (heptet), m (multiplet), dd (double doublet), td (triple doublet). Coupling constants (J) were reported in hertz (Hz). NMR data were processed using the MestReNova 14 software package. Known products were characterized by comparing to the corresponding ^{1}H NMR and ^{13}C NMR from the literature. High-resolution mass spectra (HRMS) were recorded by using an AccuTOF GC v 4g, JMS-T100GCV Mass spectrometer (JEOL, Japan). The names of all products were generated using the PerkinElmer ChemBioDraw Ultra v. 18.0.0 software package.

2. SETUP OF THE REACTION IN FLOW

For the optimization experiments and the evaluation of the scope, a Vapourtec device with a UV-150 photochemical reactor was used, equipped with 60 W, 365 nm LED. The active reactor volume was 3.33 mL using a coil of PFA (ID: 0.8 mm).

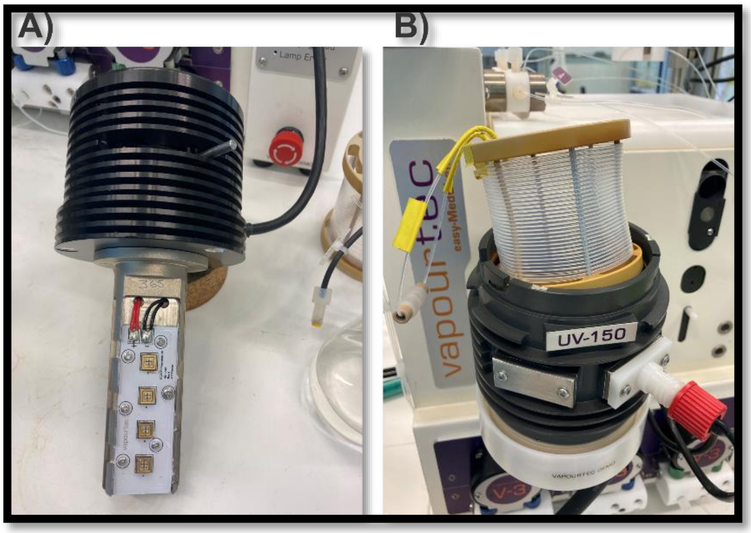

Figure S1: Photoreactor assembling. **A)** Light source using 365 nm at 60 W, and **B)** installation of PFA coil holder in the Vapourtec System.

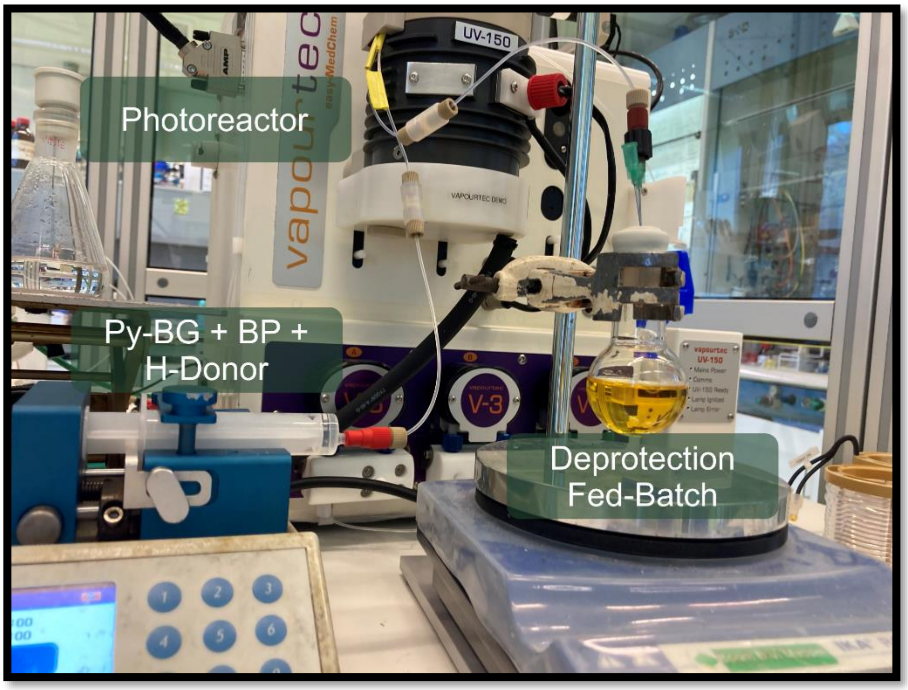

Figure S2: Overview of the reaction setup using the fed-batch approach.

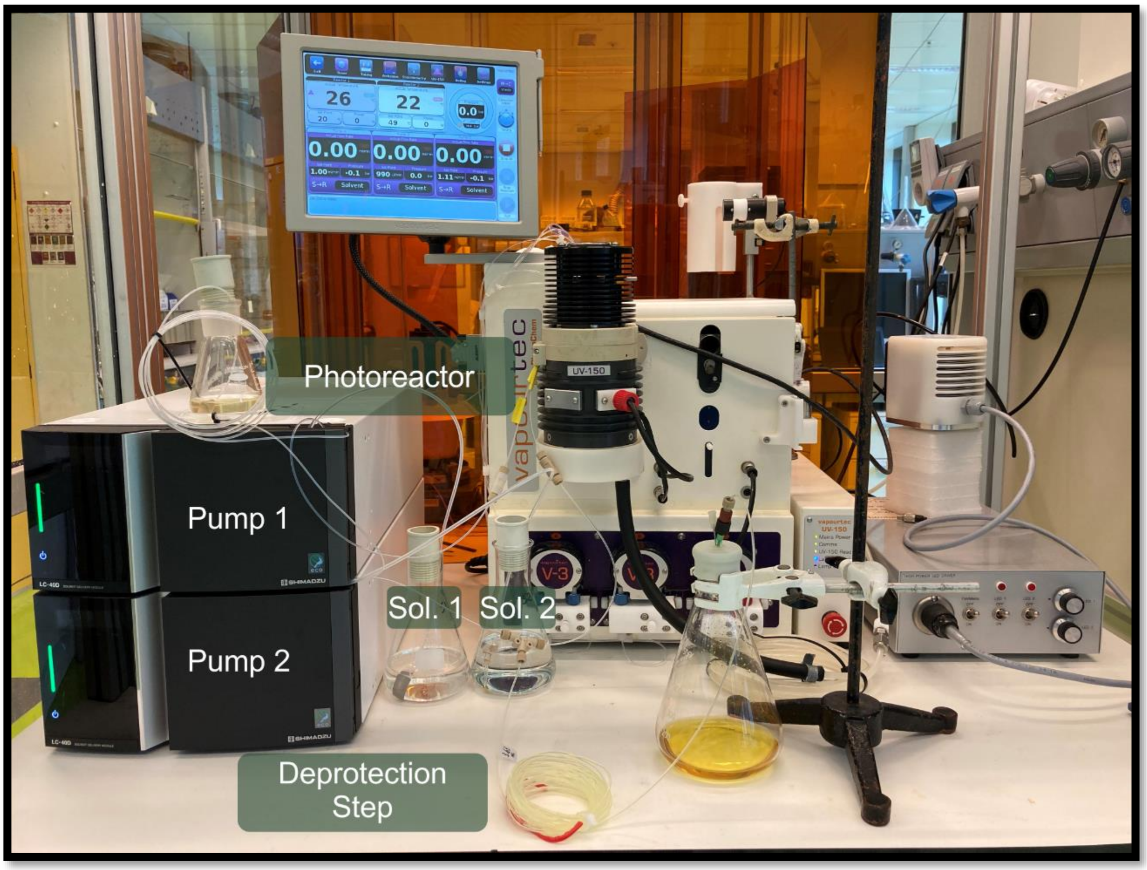

Figure S3: Overview of the reaction setup using the telescope approach. Sol. 1: pyridine blocking group, H-Donor, and benzophenone in acetonitrile. Sol. 2: DBU in dichloromethane.

3. REACTION OPTIMIZATION

For the development of the herein described C-4 selective Minisci reaction, a screening of several reaction conditions that promotes the alkyl-aryl bond formation efficiently were tested. An initial solution, containing a pyridine bearing a fumarate-based blocking group (**1a**), cyclohexane (**2a**) as model alkane, a photocatalyst and an oxidant, was introduced into a syringe and pumped through the photoreactor displayed in Figure S1. The resulting reaction crude was collected in a vial after a given residence time, and the solvent was evaporated. The yield of the targeted product (**3a**) was calculated by ^{1}H NMR spectroscopy, using 1,3,5-trimethoxybenzene as an internal standard. The ^{1}H NMR signals used for yield determination were the aromatic protons from the pyridine ring for both starting material and product (Figure S4).

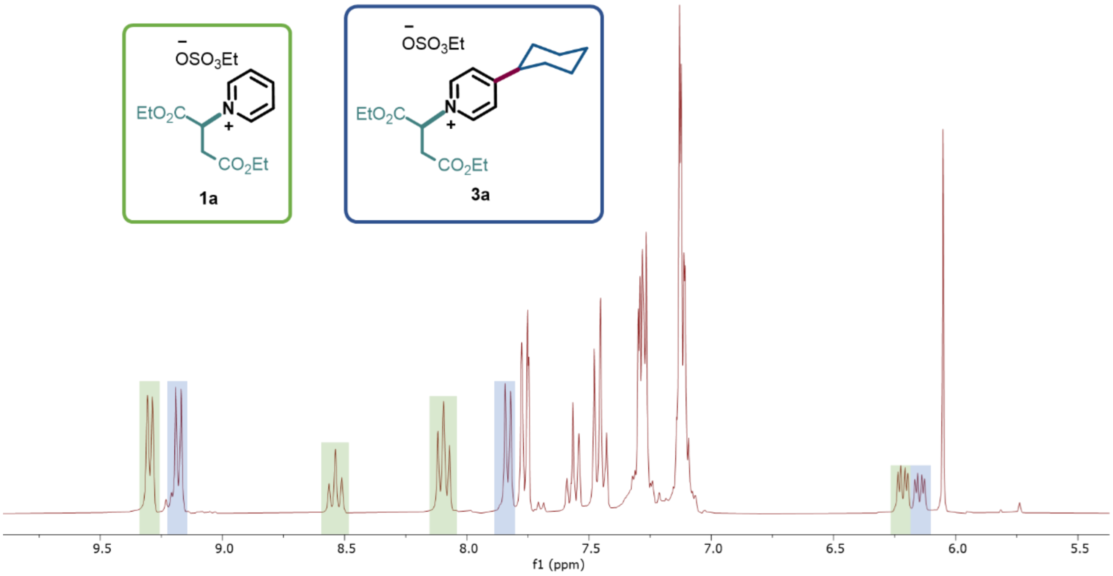

Figure S4: ^{1}H NMR spectrum of a reaction crude highlighting both starting material (in green) and product (in blue) characteristic peaks.

3.1. Photocatalyst and oxidant screening

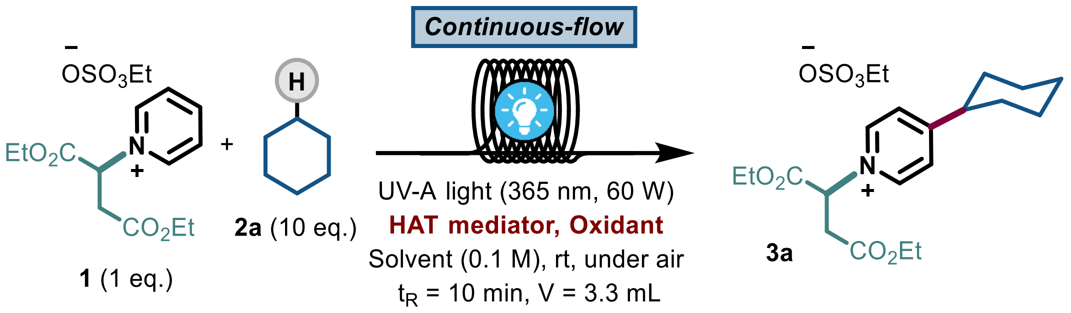

Entry	Oxidant (2 eq.)	HAT mediator	Solvent	Yield[a] (%)
1	$(NH_4)_2S_2O_8$	TBADT (0.04 eq.)	$CH_3CN:H_2O$ (1:1)	24
2	$(NH_4)_2S_2O_8$	BP1 (0.2 eq.)	$CH_3CN:H_2O$ (1:1)	28
3	$Ph(I)OAc_2$	BP1 (0.2 eq.)	CH_3CN	38
4	DMP	BP1 (0.2 eq.)	$CH_3CN:CH_2Cl_2$ (2.6:1)	27
5	Selectfluor	BP1 (0.2 eq.)	$CH_3CN:H_2O$ (1:1)	42
6		BP1 (0.2 eq.)	CH_3CN	13
7			CH_3CN	0[b]

[a] Yield determined by quantitative ^{1}H NMR using 1,3,5-trimethoxybenzene as an internal standard. DMP = Dess-Martin Periodinane, TBADT = tetrabutylammonium decatungstate. BP1 = benzophenone

First, we tested different combinations of stoichiometric oxidants and photocatalysts. The most used oxidant in Minisci-type transformations, i.e. $(NH_4)_2S_2O_8$ gave rise to solubility problems, as precipitation was observed during the reaction evolution, even when using water as a cosolvent. Therefore we switched to other organic oxidants, such as $Ph(I)OAc_2$, DMP or Selectfluor. On the side of the photocatalysts, an aromatic ketone such as benzophenone (**BP1**) demonstrated to be, at least, equally efficient as TBADT, so we adopted **BP1** as the photocatalyst. More interestingly, a blank experiment without external oxidant also furnished the targeted product **3a,** albeit in low yield.

Due to this unexpected dual role of the benzophenone, both as hydrogen atom transfer mediator and terminal oxidant (entry 6), we next investigated the effect of increasing the equivalents of **BP1**.

3.2. Equivalents of Benzophenone

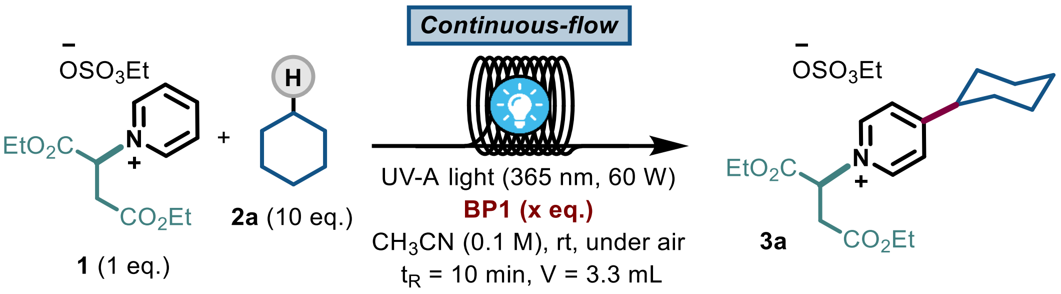

Entry	BP1 (eq.)	Yield[a] (%)
1	0.2	13
2	1	63
3	1.5	70
4	2.0	65

[a] Yield determined by quantitative ^{1}H NMR using 1,3,5-trimethoxybenzene as an internal standard.

After finding that sub-stoichiometric amounts of benzophenone can promote the Minisci reaction, we decided to investigate the effect of using stoichiometric amounts. Using 1 equivalent gave rise the desired coupling product **3a** in 63% qNMR yield. We could increase slightly the yield by using 1.5 equivalents of **BP1**, to 70%. However, when we used 2 equivalents, we started to observe the formation of by-products in the reaction mixture. Analysis of the different crudes by LC-MS demonstrated that overalkylation of **3a** took place when increasing the amount of **BP1** (Figure S5).

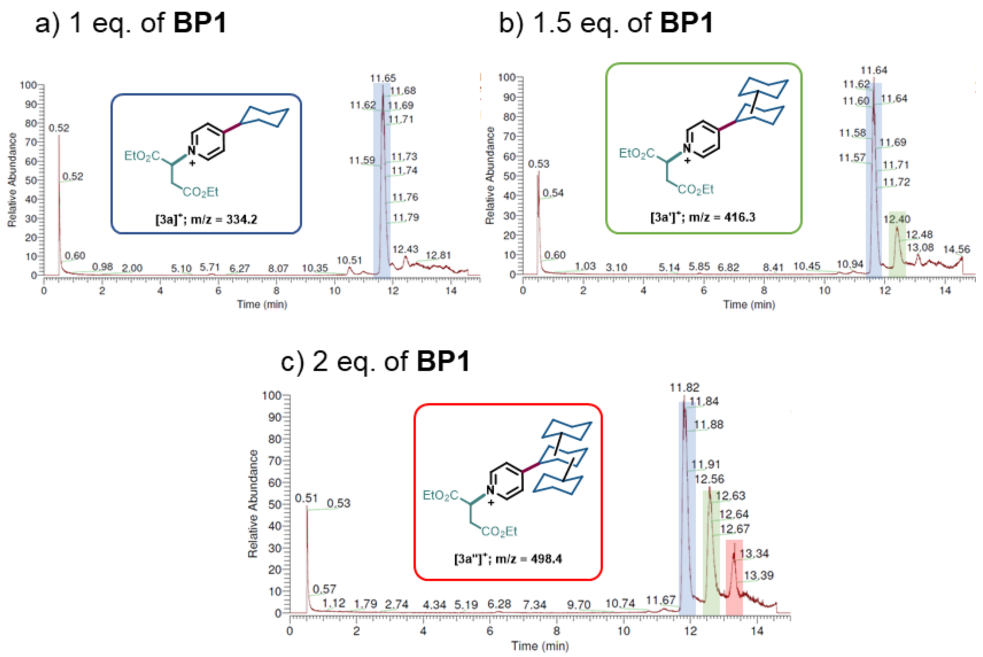

Figure S5: LC-MS measurements of the different reaction crudes.

3.3. Benzophenones screening

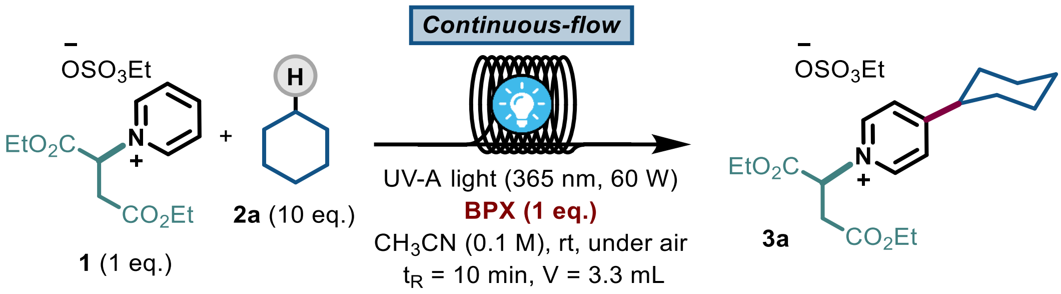

Entry	Benzophenone (BPX)	Yield[a] (%)
1	BP1	63
2	BP2	36
3	BP3	52
4	BP4	55

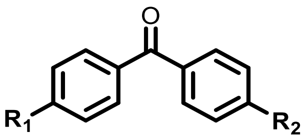

BP1, $R_1 = R_2 = H$
BP2, $R_1 = R_2 = MeO$
BP3, $R_1 = R_2 = CF_3$
BP4, $R_1 = MeO$; $R_2 = CF_3$

[a] Yield determined by quantitative ^1H NMR using 1,3,5-trimethoxybenzene as an internal standard.

Different substituted benzophenones were also tested. When using an electron-rich benzophenone, **BP2**, the compound **3a** was obtained in 36% yield. An electron-poor benzophenone such as **BP3**, gave rise compound **3a** in 52% yield. Finally, **BP4** bearing an electron-withdrawing group and an electron-donating group at the same time, yielded the product **3a** in 55% yield. Overall, none of the substituted benzophenones outperformed the standard and cheap **BP1**.

3.4. Solvent screening

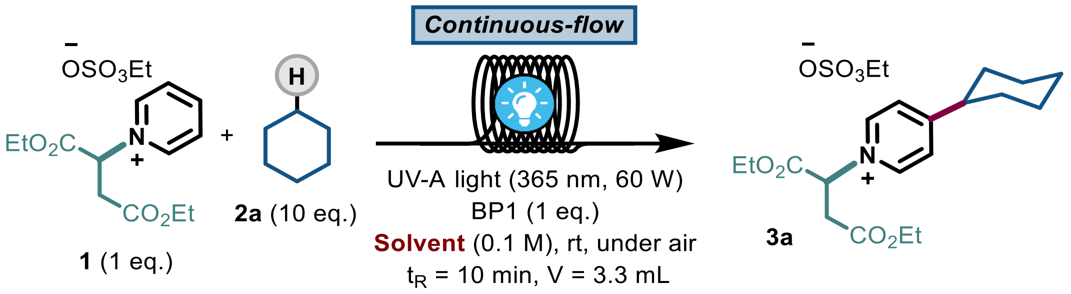

Entry	Solvent	Yield[a] (%)
1	CH_3CN	63
2	CH_3OH	32
3	CH_2Cl_2	54
4	$CHCl_3$	20
5	DCE	60
6	Acetone	57
7	$CH_3CN:H_2O$ (2:1)	44

[a] Yield determined by quantitative ^{1}H NMR using 1,3,5-trimethoxybenzene as an internal standard. DCE = dichloroethane.

The solvent screening of the reaction revealed that acetonitrile, dichloromethane, dichloroethane and acetone were promoting the formation of **3a** more efficiently, giving rise 63%, 54%, 60% and 57% yield, respectively. A significantly lower yield was achieved when using chloroform as the solvent of the reaction. More interestingly, the reaction demonstrated to be water-tolerant, observing a 44% formation of **3a** when using a biphasic mixture of acetonitrile/water (2:1).

3.5. Residence time optimization

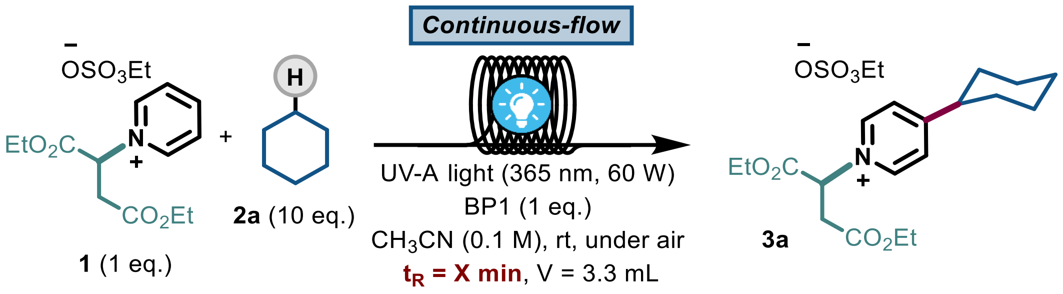

Entry	Residence time (min)	Yield[a] (%)
1	30	61
2	10	63
3	5	62
4	3	58
5	1	42

[a] Yield determined by quantitative ^{1}H NMR using 1,3,5-trimethoxybenzene as an internal standard.

Next, the residence time of the reaction inside the photochemical reactor was investigated. At 10 minutes of residence time, full conversion was observed with good yield. Longer residence time did not promote the degradation of the product and with shorter residence time than 5 minutes, lower conversion was observed.

3.6. Concentration optimization

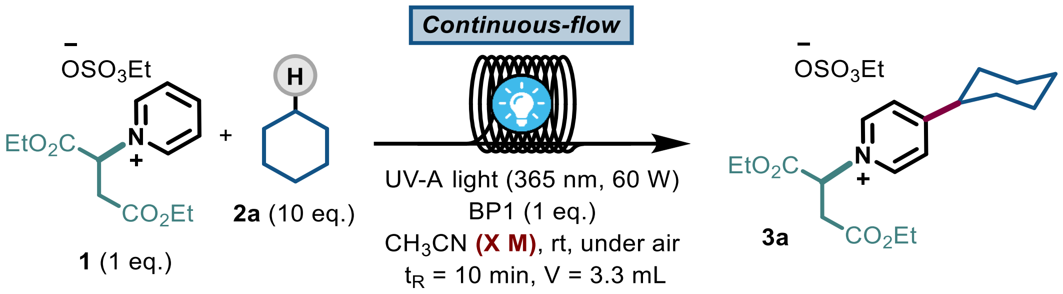

Entry	[1] (mol.L^{-1})	Yield[a] (%)
1	0.1	63
2	0.05	62
3	0.03	61
4	0.01	61

[a] Yield determined by quantitative ^{1}H NMR using 1,3,5-trimethoxybenzene as an internal standard.

We then investigated the effect of changing the concentration of **1** used in the reaction. Using higher concentration than 0.1 mol.L^{-1} gave rise immiscible mixtures, incompatible with our microfluidic setup. When more diluted solutions where tested, similar reaction yield was obtained.

3.7. H-Donor and photocatalyst equivalent correlation

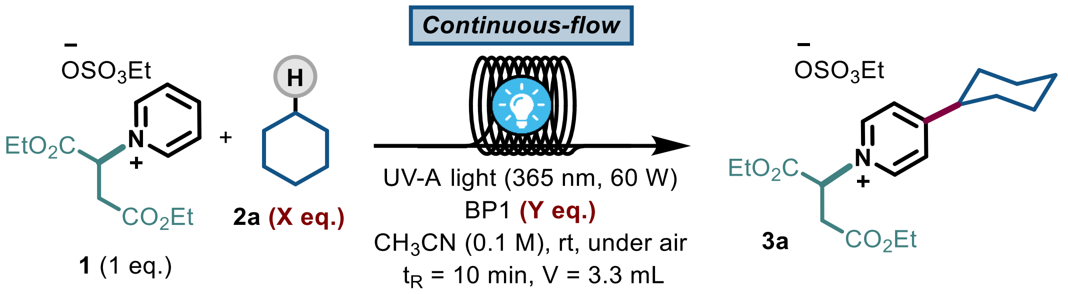

Entry	Cyclohexane (x eq.)	BP1 (x eq.)	Yield[a] (%)
1	15	1	64
2	10	1	63
3	5	1	65
4	2.5	1	55
5	1.5	1	41
6	2.5	1.5	66
7	1.5	1.5	50

[a] Yield determined by quantitative ^{1}H NMR using 1,3,5-trimethoxybenzene as an internal standard.

Furthermore, the required amount of alkane was investigated, and also the ratio of alkane/**BP1**. Using concentrations higher than 15 equivalents of **2a** afforded an immiscible solution. In addition, a slight decrease in yield was observed when 2.5 equivalents of **BP1** were used. However, when an excess of **BP1** (1.5 equivalents) and a low concentration of **2a** (2.5 equivalents) were used, the compound **3a** was obtained in 66% yield.

3.8. Optimization of the residence time for the deprotection step in the telescoped setup

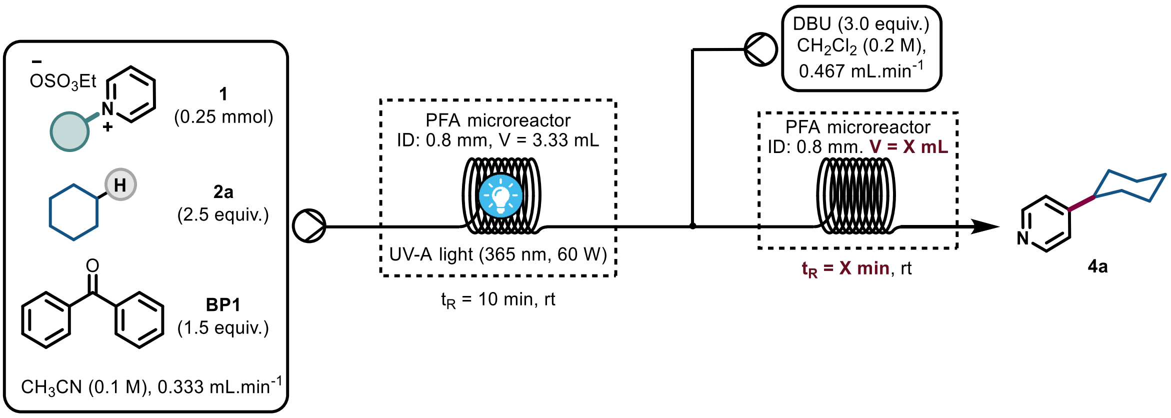

Entry	Coil Volume (mL)	Residence time (min)	Yield[a] (%)
1	0.8	1	54
2	4	5	62
3	8	10	64

[a] Yield determined by quantitative ^{1}H NMR using 1,3,5-trimethoxybenzene as an internal standard.

A mixture of pyridinium salt **1** (94.4 mg, 0.25 mmol), **BP1** (68.3 mg, 0.375 mmol, 1.5 eq.), **2a** (67 µL, 0.625 mmol, 2.5 eq.) in CH_3CN (0.1 M) was pumped through a PFA coil (ID: 0.8 mm, V = 3.33 mL, PFA = perfluoroalkoxy polymer) at a rate of 0.333 mL/min inside the photoreactor (Vapourtec, 365 nm, 60 W) at room temperature. The outflow of the latter was then mixed in a PEEK T-mixer with a solution of DBU (112 µL, 3 eq.) in CH_2Cl_2 (4.2 mL) pumped at 0.467 mL/min. For residence time screening of the deprotection step (second step) was used PFA coil (ID: 0.8 mm) volume between 0.8 and 8 mL. The resulting mixture was quenched in a vial with trifluoroacetic acid (5 eq.) in CH_3CN (3 mL).

4 SCOPE LIMITATIONS

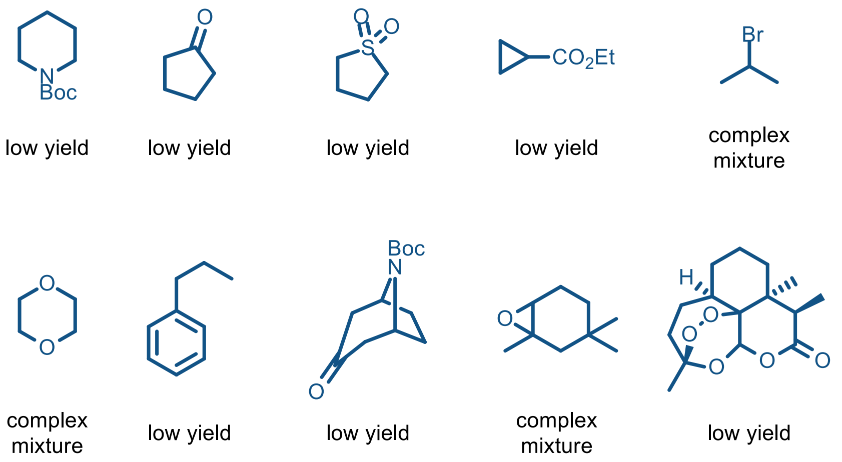

Herein we describe a list of some alkanes that were tested but unsuccessful under the developed reaction conditions. Either very low yields of complex mixtures were obtained, so further optimization of the methodology will be required in order to introduce a pyridine ring in these moieties.

5 SYNTHETIC PROCEDURES

5.1 Synthesis of pyridinium carboxylate salt:

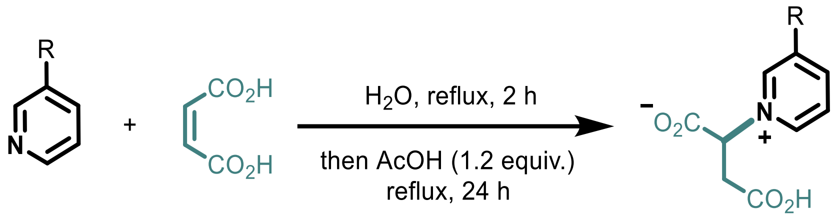

The synthesis of pyridinium carboxylate salt was performed following the procedure described in the literature.[1] To a solution of maleic acid (11.61 g, 100 mmol) in H_2O (50 mL) was added the corresponding pyridine (8.05 mL, 100 mmol, 1 eq.). The solution was stirred at 90 ºC for 2 h. After this time a crystalline solid precipitate in solution and acetic acid (6.9 mL, 120 mmol, 1.2 eq.) was added. The suspension was kept under stirring at 90 °C for 24 h. The crude reaction was cooled down to room temperature, filtered and the precipitate was washed with water, CH_3OH and EtOAc. The resulting white solid was dried overnight under reduced pressure and used for the next step without further purification.

5.2 Synthesis of substituted pyridinium salts (1a-1d):

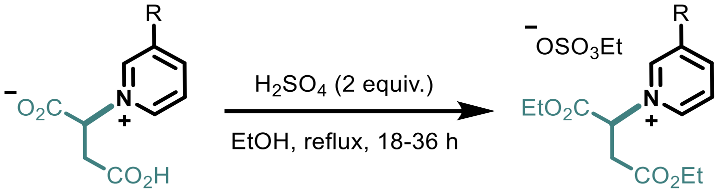

1a-1d

The synthesis of substituted pyridinium salts (**1a-1d**) were performed following the procedure described in the literature.[1] To a solution of pyridinium carboxylate

salt (1 eq.) in EtOH (0.2 M) was added concentrated sulfuric acid (2 eq.). The solution was stirred at 90 ºC for 18-36 h. The solvent was evaporated under reduced pressure and the crude reaction was diluted with a mixture of dichloromethane and H_2O (0.5 M, 9:1). The mixture was extracted 10 times with dichloromethane and the combined organic phase was dried by $MgSO_4$, filtered and the solvent evaporated under reduced pressure. The resulting product was dried overnight under reduced pressure and used for the next step without further purification. All NMR data of the compounds were in according to described in the literature.

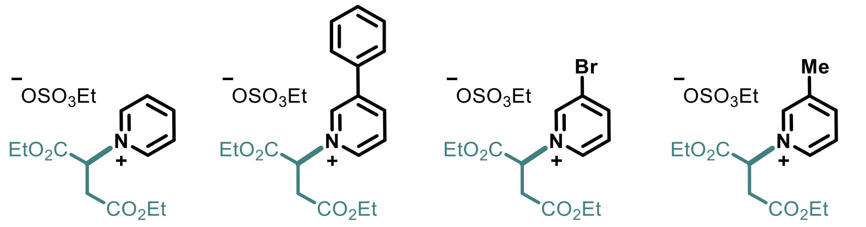

1a, 69% **1b,** 60% **1c,** 33% **1d,** 69%

5.3 General procedure A: Photoflow and Fed-batch deprotection

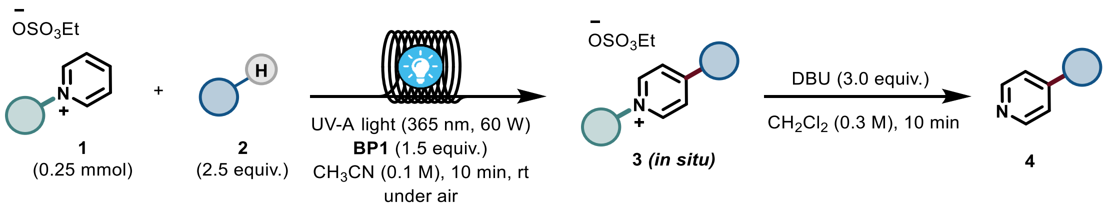

A mixture of pyridinium salt (**1**) (188.7 mg, 0.5 mmol), **BP1** (136.7 mg, 0.75 mmol, 1.5 eq.), H-donor (**2**) (2.5 eq.) in CH_3CN (0.1 M) was pumped through the coil (ID: 0.8 mm, V = 3.33 mL) to a rate of 0.333 mL/min by the photoreactor (Vapourtec, 365 nm, 60 W) to room temperature. The mixture was collected in a vial containing DBU (224 µL, 3 eq.) in CH_2Cl_2 (2.5 mL). Then, the solvent was evaporated and the product was isolated by a chromatography column.

5.4 General Procedure B: Telescope setup:

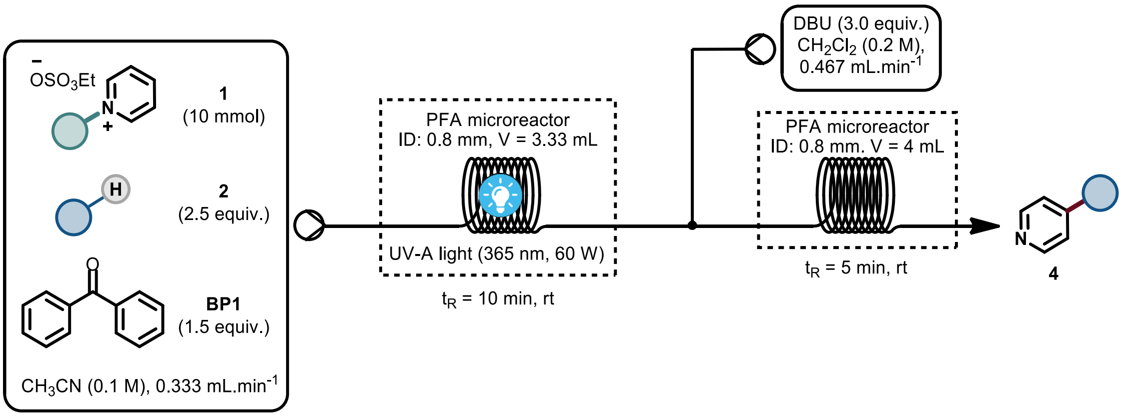

A mixture of pyridinium salt (**1**) (3.77 g, 10 mmol), **BP1** (2.73 g, 15 mmol, 1.5 eq.), H-donor (**2**) (2.5 eq.) in CH_3CN (0.1 M) was pumped through the PFA coil (ID: 0.8

mm, V = 3.33 mL) to a rate of 0.333 mL/min by the photoreactor (Vapourtec, 365 nm, 60 W) to room temperature. The outflow of the latter was then mixed in a PEEK T-mixer with a solution of DBU (4.5 mL, 3 eq.) in CH_2Cl_2 (168 mL) pumped at 0.467 mL/min by a PFA coil (ID: 0.8 mm, V = 4.00 mL) at room temperature. The solvent was evaporated under reduced pressure and the crude mixture was diluted in cyclohexane (100 mL) and aq. 1 M HCl (75 mL). The mixture was extracted in a separatory funnel and the aqueous phase was washed with cyclohexane (3 times x 50 mL). Then, the pH of the aqueous solution was adjusted above 10 with aq. 1 M NaOH and the mixture extracted with EtOAc (3 times x 50 mL). The combinate organic was dried with anhydrous $MgSO_4$, filtered and the solvent evaporated under reduced pressure.

6 EXPERIMENTAL DATA

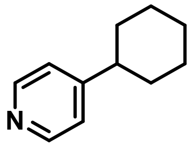

4a

4-cyclohexylpyridine (**4a**): Compound **4a** (reported compound)[1] was obtained following the general procedure A using cyclohexane (135 µL, 1.25 mmol, 2.5 eq.) as substrate. The compound **4a** was isolated by silica gel chromatography (DCM:MeOH = 99.5:0.5) and obtained as a yellow oil (50.1 mg, 61% yield).

5 mmol scale: The compound **4a** was obtained following the general procedure A using cyclohexane (1.35 mL, 2.5 equiv.) as substrate. The compound **4a** was isolated by silica gel chromatography (516.1 mg; 64% yield).

Gram scale: The compound **4a** was obtained following the general procedure B using cyclohexane (2.7 mL, 2.5 equiv.) as substrate. The compound **4a** was isolated by acid-base extraction (1.20 g, 74% yield, 91% purity).

^{1}H NMR (400 MHz, $CDCl_3$): δ 8.48 (bs, 2H); 7.11 (d, *J* = 5.2 Hz, 2H); 2.54 – 2.43 (m, 1H); 1.91 – 1.81 (m, 4H); 1.76 (d, *J* = 12.6 Hz, 1H); 1.39 (pent, *J* = 12.6 Hz, 4H); 1.31 – 1.21 (m, 1H).

^{13}C{H} NMR (100 MHz, $CDCl_3$): δ 156.6; 149.6; 122.4; 43.8; 33.5; 26.5; 25.9.

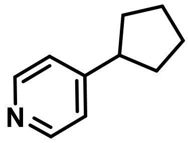

4b

4-cyclopentylpyridine (**4b**): Compound **4b** (reported compound)[2] was obtained following the general procedure A using cyclopentane (117 µL, 1.25 mmol, 2.5 equiv.) as substrate. The compound **4b** was isolated by silica gel chromatography (toluene:ethyl acetate = 95:5) and obtained as a yellow oil (43.0 mg, 58% yield).

^{1}H NMR (400 MHz, $CDCl_3$): δ 8.42 (bs, 2H); 7.09 (d, *J* = 5.6 Hz, 2H); 2.91 (quin, *J* = 8.40 Hz, 1H); 2.08 – 1.97 (m, 2H); 1.81 – 1.70 (m, 2H); 1.70 – 1.59 (m, 2H); 1.58 – 1.46 (m, 2H).

^{13}C{H} NMR (100 MHz, $CDCl_3$): δ 155.8; 149.4; 122.7; 45.2; 33.9; 25.5.

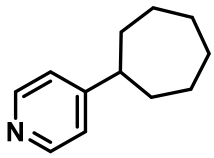

4c

4-cycloheptylpyridine (**4c**): Compound **4c** (reported compound)[3] was obtained following the general procedure A using cycloheptane (152 µL, 1.25 mmol, 2.5 equiv.) as substrate. The compound **4c** was isolated by silica gel chromatography (toluene:ethyl acetate = 95:5) and obtained as a yellow oil (58.7 mg, 67% yield).

^{1}H NMR (400 MHz, $CDCl_3$): δ 8.40 (d, *J* = 5.3 Hz, 2H); 7.03 (d, *J* = 6.0 Hz, 2H); 2.63 – 2.53 (m, 1H); 1.86 – 1.78 (m, 2H); 1.78 – 1.69 (m, 2H); 1.68 – 1.43 (m, 8H).

^{13}C{H} NMR (100 MHz, $CDCl_3$): δ 158.5; 149.7; 122.3; 46.2; 35.9, 27.8, 27.1.

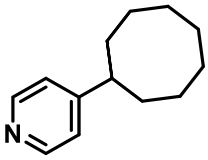

4d

4-cyclooctylpyridine (**4d**): Compound **4d** (reported compound)[4] was obtained following the general procedure A using cyclooctane (169 µL, 1.25 mmol, 2.5 equiv.) as substrate. The compound **4d** was isolated by silica gel chromatography (toluene:ethyl acetate = 95:5) and obtained as a yellow oil (66.9 mg, 71% yield).

^{1}H NMR (400 MHz, $CDCl_3$): δ 8.39 (d, *J* = 5.8 Hz, 2H); 7.03 (d, *J* = 5.9 Hz, 2H); 2.73 – 2.61 (m, 1H); 1.82 – 1.43 (m, 14H).

^{13}C{H} NMR (100 MHz, $CDCl_3$): δ 158.8; 149.7; 122.5; 44.0; 33.7; 26.8; 26.2; 25.8.

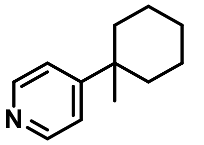

4e-a

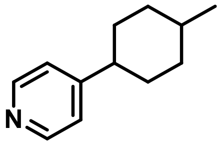

4e-b

4-(1-methylcyclohexyl)pyridine (**4e-a**) and 4-(4-methylcyclohexyl)pyridine (**4e-b**): The compounds **4e-a** and **4e-b** (reported compounds)[1] were obtained following the general procedure A using methylcyclohexane (160 µL, 1.25 mmol, 2.5 equiv.) as substrate. The compounds **4e-a** and **4e-b** were isolated as a yellow oil (55.2 mg, 63% yield) by silica gel chromatography (DCM:MeOH = 99.5:0.5) and obtained as an inseparable mixture of regioisomers (1:1 r.r.).

^{1}H NMR (400 MHz, $CDCl_3$): δ 8.63–8.39 (m, 3.4H); 7.29–7.25 (m, 2.4H); 7.18–7.05 (m, 1.8H); 2.87–2.75 (m, 0.1H); 2.52 (dt, *J* = 12.0, 3.2 Hz, 0.5H); 2.48–2.37 (m, 0.2H); 2.12–1.70 (m, 7.4H); 1.66–1.51 (m, 5.3H); 1.51–1.25 (m, 7H); 1.17 (s, 3H); 1.14–0.98 (m, 1.6H); 0.95 (d, *J* = 6.5 Hz, 1.9H); 0.66 (d, *J* = 6.5 Hz, 0.7H).

^{13}C{H} NMR (101 MHz, $CDCl_3$): δ 159.0; 155.6; 149.8; 149.7; 124.0; 121.0; 52.0; 43.7; 42.1; 38.0; 37.3; 37.2; 37.0; 35.4; 35.2; 34.9; 34.6; 33.4; 33.0; 32.9; 32.2; 31.5; 30.0; 26.5; 26.4; 26.3; 26.1; 22.7; 22.6; 22.4; 20.6.

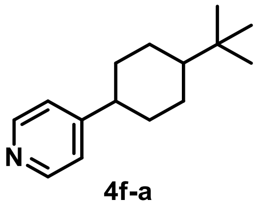

4f-a

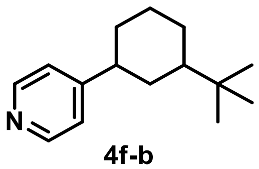

4f-b

4-(4-(*tert*-butyl)cyclohexyl)pyridine (**4f-a**) and 4-(3-(tert-butyl)cyclohexyl)pyridine (**4f-b**): The compounds **4f-a** and **4f-b** (unreported compounds) were obtained following the general procedure A using *tert*-butylcyclohexane (845 µL, 5 mmol,

10 equiv.) as substrate. The compounds **4f-a** and **4f-b** were isolated as a yellow oil (54.1 mg, 50% yield) by silica gel chromatography (DCM:MeOH = 99.5:0.5) and obtained as an inseparable mixture of diastereomers (2.3:1 r.r., respectively).

^{1}H NMR (400 MHz, $CDCl_3$): δ 8.48 (bs, 2H); 7.15–7.07 (m, 2H); 2.47 (dt, J = 11.6, 3.3 Hz, 0.8H); 2.42 (dt, J = 12.1, 3.1 Hz, 0.2H); 1.97–1.79 (m, 4H); 1.46–1.24 (m, 3H); 1.22–0.93 (m, 3.7H); 0.87 (s, 2.5H); 0.86 (s, 6H); 0.79 (s, 0.8H); 0.77 (s, 0.5H).

^{13}C{H} NMR (100 MHz, $CDCl_3$): δ 156.6; 156.4; 154.1; 149.6; 149.6; 149.6; 149.5 142.0; 129.3; 128.5; 128.3; 126.8; 126.5; 122.4; 48.1; 48.0; 47.5; 44.3; 43.8; 42.1; 36.6; 35.9; 34.7; 33.8; 33.2; 32.5; 32.4; 30.4; 29.8; 28.9; 27.5; 27.5; 27.4; 27.3; 27.2; 26.7; 26.6.

HRMS (ESI): *m/z* calcd for $C_{15}H_{23}N_1$: 218.1909; found: 218.1913.

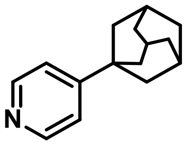

4g-a

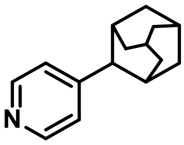

4g-b

4-(adamantan-1-yl)pyridine (**4g-a**) and 4-(adamantan-2-yl)pyridine (**4g-b**): The compounds **4g-a** and **4g-b** (reported compound)[1] were obtained following the general procedure A using adamantane (170.3 mg, 1.25 mmol, 2.5 equiv.) as substrate. The compounds were isolated as a yellow oil (67.0 mg, 63% yield) by silica gel chromatography (DCM:MeOH = 99.5:0.5), and obtained as an inseparable mixture of regioisomers (95:5 r.r., respectively).

^{1}H NMR (400 MHz, $CDCl_3$): δ 8.51 (d, J = 6.3 Hz, 2H); 7.23 (d, J = 6.3 Hz, 2H); 2.96 (s, 0.07); 2.46 (s, 0.14); 2.11 (bs, 3H);2.07–1.92 (m, 0.45H); 1.89 (bs, 6H); 1.78 (q, J = 12.6 Hz, 6H); 1.62–1.54 (m, 0.21H).

^{13}C{H} NMR (100 MHz, $CDCl_3$): δ 159.8, 149.7, 122.3, 120.3, 53.4, 46.4, 42.3, 38.8, 37.6, 36.5, 36.2, 31.9, 30.5, 28.7, 28.6, 27.8, 27.6.

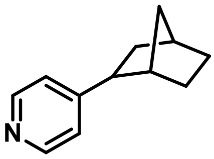

4h-a

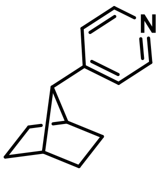

4h-b

4-(bicyclo[2.2.1]heptan-2-yl)pyridine (**4h-a**) 4-(bicyclo[2.2.1]heptan-7-yl)pyridine (**4h-b**): The compounds **4h-a** and **4h-b** (reported compound)[5] were obtained following the general procedure A using norbornane (120.2 mg, 1.25 mmol, 2.5 equiv.) as substrate. The compounds were isolated as a yellow oil (44.3 mg, 51% yield) by silica gel chromatography (toluene:ethyl acetate = 98:2), and obtained as an inseparable mixture of regioisomers (95:5 r.r., respectively).

^{1}H NMR (400 MHz, $CDCl_3$): δ 8.46 (d, J = 5.0 Hz, 2H); 7.10 (d, J = 5.0 Hz, 2H); 3.20–3.12 (m, 0.06H); 2.74–2.64 (m, 1H); 2.41–2.32 (m, 2H); 2.00–1.90 (m, 0.14H), 1.81–1.73 (m, 1H); 1.64–1.55 (m, 3H); 1.49–1.41 (m, 1H); 1.37–1.19 (m, 3H).

^{13}C{H} NMR (100 MHz, $CDCl_3$): δ 156.2, 149.6, 149.3, 123.6, 122.5, 46.6, 45.4, 42.5, 42.2, 42.0, 40.4, 38.5, 37.3, 36.7, 36.6, 36.1, 33.6, 30.7, 30.3, 29.9, 28.7, 22.9.

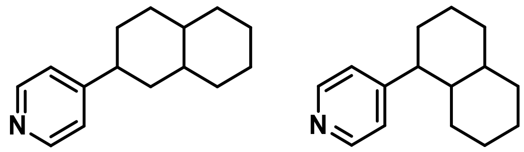

4i-a **4i-b**

4-(decahydronaphthalen-2-yl)pyridine (**4i-a**) and 4-(decahydronaphthalen-1-yl)pyridine (**4i-b**): The compound **4i-a** and **4i-b** (unreported compound) were obtained following the general procedure A using decahydronaphthalene (193 µL, 1.25 mmol, 2.5 equiv.) as substrate and 10 minutes of residence time. The compounds were isolated as a yellow oil (38.1 mg, 36% yield) by silica gel chromatography (pentane:ethyl acetate = 95:5 to 90:10), and obtained as an inseparable mixture of regioisomers (3:2 r.r., respectively).

^{1}H NMR (400 MHz, $CDCl_3$): δ 8.46 (bs, 4H); 7.10 (d, *J* = 5.4 Hz, 2H); 7.05 (d, *J* =5.4 Hz, 2H); 2.55 (tt, *J* = 12.1, 3.4 Hz, 1H); 2.16 (td, *J* = 10.9, 3.7 Hz, 1H); 1.91–1.54 (m, 15H); 1.50–1.35 (m, 4H); 1.34–0.90 (m, 18H); 0.74 (dq, *J* = 12.7, 3.7 Hz, 1H).

^{13}C{H} NMR (101 MHz, $CDCl_3$): δ 156.3, 155.4, 149.6, 149.6, 123.2, 122.3, 50.58, 47.2, 43.7, 43.1, 43.0, 42.8, 40.7, 35.1, 34.2, 34.0, 33.8, 33.8, 33.6, 33.3, 31.0, 26.6, 26.5, 26.5, 26.2.

HRMS (EI): *m/z* calcd for $C_{15}H_{21}N$: 215.1674; found: 215.1667.

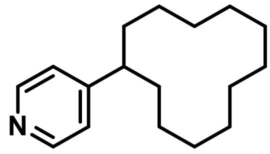

4j

4-cyclododecylpyridine (**4j**): The compound **4j** (reported compound) was obtained following the general procedure A using cyclododecane (210.4 mg, 1.25 mmol, 2.5 equiv.) as substrate. Compound **4j** was isolated by silica gel chromatography (DCM:MeOH = 99.5:0.5), and obtained as a yellow oil (48.1 mg, 39% yield).

^{1}H NMR (400 MHz, $CDCl_3$): δ 8.46 (bs, 2H); 7.09 (d, *J* = 4.7 Hz, 2H); 2.73 (p, *J* = 6.3 Hz, 1H); 1.84–1.71 (m, 2H); 1.53–1.14 (m, 20H).

^{13}C{H} NMR (101 MHz, $CDCl_3$): δ 156.4; 149.5; 123.2; 39.4; 30.7; 23.8; 23.6; 23.4; 23.2; 22.4.

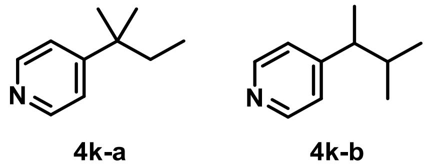

4k-a **4k-b**

4-(tert-pentyl)pyridine (**4k-a**) and 4-(3-methylbutan-2-yl)pyridine (**4k-b**): The compounds **4k-a** and **4k-b** (reported compounds)[6] were obtained following the general procedure A using isopentane (582 µL, 5 mmol, 10 equiv.) as substrate. The compounds **4k-a** and **4k-b** were isolated as a yellow oil (42.0 mg, 56% yield) by silica gel chromatography (DCM:MeOH = 99.5:0.5) and obtained as an inseparable mixture of regioisomers (9:1 r.r., respectively).

^{1}H NMR (400 MHz, $CDCl_3$): δ 8.49 (m, 2H); 7.20 (d, *J* = 5.9 Hz, 2H); 7.06 (d, *J* = 5.6 Hz, 0.2H); 2.40 (pent, *J* = 7.5 Hz, 0.1H); 1.76 (h, *J* = 6.8 Hz, 0.1H); 1.62 (q, *J* = 7.4 Hz, 2H); 1.25 (s, 6H); 1.20 (d, *J* = 7.0 Hz; 0.4H); 0.91 (d, *J* = 6.6 Hz, 0.4H); 0.74 (d, *J* = 6.7 Hz, 0.4H); 0.66 (t, *J* = 7.4 Hz, 3H).

^{13}C{H} NMR (101 MHz, $CDCl_3$): δ 158.5; 155.9; 149.4; 149.4; 123.2; 121.4; 46.2; 37.9; 36.1; 33.8; 27.6; 20.9; 19.8; 17.9; 8.9.

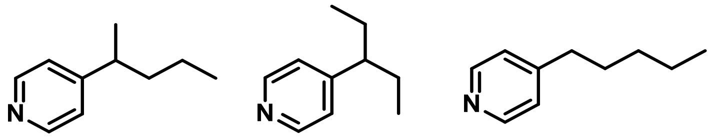

4l-a **4l-b** **4l-c**

4-(pentan-2-yl)pyridine (**4l-a**), 4-(pentan-3-yl)pyridine (**4l-b**) and 4-pentylpyridine (**4l-c**): The compounds **4l-a**, **4l-b**, and **4l-c** (unreported compounds) were obtained following the general procedure A using pentane (573 µL, 5 mmol, 10 equiv.) as substrate. The compounds were isolated as a yellow oil (33.8 mg, 45% yield) by silica gel chromatography (DCM:MeOH = 99.5:0.5) and obtained as an inseparable mixture of regioisomers (5:4:1 r.r., respectively).

^{1}H NMR (400 MHz, $CDCl_3$): δ 8.63–8.35 (m, 2.7H); 7.21 (d, *J* = 5.0 Hz, 0.2H); 7.09 (d, *J* = 5.0 Hz, 2H); 7.05 (d, *J* = 5.0 Hz, 0.8H); 2.67 (h, *J* = 7.1 Hz, 1H); 2.58 (t, *J* = 7.7 Hz, 0.1H); 2.35–2.23 (m, 0.4H); 1.75–1.60 (m, 1H); 1.59–1.48 (m, 2.6H); 1.31–1.11 (m, 6H); 0.86 (t, *J* = 7.3 Hz; 3H); 0.75 (t, *J* = 7.3 Hz, 2.1H); 0.66 (t, *J* = 7.6 Hz, 0.2H).

^{13}C{H} NMR (101 MHz, $CDCl_3$): δ 156.7; 154.9; 149.6; 149.5; 129.3; 128.5; 126.8; 123.4; 122.6; 49.1; 39.8; 39.1; 37.9; 36.2; 35.2; 31.3; 29.9; 28.5; 27.6; 22.4; 21.4; 20.5; 14.0; 13.9; 11.9; 8.9.

HRMS (ESI): *m/z* calcd for $C_{10}H_{15}N_1$: 150.1283; found: 150.1283

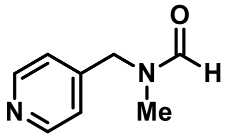

4m

N-methyl-N-(pyridin-4-ylmethyl)formamide (**4m**): The compound **4m** (reported compound)[7] was obtained following the general procedure A using N,N-dimethylformamide (389 µL, 5 mmol, 10 equiv.) as substrate. The compound **4m** was isolated by silica gel chromatography (DCM:MeOH = 99:1) and obtained as a yellow oil (32.3 mg, 43% yield).

^{1}H NMR (400 MHz, $CDCl_3$): δ 8.57, 8.53 (each bs, total 2H); 8.22, 8.16 (each s, total 1H); 7.12, 7.10 (each d, J = 5.9 Hz, total 2H); 4.48, 4.38 (each s, total 2H); 2.86, 2.77 (each s, total 3H).

^{13}C{H} NMR (100 MHz, $CDCl_3$): δ 162.8, 162.7; 150.2, 150.0; 145.0, 144.9; 129.2, 128.5; 122.7, 122.0; 52.2, 46.8; 34.2, 29.7.

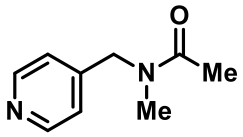

4n

N-methyl-*N*-(pyridin-4-ylmethyl)acetamide (**4n**): The compound **4n** (unreported compound) was obtained following the general procedure A using N,N-dimethylacetamide (116 µL, 1.25 mmol, 2.5 equiv.) as substrate. The compound **4n** was isolated by silica gel chromatography (DCM:MeOH = 99:1) and obtained as a yellow oil (45.5 mg, 55% yield).

^{1}H NMR (400 MHz, $CDCl_3$): δ 8.59, 8.53 (each d, *J* = 5.5 Hz, total 2H); 7.11, 7.08 (each d, *J* = 5.5 Hz, total 2H); 4.55, 4.50 (each s, total 2H); 2.94, 2.93 (each s, total 3H); 2.16, 2.07 (each s, total 3H).

^{13}C{H} NMR (100 MHz, $CDCl_3$): δ 170.9; 150.4, 150.0; 146.3, 145.8; 122.6, 121.1; 53.3, 49.9; 36.0, 34.0; 21.6, 21.3.

HRMS (ESI): *m/z* calcd for $C_9H_{12}N_2O_1$: 165.1028; found: 165.1025.

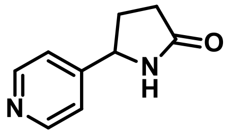

4o

5-(pyridin-4-yl)pyrrolidin-2-one (**4o**): The compound **4o** (unreported compound) was obtained following the general procedure A using pyrrolidin-2-one (96 µL, 1.25 mmol, 2.5 equiv.) as substrate. The compound **4o** was isolated by silica gel chromatography (pentane:DCM:MeOH = 4:5.5:0.5), and obtained as a brown solid (43.2 mg, 53% yield).

^{1}H NMR (400 MHz, $CDCl_3$): δ 8.54 (bs, 2H); 7.70 (bs, 1H); 7.19 (d, *J* = 5.3 Hz, 2H); 4.72 (t, *J* = 7.1 Hz, 1H); 2.64–2.53 (m, 1H); 2.42–2.35 (m, 2H); 1.93–1.83 (m, 1H).

^{13}C{H} NMR (100 MHz, $CDCl_3$): δ 179.1; 151.6; 150.1; 120.4; 56.9; 30.3; 29.9.

HRMS (ESI): *m/z* calcd for $C_9H_{10}N_2O_1$: 163.0871; found: 163.0866.

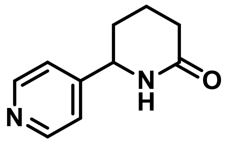

4p

6-(pyridin-4-yl)piperidin-2-one (**4p**) The compound **4p** (unreported compound) was obtained following the general procedure A using piperidin-2-one (123.9 mg, 1.25 mmol, 2.5 equiv.) as substrate. The compound **4p** was isolated by silica gel chromatography (DCM:MeOH = 99:1) and obtained as a yellow oil (48.6 mg, 55% yield).

^{1}H NMR (400 MHz, $CDCl_3$): δ 8.62 (bs, 2H); 7.24 (d, *J* = 5.0 Hz, 2H); 6.10 (bs, 1H); 4.58 (dd, *J* = 7.5; 5.5 Hz, 1H); 2.50–2.42 (m, 2H); 2.20–2.11 (m, 1H); 1.88–1.78 (m, 2H); 1.73–1.66 (m, 1H).

^{13}C{H} NMR (100 MHz, $CDCl_3$): δ 172.3; 151.4; 150.3; 121.0; 56.6; 31.4; 31.3; 19.2.

HRMS (ESI): *m/z* calcd for $C_{10}H_{12}N_2O_1$: 177.1028; found: 177.1029.

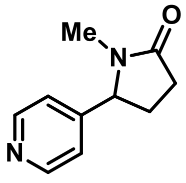

4q

1-methyl-5-(pyridin-4-yl)pyrrolidin-2-one (**4q**): The compound **4q** (unreported compound) was obtained following the general procedure A using 1-methylpyrrolidin-2-one (120 µL, 1.25 mmol, 2.5 equiv.) as substrate. Compound **4q** was isolated by silica gel chromatography (pentane:DCM:MeOH = 4:5.5:0.5) and obtained as a yellow oil (48.6 mg, 55% yield).

^{1}H NMR (400 MHz, $CDCl_3$): δ 8.57 (bs, 2H); 7.08 (d, *J* = 5.3 Hz, 2H); 4.47 (t, *J* = 6.4 Hz, 1H); 2.66 (s, 3H); 2.52–2.38 (m, 3H); 1.86–1.73 (m, 1H).

^{13}C{H} NMR (100 MHz, $CDCl_3$): δ 175.4; 150.4; 150.1; 121.1; 63.4; 29.5; 28.3; 27.7.

HRMS (ESI): *m/z* calcd for $C_{10}H_{12}N_2O_1$: 177.1028; found: 177.1029.

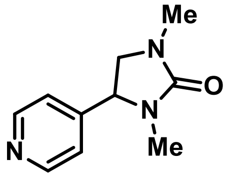

4r

1,3-dimethyl-4-(pyridin-4-yl)imidazolidin-2-one (**4r**): The compound **4r** (unreported compound) was obtained following the general procedure A using 1,3-dimethylimidazolidin-2-one (135 µL, 1.25 mmol, 2.5 equiv.) as substrate. The compound **4r** was isolated by silica gel chromatography (DCM:MeOH = 99.5:0.5 → 98:2), and obtained as a colorless oil (21.8 mg, 23% yield).

^{1}H NMR (400 MHz, $CDCl_3$): δ 8.61 (bs, 2H); 7.22 (d, *J* = 5.7 Hz, 2H); 4.36 (t, *J* = 8.7 Hz, 1H); 3.67 (t, *J* = 8.7 Hz, 1H); 3.01 (t, *J* = 8.7 Hz, 1H); 2.81 (s, 3H); 2.64 (s, 3H).

^{13}C{H} NMR (100 MHz, $CDCl_3$): δ 161.5; 150.4; 148.6; 121.7; 59.5; 53.3; 31.2; 29.9.

HRMS (ESI): *m/z* calcd for $C_{10}H_{13}N_3O_1$: 192.1137; found: 192.1135.

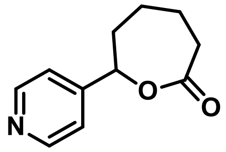

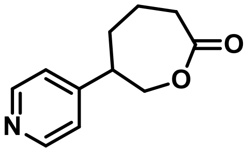

4s-a **4s-b**

7-(pyridin-4-yl)oxepan-2-one (**4s-a**) and 6-(pyridin-4-yl)oxepan-2-one (**4s-b**): The compounds **4s-a** and **4s-b** (unreported compounds) were obtained following the general procedure A using oxepan-2-one (554 µL, 5 mmol, 10 equiv.) as substrate. The compounds **4s-a** and **4s-b** were isolated as a yellow oil (22.1 mg, 23% yield) by silica gel chromatography (DCM:MeOH = 99.5:0.5) and obtained as an inseparable mixture of regioisomers (1:1 r.r.).

^{1}H NMR (400 MHz, $CDCl_3$): δ 8.63–8.45 (m, 2H); 7.15–7.06 (m, 2H); 4.43 – 4.35 (m, 1H); 4.35–4.25 (m, 1H); 3.10–2.70 (m, 3H); 2.18–1.76 (m, 4H).

^{13}C{H} NMR (100 MHz, $CDCl_3$): δ 175.0; 173.6; 153.8; 153.3; 150.2; 150.1; 122.0; 121.6; 68.9; 67.6; 46.3; 40.4; 39.8; 36.8; 35.8; 33.3; 29.4; 28.7.

HRMS (ESI): *m/z* calcd for $C_{11}H_{13}N_1O_1$: 192.1025; found: 192.1023.

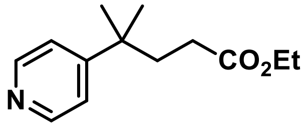

4t

Ethyl 4-methyl-4-(pyridin-4-yl)pentanoate (**4t**): The compound **4t** (unreported compound) was obtained following the general procedure A using ethyl 4-methylpentanoate (829 µL, 5 mmol, 10 equiv.) as substrate and 30 minutes of residence time. Compound **4t** was isolated by silica gel chromatography (DCM:MeOH = 99.5:0.5), and obtained as a yellow oil (42.5 mg, 38% yield).

^{1}H NMR (400 MHz, $CDCl_3$): δ 8.50 (bs, 2H); 7.20 (d, *J* = 4.6 Hz, 2H); 4.01 (q, *J* = 7.1 Hz, 2H); 2.04 – 1.90 (m, 4H); 1.27 (s, 6H); 1.17 (t, *J* = 7.1 Hz, 3H).

^{13}C{H} NMR (101 MHz, $CDCl_3$): δ 173.3; 157.2; 149.7; 121.2; 60.3; 38.0; 37.2; 29.9; 27.8; 14.0.

HRMS (ESI): *m/z* calcd for $C_{13}H_{19}N_1O_2$: 222.1494; found: 222.1490.

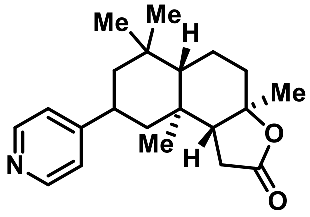

4u

(3aR,5aS,9aS,9bR)-3a,6,6,9a-tetramethyl-8-(pyridin-4-yl)decahydronaphtho[2,1-b]furan-2(3aH)-one (**4u**): The compound **4u** (unreported compound) was obtained following the general procedure A using sclareolide (313.0 mg, 1.25 mmol, 2.5 equiv.) as substrate. The compound **4u** was isolated by silica gel chromatography (DCM:MeOH = 99.5:0.5), and obtained as a white solid (45.1 mg, 28% yield).

^{1}H NMR (400 MHz, $CDCl_3$): δ 8.52 (bs, 2H); 7.13 (bs, 2H); 2.95 (tt, *J* = 12.7, 3.2 Hz, 1H); 2.47–2.36 (m, 1H); 2.22 (dd, *J* = 16.2, 6.4 Hz, 1H); 2.12 (dt, *J* = 11.9, 3.1 Hz, 1H); 2.04 (dd, *J* = 14.7, 6.4 Hz, 1H); 1.99–1.91 (m, 1H); 1.73 (td, *J* = 12.5, 4.0, 1H); 1.67–1.56 (m, 2H); 1.43–1.38 (m, 1H); 1.36 (s, 3H); 1.28–1.14 (m, 3H); 1.05 (s, 3H); 0.99–0.94 (m, 6H).

^{13}C{H} NMR (100 MHz, $CDCl_3$): δ 176.3; 154.9; 149.8; 122.5; 86.0; 58.9; 56.3; 48.9; 46.1; 38.6; 36.8; 35.1; 34.0; 33.0; 28.6; 21.6; 21.3; 20.4; 15.7.

HRMS (ESI): *m/z* calcd for $C_{21}H_{29}N_1O_2$: 328.2277; found: 328.2270.

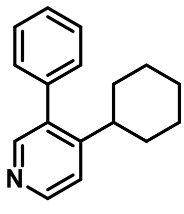

4v

4-cyclohexyl-3-phenylpyridine (**4v**): The compound **4v** (reported compound) was obtained following the general procedure A using compound **1b** (0.5 mmol) and cyclohexane (541 µL, 5 mmol, 10 equiv.) as substrate. The compound **4v** was isolated by silica gel chromatography (cyclohexane:ethyl acetate = 99:1 to 93:7) and obtained as a yellow oil (49.8 mg, 42% yield).

^{1}H NMR (400 MHz, $CDCl_3$): δ 8.49 (s, 1H); 8.38 (s, 1H); 7.50–7.35 (m, 3H); 7.30–7.19 (m, 2H, 1H); 2.65 (tt, *J* = 12.6, 12.1, 3.5 Hz, 2H); 1.76–1.62 (m, 5H), 1.46–1.33 (m, 2H), 1.29–1.08 (m, 3H).

$^{13}C\{H\}$ NMR (100 MHz, $CDCl_3$): δ 153.9; 150.3; 148.6; 138.0; 129.4; 128.5; 128.3; 127.5; 126.5; 121.3; 39.6; 33.8; 26.4; 25.9.

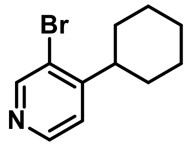

4w

3-bromo-4-cyclohexylpyridine (**4w**): The compound **4w** (reported compound) was obtained following the general procedure A using compound **1c** (0.5 mmol) and cyclohexane (135 µL, 1.25 mmol, 2.5 equiv.) as substrate. The compound **4w** was isolated by silica gel chromatography (pentane:ethyl acetate = 99:1 to 93:7) and obtained as a yellow oil (63.3 mg, 53% yield).

^1H NMR (400 MHz, $CDCl_3$): δ 8.64 (s, 1H); 8.41 (d, *J* = 5.1 Hz, 1H); 7.16 (d, *J* = 5.1 Hz, 1H); 2.91 (tt, *J* = 11.6, 3.1 Hz, 1H); 1.93–1.83 (m, 4H); 1.79 (d, *J* = 14.6 Hz, 1H); 1.52-1.19 (m, 6H).

$^{13}C\{H\}$ NMR (100 MHz, $CDCl_3$): δ 154.8; 151.8; 148.4; 123.0; 122.4; 42.8; 32.3; 26.4; 25.9.

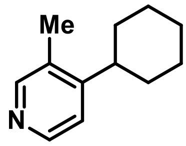

4x

4-cyclohexyl-3-methylpyridine (**4x**): The compound **4x** (reported compound) was obtained following the general procedure A using compound **1d** (0.5 mmol) and cyclohexane (135 µL, 1.25 mmol, 2.5 equiv.) as substrate. The compound **4x** was isolated by silica gel chromatography (pentane:ethyl acetate = 99.5:0.5) and obtained as a yellow oil (42.0 mg, 48% yield).

^1H NMR (400 MHz, $CDCl_3$): δ 8.35 (d, 1H, 1H); 7.10 (d, *J* = 5.1 Hz, 1H); 2.67 (tt, *J* = 11.0, 3.1 Hz, 1H); 2.29 (s, 3H); 1.93–1.75 (m, 6H); 1.49–1.32(m, 4H); 1.31–1.22 (m, 1H).

$^{13}C\{H\}$ NMR (100 MHz, $CDCl_3$): δ 154.4; 150.6; 147.7; 130.7; 39.8; 32.7; 26.8; 26.1; 16.0.

7 EXPERIMENTAL MECHANISTIC STUDIES

In order to get information about the mechanistic intricacies of the benzophenone-mediated Minisci reaction, we designed a couple of kinetic experiments. Due to the quick reaction times under standard microfluidic conditions, we modified slightly the reaction conditions and conduct all the following mechanistic studies in batch, using a NMR tube inside a home-made batch reactor. This reactor consisted in a 3D-printed (PLA) vessel, internally coated with a reflectant layer, and equipped with a PR160L Kessil lamp (390 nm, 40 W).[8] On top of it, a 3D-printed (PLA) lid with 8 holes was mounted; in this way, up to 8 reactions could be run simultaneously. Cooling was applied via a strong compressed air flow from below to keep the temperature below 30 °C.

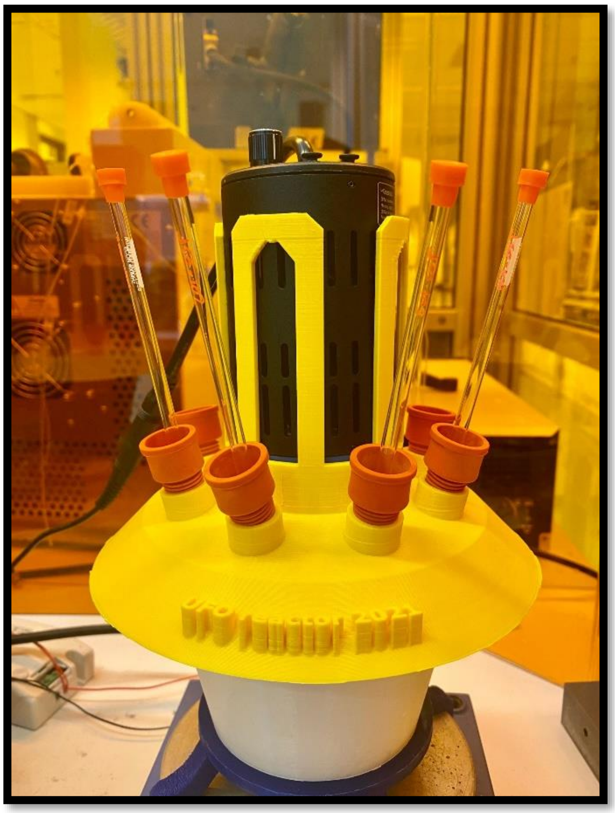

Figure S6. Picture of the 3D-printed batch reactor for the development of the kinetic studies.

All the following reactions in section 7 were monitored by ^{1}H NMR spectroscopy and the data points were acquired as following: after an initial spectrum of the sample without light irradiation, the sample was irradiated for a short given amount of time, and the evolution was analyzed by ^{1}H NMR spectroscopy. This procedure was repeated as many times as required in order to get the reaction profiles.

In order to test the viability of this procedure, and to check that the reaction only takes place under UV irradiation, an ON/OFF experiment was conducted next.

7.2 ON-OFF experiment

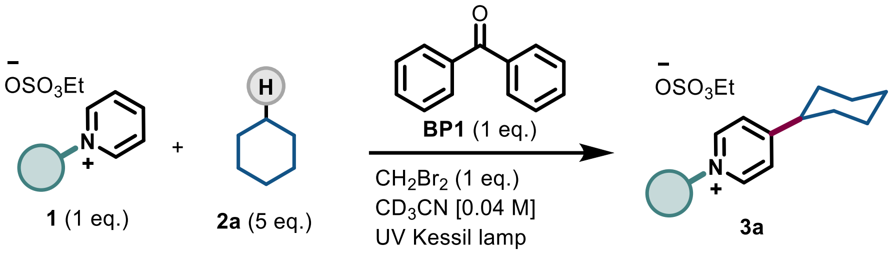

A NMR tube containing **1** (7.5 mg, 0.02 mmol), **2a** (10.8 μL, 5 eq.), **BP1** (3.6 mg, 1 eq.) and internal standard CH_2Br_2 (1.4 μL, 0.02 mmol) in 0.5 mL of CD_3CN was prepared. The different spectra were acquired at the described times in Figure S7, turning ON/OFF the Kessil lamp when required. No evolution towards the product was observed in absence of UV light.

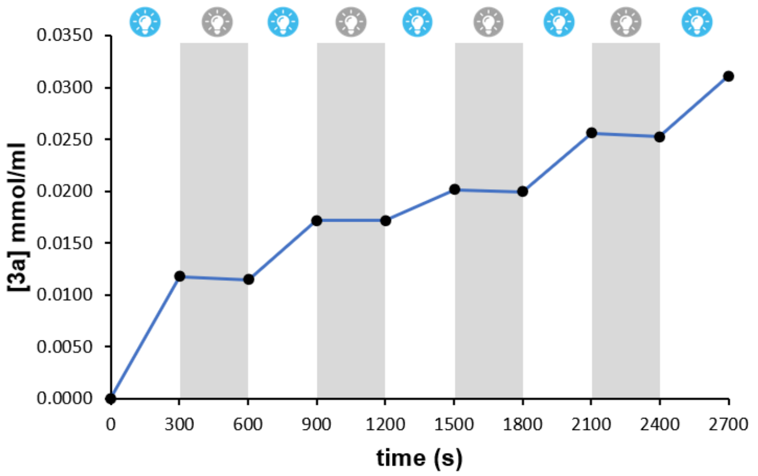

Figure S7. ON/OFF experiment profile.

7.3 Kinetic Orders

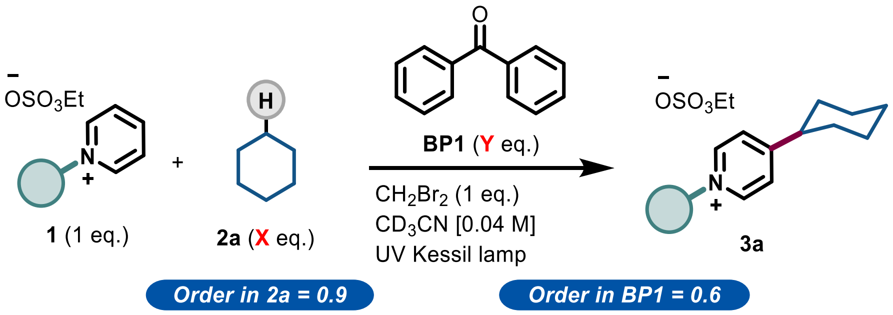

A stock solution containing **1** (75 mg, 0.1 mmol), internal standard CH_2Br_2 (15 μL, 0.1 mmol) in 5 mL of CD_3CN was prepared for all the following experiments. A 0.5 mL aliquot of the stock solution was then added to every NMR tube, already loaded with different amounts of **2a** (Table S1) or **BP1** (Table S2). The reaction mixtures were then inserted into the home-made photochemical batch reactor (Figure S6) and irradiated for a given amount of time. The evolution of the reactions were monitored directly by ^{1}H NMR, in comparison with the internal standard. In order to obtain the experimental reaction rates (r_0, Tables S1 and S2), the initial rates method was applied, for which only the first ~30% of conversion towards product was taken into account (Figures S8 and S10).

a) Order in Alkane

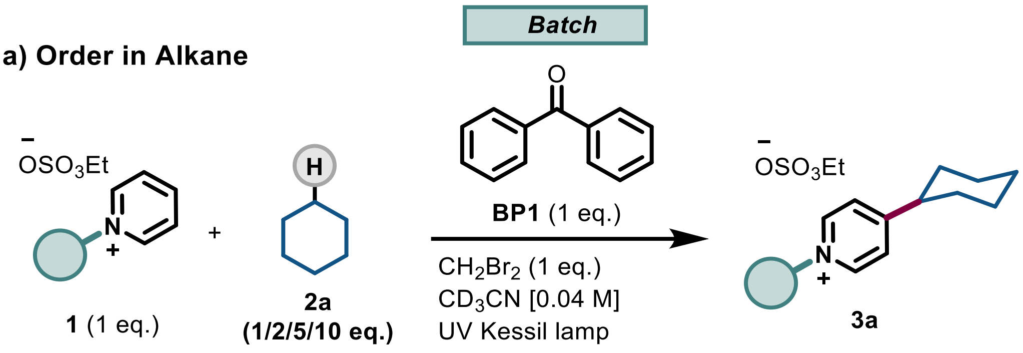

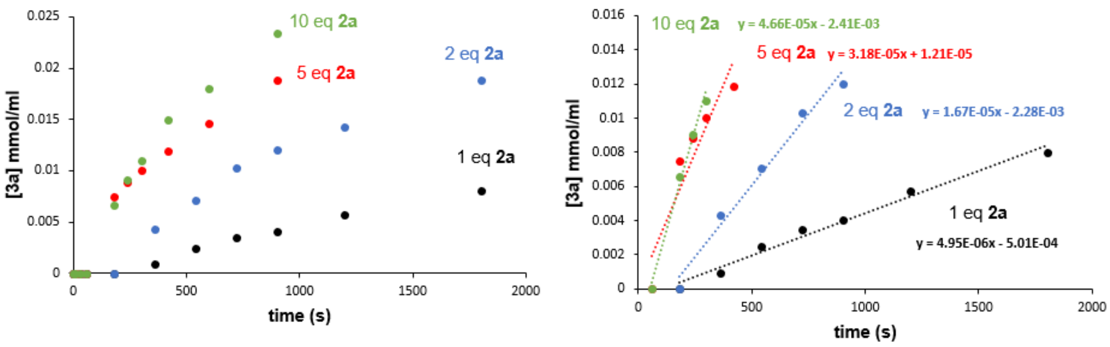

Figure S8. Concentration vs time plots for the formation of **3a**. Starting conditions $[\mathbf{1}]_0$ = 0.04 M, $[\mathbf{BP1}]_0$ 0.04 M.

Table S1. Initial rates for the formation of **3a** in CD_3CN at room temperature.[a]

**2a** equivalents	$[\mathbf{2a}]_{added}$ mol L^{-1}	$r_0/10^{-5}$ mol L^{-1} s^{-1}
1 equiv / 2.2 μL	0.04	0.49
2 equiv / 4.3 μL	0.08	1.67
5 equiv / 10.8 μL	0.2	3.18
10 equiv / 21.6 μL	0.4	4.66

[a] Up to 30% conversion.

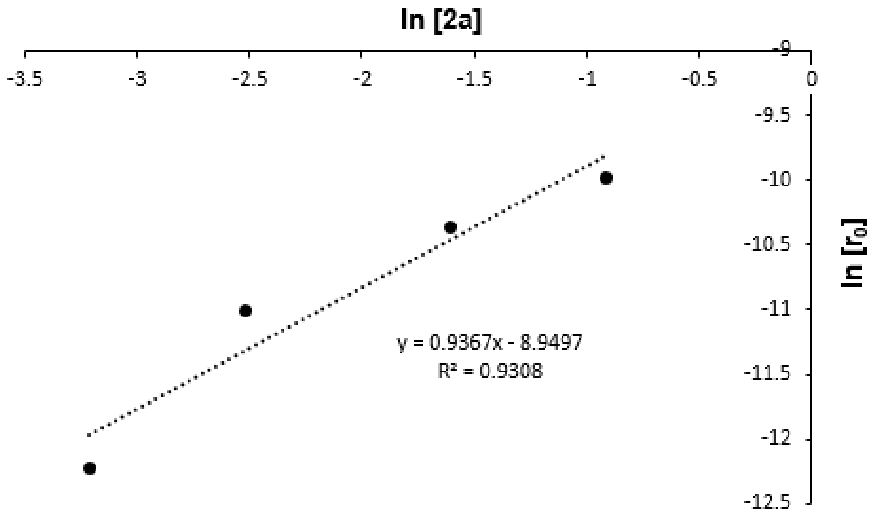

Figure S9. Plot of ln(r_0) vs. ln[**2a**]. The slope of the straight line is 0.9.

b) Order in Benzophenone

Batch

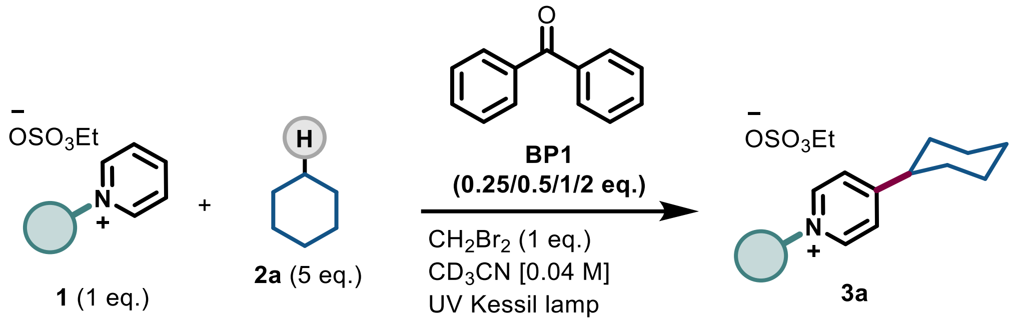

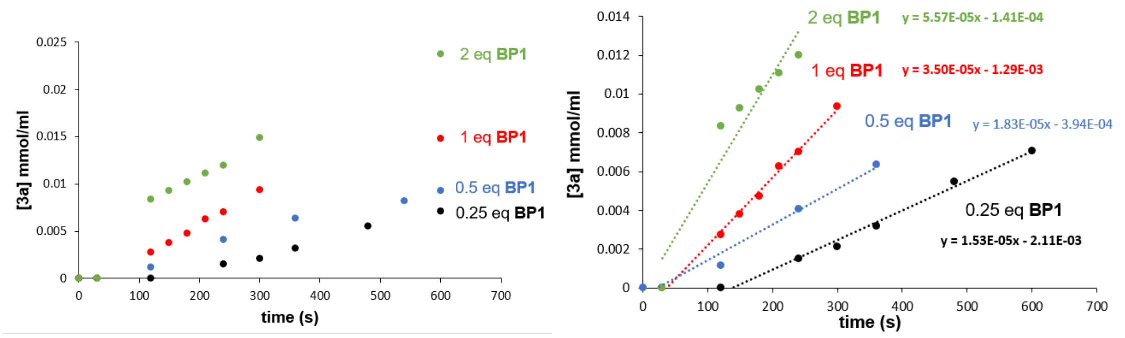

Figure S10. Concentration vs time plots for the formation of **3a**. Starting conditions [**1**]$_0$ = 0.04 M, [**2a**]$_0$ 0.20 M.

Table S2. Initial rates for the formation of **3a** in CD_3CN at room temperature.[a]

**BP1** equivalents	[**BP1**]$_{added}$ mol L^{-1}	$r_0/10^{-5}$ mol L^{-1} s^{-1}
0.25 equiv / 0.9 mg	0.01	1.53
0.5 equiv / 1.8 mg	0.02	1.83
1 equiv / 3.6 mg	0.04	3.50
2 equiv / 7.2 mg	0.08	5.57

[a] Up to 30 conversion.

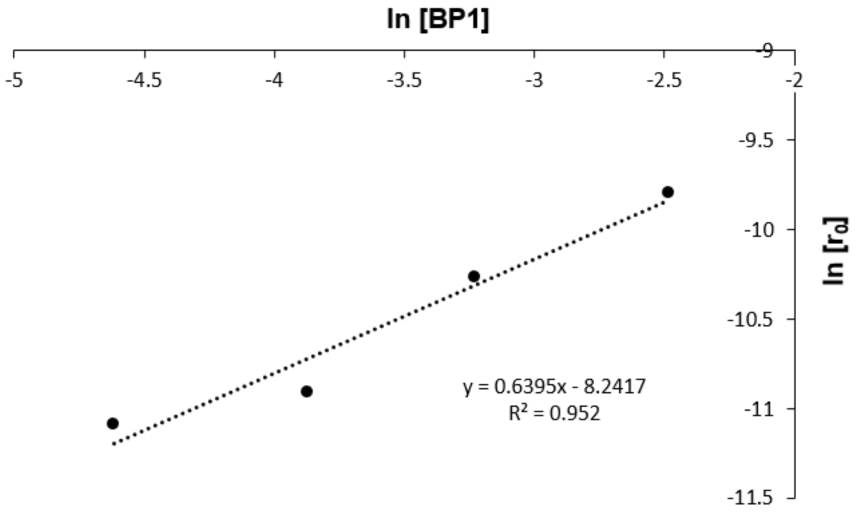

Figure S11. Plot of ln(r_0) vs. ln[**2a**]. The slope of the straight line is 0.6.

7.4 Kinetic Isotope Effect

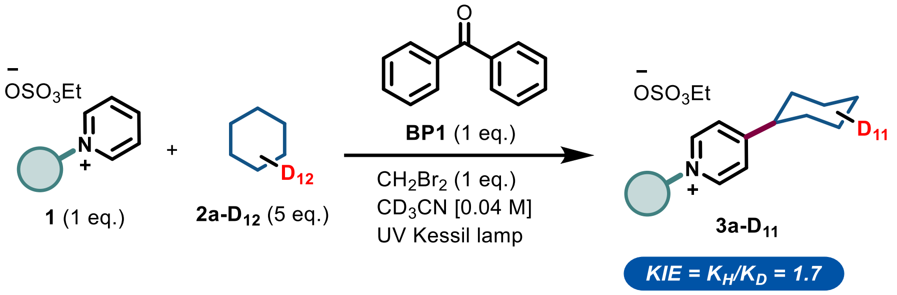

A stock solution containing **BP1** (14.4 mg, 0.03 mmol), internal standard CH_2Br_2 (5.3 µL, 0.03 mmol) in 2 mL of CD_3CN was prepared for the next experiments. A 0.5 mL aliquot of the stock solution was then added to every NMR tube, already loaded with the corresponding starting materials (Table S3). The reaction mixtures were then inserted into the home-made photochemical batch reactor (Figure S6) and irradiated for a given amount of time. The evolution of the reactions were monitored directly by ^{1}H NMR, in comparison with the internal standard. In order to obtain the experimental reaction rates (r_0, Table S3), the initial rates method was applied, for which only the first ~30% of conversion towards product was taken into account (Figure S12). By comparison of the different reactions rates with the reference reaction, the kinetic isotope effects could be calculated.

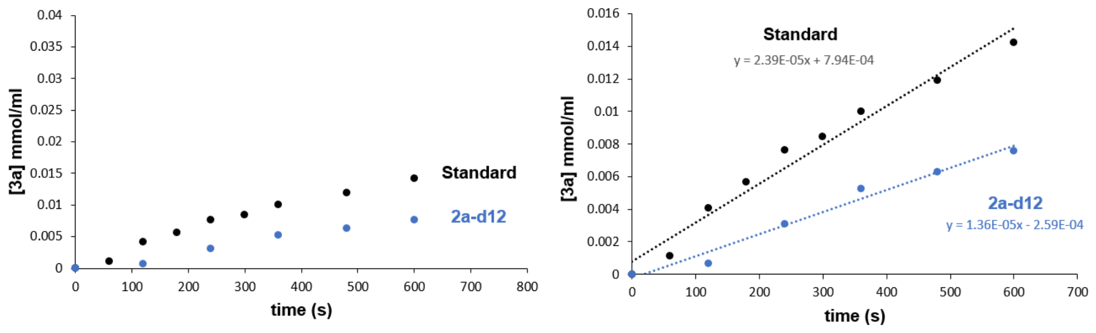

Figure S12. Concentration vs time plots for the formation of **3a**. Starting conditions [**1**]$_0$ = 0.04 M, [**2a**]$_0$ 0.20 M.

Table S3. Initial rates for the formation of **3a** in CD_3CN at room temperature.[a]

**Reaction**	[**1**]$_{added}$ mol L^{-1}	[**2a**]$_{added}$ mol L^{-1}	r_0/10^{-5} mol L^{-1} s^{-1}
Standard	0.04	0.2	2.39
**2a-*d*$_{12}$**	0.04	0.2 (**2a-D$_{12}$**)	1.43

[a] Up to 20% conversion.

$$KIE = K_H/K_D = 1.7$$

After comparing the initial rates of both reactions, we observed a small but not negligible kinetic isotope effect of 1.7. This might suggest that the C-H bond cleavage of the alkane is not rate-determining, but this elementary step could be in a prior equilibrium before the actual rate-determining step.

7.5 H/D scrambling

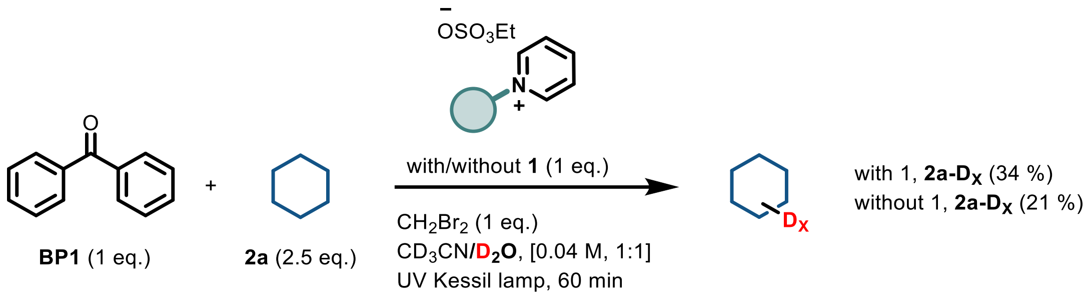

A stock solution containing **BP1** (10.8 mg, 0.06 mmol), **2a** (16.2 μL, 0.15 mmol), and internal standard CH_2Br_2 (4 μL, 0.06 mmol) in 1.5 mL of a 1:1 CD_3CN/D_2O mixture was prepared. 0.5 mL aliquots of this stock solution were then added to two NMR tubes, one already loaded with **1** (7.5 mg, 0.02 mmol). Both reaction mixtures were then inserted into a home-made photochemical batch reactor (Figure S6) and irradiated for 60 minutes. The deuteration of cyclohexane was monitored directly by ^1H NMR, in comparison with the internal standard and the dissappearence of the initial cyclohexane ^1H NMR peaks.

In both cases we could observe the partial deuteration of cyclohexane, which indicates that the first step of the reaction, the benzophenone-mediated hydrogen atom transfer, is reversible under the following conditions.

7.6 Oxygen Effect in Flow

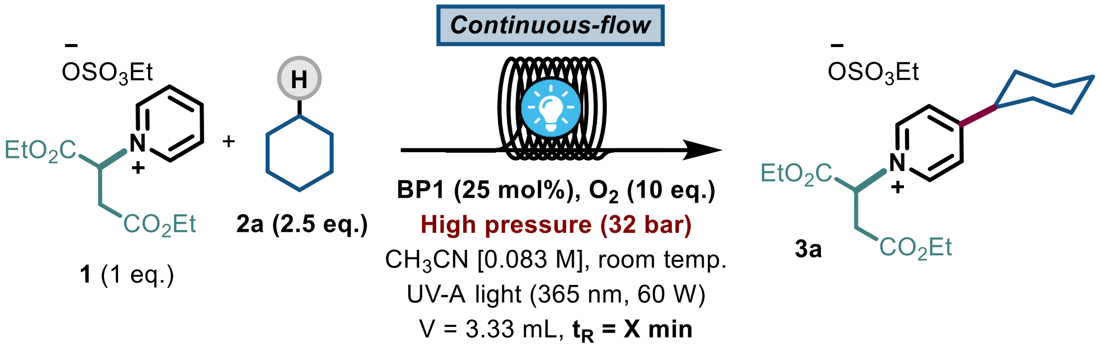

A stock solution containing **1** (94.4 mg, 0.25 mmol), **BP1** (11.4 mg, 25 mol%) and **2a** (68 µL, 0.625 mmol, 2.5 equiv.) in 3 mL of CH_3CN was prepared. To perform the reaction, the stock solution was first mixed with O_2 gas in a filling loop, after which the gas-liquid mixture was pumped over the photochemical reactor. For the loop filling, the stock solution was charged in a 5 mL syringe and the syringe was connected via a T-mixer with an oxygen line and a filling loop (PFA capillary: 0.8 mm ID, 10 mL), equipped with a switch valve on each side and a back-pressure regulator of 2.8 bar at the outlet (Figure S13A). Gas and liquid feed were pumped into the filling loop at 2.80 mL/min and 0.3 mL/min, respectively (10 equivalents of O_2), to create a gas-liquid slug flow. After all the solution had been fed into the filling loop, the two switch valves were closed, containing the gas-liquid mixture inside the loop. To flow the mixture through the reactor, the reactor coil was first filled with solvent, with a 34 bar BPR at the outlet. Then the filling loop was connected to the reactor coil on one side and to an HPLC pump on the other side (Figure S13B). After opening the switch valves, the content of the filling loop was pumped through the Vapourtec reactor with a flow rate of 0.333 mL·min^{-1} or 0.055 mL·min^{-1} to achieve 10 min and 1 h residence time, respectively. The outflow was collected, all solvent was removed through rotary evaporation, and the crude mixture was analyzed via ^{1}H NMR to determine the reaction yield (with 1,3,5-trimethoxybenzene as external standard).

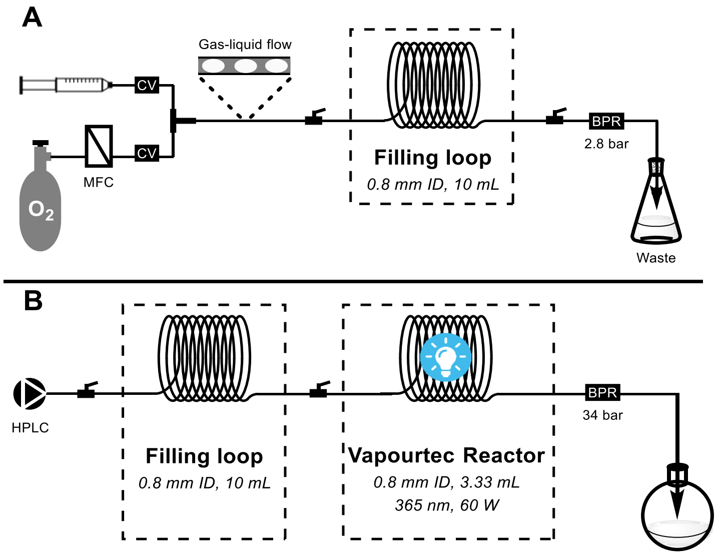

Figure S13: Overview of the procedure using 10 equivalents of O_2(g) in continuous-flow. (A) Loop filling of gas-liquid mixture, (B) photochemical reaction of gas-liquid mixture in a Vapourtec reactor at 34 bar.

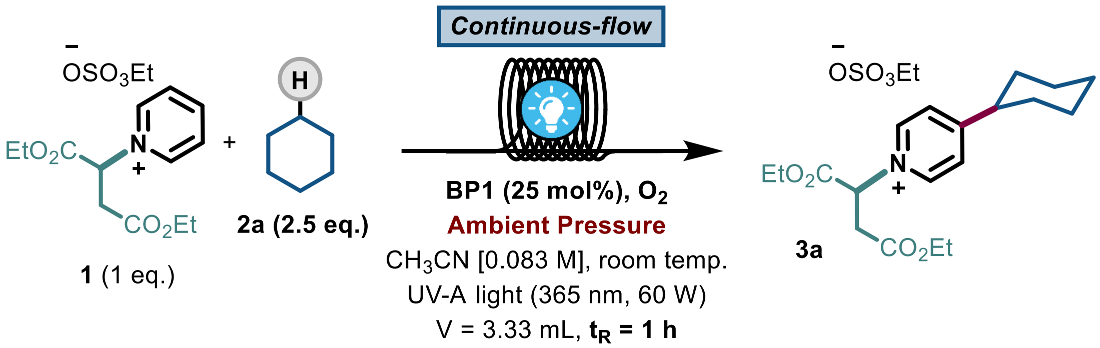

A stock solution containing **1** (94.4 mg, 0.25 mmol), **BP1** (11.4 mg, 25 mol%) and **2a** (68 μL, 0.625 mmol, 2.5 equiv.) in 3 mL of CH_3CN was prepared in a Pyrex glass vial, after which the vial was sealed with a rubber septum. The solution was sparged with O_2 and a O_2 filled balloon was inserted above the solution. The oxygen enriched solution was charged in a 5 mL syringe and was injected into a Vapourtec reactor with a flow rate of 0.055 mL·min^{-1} to achieve 1 h residence time. The outflow was collected, all solvent was removed through rotary evaporation, and the crude mixture was analyzed via ^{1}H NMR to determine the reaction yield (with 1,3,5-trimethoxybenzene as external standard).

Table S4. Yields for the formation of **3a** under oxygen-enriched flow conditions and catalytic amounts of **BP1** (25 mol%).

Entry	Pressure (bar)	Residence time (minutes)	Yield[1] (%)
1	32	10	traces
2	32	60	traces
3	Ambient	60	23

[1] Yield determined by quantitative ^{1}H NMR using 1,3,5-trimethoxybenzene as an internal standard.

Performing the Minisci reaction in flow with 10 eq. of O_2 under 34 bar pressure only gave trace amounts of product, after 10 and 60 minutes residence time (Table S4, entries 1 and 2). Oxygen has been shown to exhibit good quenching ability of the triplet excited state of benzophenone.[9] We reasoned that this interaction could explain the low conversion of pyridine under high O_2 concentrations, by effectively preventing the initial HAT event and therefore the overall reaction. So, we repeated the flow experiment at atmospheric pressure with an oxygen enriched solution (oxygen solubility in CH_3CN under atmospheric pressure is 2.4 mM), which was prepared by sparging the stock solution with O_2 before charging in a syringe. Under these conditions, a yield of 23 was reached after 1 h residence time (Table S4), confirming that the concentration of O_2 has an important effect on reaction efficiency. This result demonstrates that benzophenone can also be used in catalytic amounts by working under the appropriate concentration of oxygen in the system.

7.7 Oxygen Effect in Batch

After the preliminary results observed in section 7.5 using oxygen, we wondered if we could promote a catalytic transformation to full conversion under longer reaction times. For practical reasons, namely very low flow rates required for long residence times in flow, we decided to switch to batch conditions.

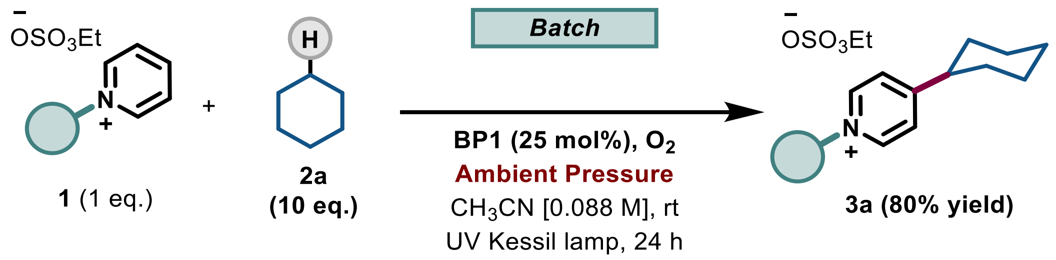

A stock solution containing **1** (188.7 mg, 0.5 mmol), **BP1** (22.8 mg, 25 mol%) and **2a** (540 μL, 5 mmol, 10 equiv.) in 6 mL of CH_3CN was prepared in a Pyrex glass vial, after which the vial was sealed with a rubber septum. The solution was sparged with O_2 and a O_2 filled balloon was inserted above the solution using a needle. The vial was then placed inside a home-made photochemical batch reactor (Figure S6) and it was irradiated for 24 h. Then, the solvent was removed through rotary evaporation and the crude mixture was analyzed via ^{1}H NMR using 1,3,5-trimethoxybenzene as external standard. We observed that after 24 h of irradiation, **3a** was formed in 65% yield. This result confirms that, given the proper reaction times and oxygen concentrations, benzophenone can be used in catalytic amounts. Nevertheless, this process is incompatible with fast and scalable reaction conditions, so given the cheap nature of benzophenone, working under stoichiometric conditions is more suitable in terms of productivity and scalability.

8 COMPUTATIONAL MECHANISTIC STUDIES

Density Functional Theory (DFT) calculations were run with the Gaussian 16 program package.[10] Geometry optimizations were performed with the ωB97X-D functional[11] and 6-31+G(d,p) as the basis set.[12] All the stationary poins were fully characterized via analytical frequency calculations as either minima (all positive eigenvalues) or transition states (one negative eigenvalue). Intrinsic reaction coordinate (IRC) calculations followed by geometry optimizations were used to confirm the minima linked between each transition state. Solvent effects were modeled using the conductor-like polarizable continuum model (CPCM)[13] for acetonitrile (CH_3CN) and dispersion effects (D2) were already included in the functional used. Although this computation method has demonstrated to give reliable results in similar chemical systems,[14] we are also aware of the likely systematic error arisen from using DFT methods to calculate the stability of delocalized radicals. Therefore, the computational results herein described should be analyzed from a qualitative point of view.

The final energies reported are refering to free energies (298.15 K, 1 atm), and the 0.0 point is based on the triplet state excited benzophenone (**BP1***).

In addition to the reaction profile described in the main text, a second possibility was also discovered and explored. In this alternative scenario, during the radical addition step, the cyclohexyl radical (**Cy2**) could also attack the activated pyridine ring (**Py1**) from *above*. A transition state for this process was also located (**TS2a**), although in a way higher energetic position in regard to the originally described **TS2b**. From this transition state, we could find another reaction intermediate **Py2a**, which could also be oxidized via the **TS3a** to give rise the same final product. Overall this pathway was discarded due to higher energetic barriers

All the raw data from the computational studies is available at the ioChem-BD repository[15] and can be accessed through https://doi.org/10.19061/iochem-bd-6-156.

8.1 Overall Free Energy Profiles

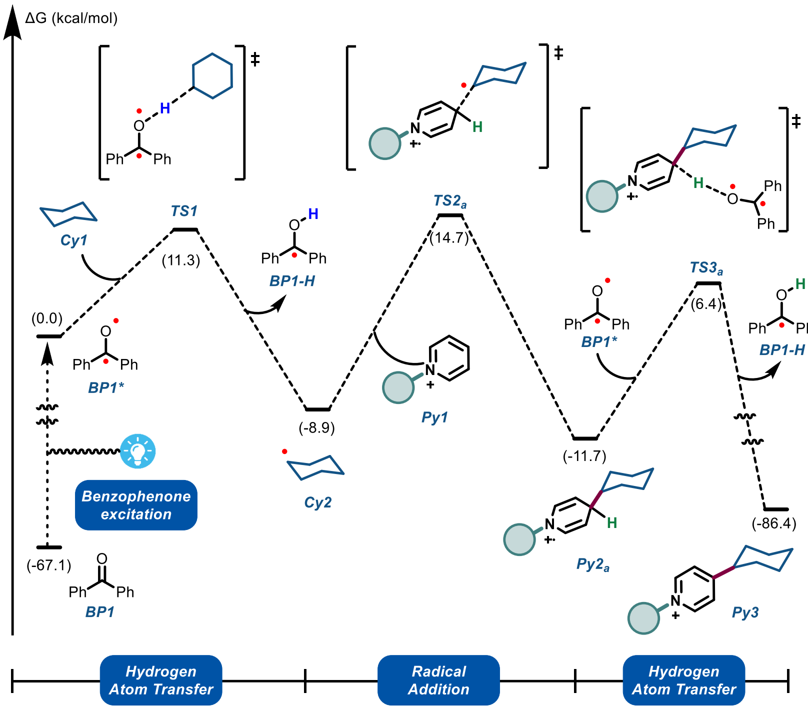

Figure S14. Alternative DFT profile for the radical addition towards **Py1** from *above*.

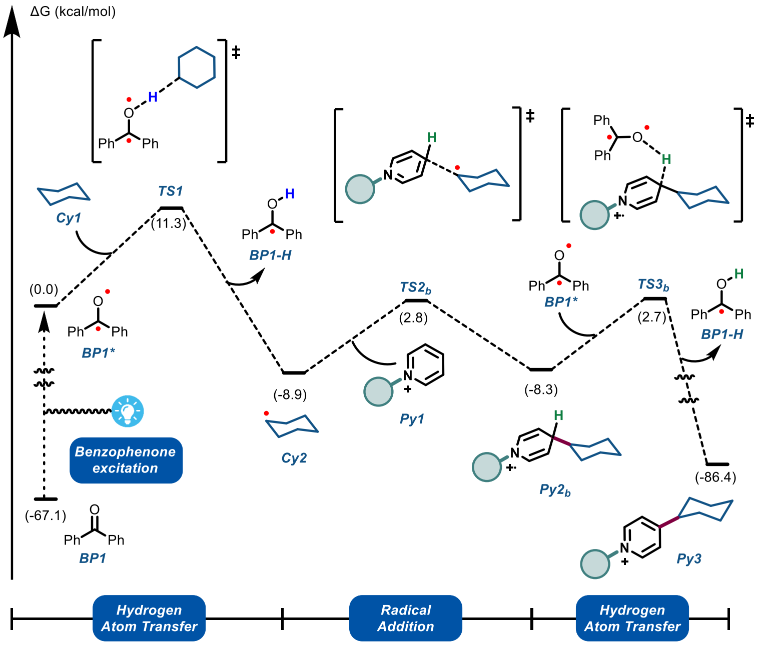

Figure S15. Main DFT profile for the radical addition towards **Py1** from *below*.

8.2 Cartesian Coordinates and Absolute Energies

The Gibbs free energies (in Hartree), and Cartesian coordinates (in Å) for all the optimized geometries at the ωB97X-D/6-31+G(d,p)/CPCM(acetonitrile) level of theory are presented below:

BP1

Energy (FREE) = -576.319323 Eh

	Atom	X	Y	Z
1	O	0.7119	2.9355	1.4848
2	C	0.4110	1.7469	1.4761
3	C	-1.0244	1.3370	1.3942
4	C	-1.5006	0.1849	2.0311
5	C	-2.8564	-0.1305	1.9827
6	C	-3.7393	0.6901	1.2826
7	C	-3.2694	1.8384	0.6429
8	C	-1.9200	2.1668	0.7087
9	C	1.4783	0.7023	1.5473
10	C	2.6584	0.9966	2.2411
11	C	3.6888	0.0649	2.2976
12	C	3.5580	-1.1598	1.6405
13	C	2.3936	-1.4517	0.9322
14	C	1.3513	-0.5287	0.8929
15	H	-0.8193	-0.4552	2.5822
16	H	-3.2219	-1.0172	2.4899
17	H	-4.7938	0.4370	1.2367
18	H	-3.9559	2.4770	0.0966
19	H	-1.5461	3.0625	0.2234
20	H	2.7540	1.9555	2.7400
21	H	4.5946	0.2923	2.8502
22	H	4.3650	-1.8847	1.6792
23	H	2.2949	-2.3984	0.4115
24	H	0.4493	-0.7599	0.3354

BP1_star

Energy (FREE) = -576.212469 Eh

	Atom	X	Y	Z
1	O	-0.0000	2.2537	0.0000
2	C	-0.0000	0.9238	0.0000
3	C	1.3073	0.3027	-0.0240
4	C	1.4999	-0.9858	-0.5708
5	C	2.7654	-1.5538	-0.5955
6	C	3.8725	-0.8506	-0.1102
7	C	3.6988	0.4358	0.4027
8	C	2.4353	1.0120	0.4488
9	C	-1.3073	0.3027	0.0240
10	C	-2.4353	1.0120	-0.4487
11	C	-3.6988	0.4357	-0.4027
12	C	-3.8725	-0.8506	0.1102
13	C	-2.7654	-1.5539	0.5955
14	C	-1.4999	-0.9858	0.5708
15	H	0.6593	-1.5280	-0.9901
16	H	2.8940	-2.5482	-1.0114
17	H	4.8598	-1.2997	-0.1386
18	H	4.5523	0.9908	0.7792
19	H	2.3069	2.0018	0.8745
20	H	-2.3069	2.0018	-0.8745
21	H	-4.5523	0.9908	-0.7792
22	H	-4.8598	-1.2997	0.1386
23	H	-2.8940	-2.5482	1.0114
24	H	-0.6593	-1.5281	0.9901

BP1-H

Energy (FREE) = -576.879958 Eh

	Atom	X	Y	Z
1	O	0.5312	2.9282	1.4293
2	C	0.2673	1.5893	1.4659
3	C	-1.1312	1.2538	1.4243
4	C	-1.6250	0.0116	1.8895
5	C	-2.9828	-0.2747	1.8511
6	C	-3.8954	0.6636	1.3615
7	C	-3.4278	1.9035	0.9194
8	C	-2.0717	2.2006	0.9520
9	C	1.3840	0.6720	1.5895
10	C	2.5766	1.0828	2.2251
11	C	3.6705	0.2288	2.3133
12	C	3.6106	-1.0552	1.7713
13	C	2.4420	-1.4737	1.1303
14	C	1.3464	-0.6261	1.0343
15	H	-0.9422	-0.7187	2.3096
16	H	-3.3348	-1.2336	2.2190
17	H	-4.9558	0.4347	1.3339
18	H	-4.1271	2.6439	0.5426
19	H	-1.7196	3.1640	0.6010
20	H	2.6362	2.0636	2.6879
21	H	4.5707	0.5650	2.8183
22	H	4.4648	-1.7205	1.8425
23	H	2.3900	-2.4638	0.6881
24	H	0.4637	-0.9558	0.4970
25	H	1.4408	3.0824	1.1490

Cy1

Energy (FREE) = -235.689705 Eh

	Atom	X	Y	Z
1	C	-4.0309	-2.3137	2.2876
2	C	-4.5127	-0.9294	1.8402
3	C	-2.5295	-2.3093	2.5949
4	H	-4.2313	-3.0396	1.4867
5	H	-4.5967	-2.6466	3.1653
6	C	-1.7174	-1.7949	1.4013
7	H	-2.3427	-1.6605	3.4625
8	H	-2.1968	-3.3154	2.8746
9	C	-3.7006	-0.4151	0.6467
10	H	-4.4065	-0.2246	2.6771
11	H	-5.5787	-0.9623	1.5875
12	C	-2.1992	-0.4107	0.9539
13	H	-1.8236	-2.4998	0.5644
14	H	-0.6514	-1.7620	1.6540
15	H	-1.6335	-0.0777	0.0762
16	H	-1.9989	0.3152	1.7548
17	H	-3.8875	-1.0638	-0.2209
18	H	-4.0333	0.5911	0.3669

Cy2

Energy (FREE) = -235.036347 Eh

	Atom	X	Y	Z
1	C	-4.0381	-2.3586	2.2659
2	C	-4.5239	-1.0786	1.6653
3	C	-2.5361	-2.3056	2.5950
4	H	-4.1985	-3.1838	1.5464
5	H	-4.6195	-2.6167	3.1575
6	C	-1.7241	-1.7935	1.4018
7	H	-2.3783	-1.6346	3.4498
8	H	-2.1865	-3.2987	2.8982
9	C	-3.7024	-0.4292	0.5983
10	H	-5.5597	-0.7793	1.8031
11	C	-2.2063	-0.4098	0.9565
12	H	-1.8247	-2.4994	0.5648
13	H	-0.6595	-1.7564	1.6590
14	H	-1.6228	-0.0589	0.0980
15	H	-2.0405	0.3066	1.7720
16	H	-3.8176	-0.9941	-0.3462
17	H	-4.0626	0.5842	0.3910

Py1

Energy (FREE) = -861.259996 Eh

	Atom	X	Y	Z
1	O	-0.7992	2.3312	-0.7185
2	C	-0.0727	1.5158	-0.1991
3	C	-0.5728	0.2874	0.5715
4	C	0.3029	-0.9561	0.3748
5	C	1.5479	-0.8484	1.2487
6	O	2.7313	-1.2321	0.7861
7	C	2.9623	-1.6738	-0.5723
8	C	2.5755	-3.1259	-0.7673
9	O	1.4637	-0.4499	2.3929
10	N	-1.9825	0.0290	0.2209
11	C	-2.3041	-0.2774	-1.0556
12	C	-3.6120	-0.5285	-1.4081
13	C	-4.6042	-0.4583	-0.4326
14	C	-4.2544	-0.1372	0.8753
15	C	-2.9302	0.1044	1.1791
16	O	1.2460	1.5469	-0.1799
17	C	1.9075	2.6712	-0.8200
18	C	3.3948	2.4394	-0.6969
19	H	-0.5484	0.5504	1.6322
20	H	0.5261	-1.1160	-0.6787
21	H	-0.2432	-1.8333	0.7360
22	H	4.0358	-1.5368	-0.7014
23	H	2.4557	-1.0008	-1.2686
24	H	3.1038	-3.7605	-0.0518
25	H	2.8546	-3.4367	-1.7776
26	H	1.5006	-3.2870	-0.6492
27	H	-1.4937	-0.3136	-1.7715
28	H	-3.8423	-0.7727	-2.4367
29	H	-5.6390	-0.6506	-0.6907
30	H	-4.9941	-0.0711	1.6623
31	H	-2.5967	0.3582	2.1764
32	H	1.5861	3.5830	-0.3118
33	H	1.5779	2.7074	-1.8604
34	H	3.6958	2.3795	0.3519
35	H	3.9251	3.2744	-1.1617
36	H	3.6909	1.5183	-1.2052

Py2a

Energy (FREE) = -1096.300877 Eh

	Atom	X	Y	Z
1	O	-1.4183	-2.4683	-0.6528
2	C	-2.1953	-1.7417	-0.0763
3	C	-1.7996	-0.4233	0.6035
4	C	-2.6516	0.7601	0.1344
5	C	-2.3932	1.9901	0.9890
6	O	-2.9026	3.1566	0.6091
7	C	-3.6048	3.3461	-0.6435
8	C	-2.6439	3.7335	-1.7498
9	O	-1.7649	1.9297	2.0269
10	N	-0.3637	-0.1843	0.4168
11	C	0.1391	0.0224	-0.8432
12	C	1.4630	0.2303	-1.0454
13	C	2.4584	0.1793	0.0530
14	C	1.7968	0.0499	1.3732
15	C	0.4597	-0.1439	1.5087
16	O	-3.4852	-1.9646	0.0893
17	C	-4.0248	-3.1915	-0.4714
18	C	-5.5077	-3.2021	-0.1852
19	H	-1.9613	-0.5593	1.6745
20	H	-3.7039	0.4798	0.2421
21	H	-2.4932	0.9677	-0.9257
22	H	-4.3027	4.1555	-0.4300
23	H	-4.1891	2.4571	-0.8888
24	H	-2.0893	4.6341	-1.4759
25	H	-3.2116	3.9394	-2.6612
26	H	-1.9270	2.9372	-1.9693
27	H	-0.5719	0.0111	-1.6579
28	H	1.7949	0.4131	-2.0598
29	H	3.0351	1.1191	0.0496
30	H	2.3905	0.1088	2.2768
31	H	-0.0205	-0.2513	2.4712
32	H	-3.5072	-4.0318	-0.0032
33	H	-3.8109	-3.1956	-1.5424
34	H	-5.6995	-3.1823	0.8904
35	H	-5.9422	-4.1170	-0.5961
36	H	-6.0023	-2.3462	-0.6513
37	H	4.9157	-1.6372	-1.6746
38	H	5.8755	0.5449	-2.2970
39	H	3.6771	-0.6103	-2.3582
40	C	4.3314	-0.7270	-1.4895
41	C	5.2921	0.4617	-1.3742
42	H	6.9033	-0.5357	-0.3483
43	C	6.2281	0.3123	-0.1702
44	C	3.5273	-0.9732	-0.2016
45	H	4.7274	1.3999	-1.2879
46	H	6.8569	1.2030	-0.0678
47	H	2.9484	-1.8967	-0.3099
48	H	5.1002	-2.0327	0.7649
49	C	4.5040	-1.1354	0.9742

50	C	5.4507	0.0592	1.1259
51	H	6.1446	-0.1285	1.9516
52	H	3.9751	-1.3401	1.9093
53	H	4.8859	0.9608	1.4003

Py2b

Energy (FREE) = -1096.295489 Eh

	Atom	X	Y	Z
1	O	-3.0752	-2.1001	-0.6399
2	C	-3.1340	-1.0148	-0.1077
3	C	-1.9325	-0.3038	0.5263
4	C	-1.7924	1.1623	0.1000
5	C	-0.7812	1.8836	0.9797
6	O	-0.2954	3.0548	0.5861
7	C	-0.5789	3.6409	-0.7077
8	C	0.4226	3.1731	-1.7450
9	O	-0.4318	1.4320	2.0522
10	N	-0.6952	-1.0438	0.2488
11	C	-0.2976	-1.2408	-1.0544
12	C	0.9184	-1.7628	-1.3377
13	C	1.9081	-2.0918	-0.2836
14	C	1.3139	-2.0030	1.0716
15	C	0.0829	-1.4689	1.2895
16	O	-4.2256	-0.2923	0.0552
17	C	-5.4720	-0.8523	-0.4387
18	C	-6.5628	0.1495	-0.1431
19	H	-2.0875	-0.3351	1.6068
20	H	-2.7617	1.6550	0.2237
21	H	-1.5369	1.2326	-0.9588
22	H	-0.4866	4.7123	-0.5313
23	H	-1.6120	3.4370	-0.9966
24	H	1.4427	3.3781	-1.4107
25	H	0.2486	3.7118	-2.6802
26	H	0.3330	2.1027	-1.9519
27	H	-1.0038	-0.9577	-1.8225
28	H	1.1945	-1.8983	-2.3771
29	H	1.8878	-2.3223	1.9331
30	H	-0.3301	-1.3342	2.2793
31	H	-5.6375	-1.8055	0.0684
32	H	-5.3581	-1.0356	-1.5094
33	H	-6.6501	0.3278	0.9315
34	H	-7.5155	-0.2453	-0.5055
35	H	-6.3693	1.0994	-0.6476
36	C	3.1840	-1.1667	-0.4324
37	C	4.3849	-1.7247	0.3587
38	H	4.0507	-2.2045	1.2848
39	H	4.8645	-2.5061	-0.2409
40	C	5.4023	-0.6192	0.7038
41	H	5.1308	-0.1492	1.6570
42	H	6.3920	-1.0635	0.8466
43	C	5.4390	0.4529	-0.3870
44	H	6.2421	1.1707	-0.1941
45	H	5.6749	-0.0256	-1.3460
46	C	4.0843	1.1866	-0.4818
47	H	4.0959	2.0791	0.1528
48	H	3.9256	1.5337	-1.5087
49	C	2.9068	0.2900	-0.0531

50	H	2.7555	0.3558	1.0314
51	H	1.9841	0.6476	-0.5217
52	H	3.4282	-1.1976	-1.5013
53	H	2.2881	-3.1127	-0.4501

Py3

Energy (FREE) = -1095.752481 Eh

	Atom	X	Y	Z
1	O	-1.2978	-2.4593	-0.2413
2	C	-1.9819	-1.5229	0.1020
3	C	-1.4252	-0.2218	0.6926
4	C	-2.1802	1.0312	0.2311
5	C	-3.4851	1.1634	1.0082
6	O	-4.6070	1.5115	0.3895
7	C	-4.7210	1.6754	-1.0434
8	C	-4.2407	3.0394	-1.4964
9	O	-3.5043	0.9935	2.2107
10	N	0.0171	-0.1315	0.4082
11	C	0.4463	-0.0337	-0.8689
12	C	1.7886	0.0561	-1.1547
13	C	2.7361	0.0374	-0.1242
14	C	2.2547	-0.0743	1.1871
15	C	0.9036	-0.1555	1.4278
16	O	-3.2995	-1.4633	0.0546
17	C	-4.0129	-2.6315	-0.4321
18	C	-5.4843	-2.2912	-0.4164
19	H	-1.5335	-0.2963	1.7776
20	H	-2.3186	1.0314	-0.8486
21	H	-1.5863	1.9131	0.4914
22	H	-5.7896	1.5603	-1.2250
23	H	-4.2078	0.8539	-1.5494
24	H	-4.7776	3.8286	-0.9646
25	H	-4.4362	3.1486	-2.5665
26	H	-3.1682	3.1761	-1.3330
27	H	-0.3090	-0.0298	-1.6439
28	H	2.0937	0.1380	-2.1908
29	H	2.9278	-0.0991	2.0352
30	H	0.4946	-0.2384	2.4261
31	H	-3.7735	-3.4693	0.2264
32	H	-3.6491	-2.8531	-1.4377
33	H	-5.8181	-2.0459	0.5948
34	H	-6.0536	-3.1551	-0.7685
35	H	-5.6979	-1.4467	-1.0763
36	C	4.2073	0.1301	-0.4151
37	C	4.9297	-1.2025	-0.0769
38	H	4.4730	-1.6607	0.8073
39	H	4.7770	-1.8999	-0.9072
40	C	6.4343	-0.9876	0.1759
41	H	6.6071	-0.7856	1.2400
42	H	6.9783	-1.9076	-0.0586
43	C	6.9705	0.1857	-0.6456
44	H	8.0577	0.2593	-0.5461
45	H	6.7691	-0.0019	-1.7078
46	C	6.3093	1.5088	-0.2065
47	H	6.9100	1.9839	0.5762
48	H	6.2850	2.2050	-1.0516
49	C	4.8795	1.2982	0.3286

50	H	4.9080	1.0725	1.4013
51	H	4.2920	2.2144	0.2144
52	H	4.3122	0.3187	-1.4893

TS1

Energy (FREE) = -811.884175 Eh

	Atom	X	Y	Z
1	O	0.0637	-1.0923	1.1334
2	C	0.9311	-0.3104	0.4634
3	C	2.1384	-0.9838	0.0550
4	C	3.3423	-0.2817	-0.2018
5	C	4.4900	-0.9599	-0.5837
6	C	4.4909	-2.3542	-0.7014
7	C	3.3196	-3.0636	-0.4268
8	C	2.1597	-2.3967	-0.0540
9	C	0.6524	1.1107	0.3186
10	C	-0.0587	1.8051	1.3183
11	C	-0.3667	3.1512	1.1663
12	C	0.0242	3.8410	0.0168
13	C	0.7314	3.1662	-0.9802
14	C	1.0414	1.8189	-0.8358
15	H	3.3743	0.7958	-0.0820
16	H	5.3993	-0.3992	-0.7782
17	H	5.3938	-2.8784	-0.9975
18	H	3.3081	-4.1460	-0.5131
19	H	1.2501	-2.9557	0.1366
20	H	-0.3622	1.2809	2.2186
21	H	-0.9117	3.6670	1.9509
22	H	-0.2219	4.8912	-0.1019
23	H	1.0299	3.6890	-1.8837
24	H	1.5590	1.2987	-1.6355
25	C	-3.1711	-1.6788	0.8322
26	C	-4.6362	-1.4972	0.3949
27	C	-2.4226	-0.3657	0.6902
28	H	-2.6987	-2.4365	0.1933
29	H	-3.1230	-2.0419	1.8636
30	C	-2.4785	0.2104	-0.7127
31	H	-2.7260	0.3684	1.4471
32	H	-1.2983	-0.5953	0.9578
33	C	-4.7229	-0.9279	-1.0237
34	H	-5.1380	-0.8138	1.0929
35	H	-5.1580	-2.4582	0.4571
36	C	-3.9450	0.3851	-1.1473
37	H	-3.9783	0.7575	-2.1768
38	H	-4.4176	1.1487	-0.5150
39	H	-4.3114	-1.6598	-1.7323
40	H	-5.7710	-0.7708	-1.3016
41	H	-1.9758	-0.4750	-1.4081
42	H	-1.9517	1.1684	-0.7586

TS2a

Energy (FREE) = -1096.258814 Eh

	Atom	X	Y	Z
1	O	-0.7138	2.3131	0.5366
2	C	-1.6532	1.7917	-0.0163
3	C	-1.6295	0.3834	-0.6225
4	C	-2.7698	-0.5029	-0.1198
5	C	-2.8666	-1.7691	-0.9573
6	O	-3.6600	-2.7551	-0.5486
7	C	-4.3546	-2.7290	0.7168
8	C	-3.4992	-3.3237	1.8184
9	O	-2.2688	-1.8904	-2.0050
10	N	-0.3115	-0.2249	-0.3970
11	C	0.1303	-0.4245	0.8748
12	C	1.3700	-0.9450	1.1137
13	C	2.2396	-1.1988	0.0290
14	C	1.7036	-1.1143	-1.2746
15	C	0.4514	-0.5965	-1.4584
16	O	-2.8370	2.3445	-0.2151
17	C	-3.0176	3.6938	0.2828
18	C	-4.4420	4.0961	-0.0186
19	H	-1.7444	0.5001	-1.7016
20	H	-3.7059	0.0546	-0.2199
21	H	-2.6556	-0.7291	0.9425
22	H	-5.2428	-3.3371	0.5439
23	H	-4.6881	-1.7156	0.9502
24	H	-3.1942	-4.3391	1.5560
25	H	-4.0781	-3.3631	2.7445
26	H	-2.5995	-2.7307	2.0053
27	H	-0.5458	-0.1528	1.6733
28	H	1.6840	-1.1105	2.1356
29	H	3.1649	-1.7359	0.1957
30	H	2.2786	-1.4201	-2.1386
31	H	0.0005	-0.4817	-2.4339
32	H	-2.2913	4.3407	-0.2146
33	H	-2.8063	3.6934	1.3546
34	H	-4.6334	4.0774	-1.0940
35	H	-4.6125	5.1122	0.3449
36	H	-5.1489	3.4281	0.4790
37	H	4.6166	1.7360	1.5845
38	H	5.9735	-0.1388	2.3160
39	H	3.5248	0.5293	2.2292
40	C	4.2170	0.7237	1.4037
41	C	5.3835	-0.2724	1.4045
42	H	6.7403	0.8868	0.1913
43	C	6.2665	-0.1037	0.1653
44	C	3.4874	0.7816	0.0976
45	H	4.9945	-1.2982	1.4369
46	H	7.0740	-0.8423	0.1772
47	H	2.5711	1.3689	0.0798
48	H	4.7210	1.7890	-1.2677
49	C	4.2994	0.7754	-1.1590

50	C	5.4508	-0.2362	-1.1236
51	H	6.0908	-0.0914	-1.9989
52	H	3.6592	0.6271	-2.0344
53	H	5.0484	-1.2550	-1.1972

TS2b

Energy (FREE) = -1096.277813 Eh

	Atom	X	Y	Z
1	O	3.1072	-1.9950	0.6480
2	C	3.0177	-0.9716	0.0079
3	C	1.6967	-0.3872	-0.5049
4	C	1.5068	1.0842	-0.1253
5	C	0.3284	1.6879	-0.8719
6	O	-0.1537	2.8620	-0.4808
7	C	0.3395	3.5790	0.6770
8	C	-0.4278	3.1903	1.9246
9	O	-0.1720	1.1383	-1.8336
10	N	0.5649	-1.2073	-0.0544
11	C	0.3061	-1.3426	1.2778
12	C	-0.7870	-2.0344	1.7165
13	C	-1.7056	-2.5672	0.7803
14	C	-1.3432	-2.5182	-0.5854
15	C	-0.2383	-1.8099	-0.9720
16	O	4.0282	-0.2154	-0.3807
17	C	5.3635	-0.6508	-0.0122
18	C	6.3345	0.3760	-0.5455
19	H	1.7264	-0.4590	-1.5940
20	H	2.4094	1.6363	-0.4047
21	H	1.3948	1.1945	0.9551
22	H	0.1703	4.6256	0.4247
23	H	1.4158	3.4303	0.7839
24	H	-1.4990	3.3516	1.7811
25	H	-0.0931	3.8123	2.7591
26	H	-0.2672	2.1437	2.1981
27	H	1.0140	-0.8917	1.9602
28	H	-0.9568	-2.1281	2.7813
29	H	-1.9465	-3.0010	-1.3434
30	H	0.0494	-1.6988	-2.0085
31	H	5.5280	-1.6394	-0.4467
32	H	5.4045	-0.7313	1.0763
33	H	6.2664	0.4515	-1.6336
34	H	7.3517	0.0732	-0.2840
35	H	6.1443	1.3591	-0.1077
36	C	-3.3888	-0.9894	0.9126
37	C	-4.3627	-1.3475	-0.1729
38	H	-4.3480	-2.4244	-0.3831
39	H	-5.3766	-1.1454	0.2075
40	C	-4.1122	-0.5264	-1.4390
41	H	-3.1135	-0.7625	-1.8242
42	H	-4.8205	-0.8147	-2.2209
43	C	-4.2117	0.9863	-1.1628
44	H	-3.6666	1.5237	-1.9454
45	H	-5.2573	1.3033	-1.2361
46	C	-3.6599	1.3778	0.2268
47	H	-3.1523	2.3457	0.1770
48	H	-4.4808	1.4897	0.9437
49	C	-2.6789	0.3208	0.7694

50	H	-1.8581	0.2270	0.0435
51	H	-2.2412	0.6370	1.7188
52	H	-3.6315	-1.3256	1.9184
53	H	-2.4698	-3.2608	1.1089

TS3a

Energy (FREE) = -1672.484546 Eh

	Atom	X	Y	Z
1	O	4.7601	-1.8111	-0.3035
2	C	4.6131	-0.6112	-0.3754
3	C	3.2920	0.0759	-0.7381
4	C	2.9524	1.2576	0.1771
5	C	1.8470	2.1070	-0.4304
6	O	1.1738	2.9531	0.3475
7	C	1.3944	3.0633	1.7718
8	C	0.5141	2.0958	2.5370
9	O	1.5879	2.0745	-1.6162
10	N	2.1973	-0.8846	-0.7834
11	C	1.8425	-1.5977	0.3450
12	C	0.7222	-2.3583	0.3653
13	C	-0.2211	-2.3711	-0.7475
14	C	0.3651	-1.8374	-1.9684
15	C	1.4946	-1.0844	-1.9482
16	O	5.5706	0.2857	-0.2013
17	C	6.9003	-0.2134	0.0912
18	C	7.8062	0.9849	0.2517
19	H	3.4229	0.4625	-1.7522
20	H	3.8363	1.8933	0.2896
21	H	2.6912	0.9002	1.1748
22	H	1.1267	4.0953	1.9988
23	H	2.4539	2.9348	2.0024
24	H	-0.5387	2.2789	2.3070
25	H	0.6605	2.2385	3.6107
26	H	0.7493	1.0546	2.2983
27	H	2.5196	-1.5384	1.1869
28	H	0.5189	-2.9358	1.2607
29	H	-0.8942	-1.1904	-0.4584
30	H	-0.1481	-1.9362	-2.9164
31	H	1.8877	-0.5970	-2.8303
32	H	7.2112	-0.8572	-0.7350
33	H	6.8450	-0.8127	1.0030
34	H	7.8387	1.5772	-0.6663
35	H	8.8190	0.6394	0.4748
36	H	7.4706	1.6219	1.0740
37	H	-2.5302	-4.7216	0.3817
38	H	-4.0100	-2.9073	1.1144
39	H	-1.6162	-3.5557	1.3175
40	C	-2.1416	-3.6955	0.3659
41	C	-3.3234	-2.7252	0.2804
42	H	-4.5576	-3.7832	-1.1412
43	C	-4.0590	-2.8076	-1.0580
44	C	-1.1680	-3.5890	-0.8254
45	H	-2.9654	-1.6982	0.4048
46	H	-4.8427	-2.0435	-1.1014
47	H	-0.5071	-4.4659	-0.7818
48	H	-2.4244	-4.6880	-2.1600
49	C	-1.9680	-3.6900	-2.1342

50	C	-3.0830	-2.6454	-2.2266
51	H	-3.6107	-2.7465	-3.1808
52	H	-1.3082	-3.6441	-3.0056
53	H	-2.6548	-1.6334	-2.2078
54	H	-3.4779	-0.9901	5.0591
55	H	-1.3544	-1.8178	4.0135
56	C	-3.1526	-0.6488	4.0847
57	C	-1.9858	-1.1004	3.5015
58	C	-1.5994	-0.6234	2.2560
59	C	-3.9723	0.3300	3.3578
60	H	-0.6706	-0.9502	1.8077
61	H	-4.9032	0.6571	3.8087
62	C	-3.6120	0.8201	2.1271
63	C	-2.4276	0.3620	1.5030
64	H	-2.6269	3.2090	1.2836
65	C	-2.0395	0.7453	0.1816
66	H	-4.2719	1.5070	1.6125
67	O	-1.1965	0.0407	-0.4993
68	C	-2.7897	3.1544	0.2120
69	C	-2.5410	1.9623	-0.4849
70	H	-3.3900	5.2104	0.0799
71	C	-3.2112	4.2919	-0.4692
72	C	-2.7030	1.9412	-1.8791
73	C	-3.3911	4.2546	-1.8514
74	H	-2.4939	1.0253	-2.4216
75	C	-3.1348	3.0763	-2.5543
76	H	-3.7229	5.1423	-2.3802
77	H	-3.2700	3.0439	-3.6305

TS3b

Energy (FREE) = -1672.490300 Eh

	Atom	X	Y	Z
1	O	2.1386	-0.8289	0.3732
2	C	2.7292	0.0591	-0.2009
3	C	2.0885	1.3709	-0.6701
4	C	2.6207	2.5612	0.1370
5	C	2.2022	3.8767	-0.4987
6	O	2.3662	5.0056	0.1868
7	C	2.8367	5.0422	1.5551
8	C	1.6782	4.9671	2.5296
9	O	1.7579	3.9363	-1.6273
10	N	0.6362	1.2615	-0.6367
11	C	-0.0328	1.0890	0.5568
12	C	-1.3583	0.8092	0.5791
13	C	-2.0976	0.5463	-0.6466
14	C	-1.4096	1.0344	-1.8357
15	C	-0.0830	1.3131	-1.8129
16	O	4.0157	0.0394	-0.5088
17	C	4.7654	-1.1382	-0.1126
18	C	6.1781	-0.9683	-0.6189
19	H	2.3769	1.5153	-1.7125
20	H	3.7151	2.5203	0.1408
21	H	2.3042	2.5033	1.1804
22	H	3.3474	6.0024	1.6293
23	H	3.5767	4.2573	1.7230
24	H	0.9702	5.7783	2.3443
25	H	2.0601	5.0660	3.5493
26	H	1.1421	4.0165	2.4584
27	H	0.5538	1.1699	1.4613
28	H	-1.8440	0.6817	1.5412
29	H	-1.9284	1.1003	-2.7855
30	H	0.4732	1.6045	-2.6938
31	H	4.2751	-2.0130	-0.5466
32	H	4.7234	-1.2162	0.9765
33	H	6.1953	-0.8825	-1.7083
34	H	6.7666	-1.8439	-0.3328
35	H	6.6466	-0.0814	-0.1848
36	C	-3.6212	0.6026	-0.5776
37	C	-4.3036	-0.2520	-1.6693
38	H	-3.7041	-0.2489	-2.5869
39	H	-4.3369	-1.2942	-1.3307
40	C	-5.7275	0.2383	-1.9916
41	H	-5.6893	1.0085	-2.7724
42	H	-6.3209	-0.5866	-2.3983
43	C	-6.3934	0.8273	-0.7471
44	H	-7.4450	1.0596	-0.9433
45	H	-6.3859	0.0714	0.0488
46	C	-5.6486	2.0948	-0.2784
47	H	-6.0926	2.9805	-0.7462
48	H	-5.7761	2.2142	0.8033
49	C	-4.1465	2.0459	-0.6197

50	H	-3.9691	2.4539	-1.6234
51	H	-3.5782	2.6735	0.0754
52	H	-3.8948	0.1853	0.4009
53	H	-1.7925	-0.7462	-0.8206
54	H	2.7609	-3.6219	-3.0114
55	C	2.3213	-3.7311	-2.0262
56	H	0.6220	-2.4890	-2.4647
57	H	3.8878	-5.0300	-1.2741
58	C	1.1397	-3.0945	-1.7322
59	C	2.9546	-4.5224	-1.0632
60	O	-1.2215	-1.8750	-1.1092
61	C	0.5344	-3.1976	-0.3749
62	C	2.3250	-4.6956	0.2477
63	C	-0.6426	-2.4448	-0.0993
64	C	1.1644	-4.0689	0.5737
65	H	2.8040	-5.3524	0.9659
66	C	-1.2202	-2.2696	1.2465
67	H	0.7108	-4.2312	1.5433
68	H	-3.2380	-2.4719	0.5188
69	C	-2.6158	-2.2643	1.3840
70	H	0.6578	-1.9354	2.2555
71	C	-0.4203	-1.9919	2.3643
72	C	-3.2010	-1.9937	2.6157
73	C	-1.0095	-1.7166	3.5937
74	H	-4.2819	-1.9946	2.7108
75	C	-2.3988	-1.7170	3.7232
76	H	-0.3835	-1.4843	4.4490
77	H	-2.8548	-1.4974	4.6831

9 NMR DATA

^{1}H NMR (400 MHz, $CDCl_3$) – **4a**

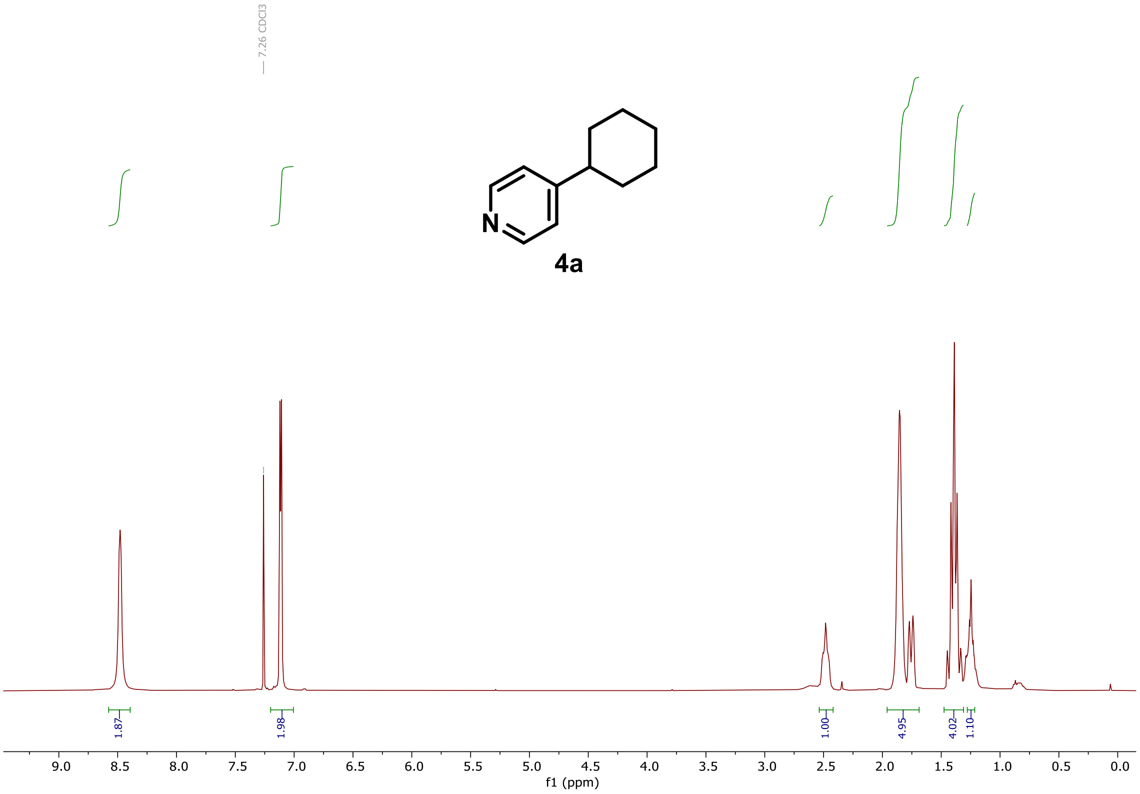

^{13}C NMR (101 MHz, $CDCl_3$) – **4a**

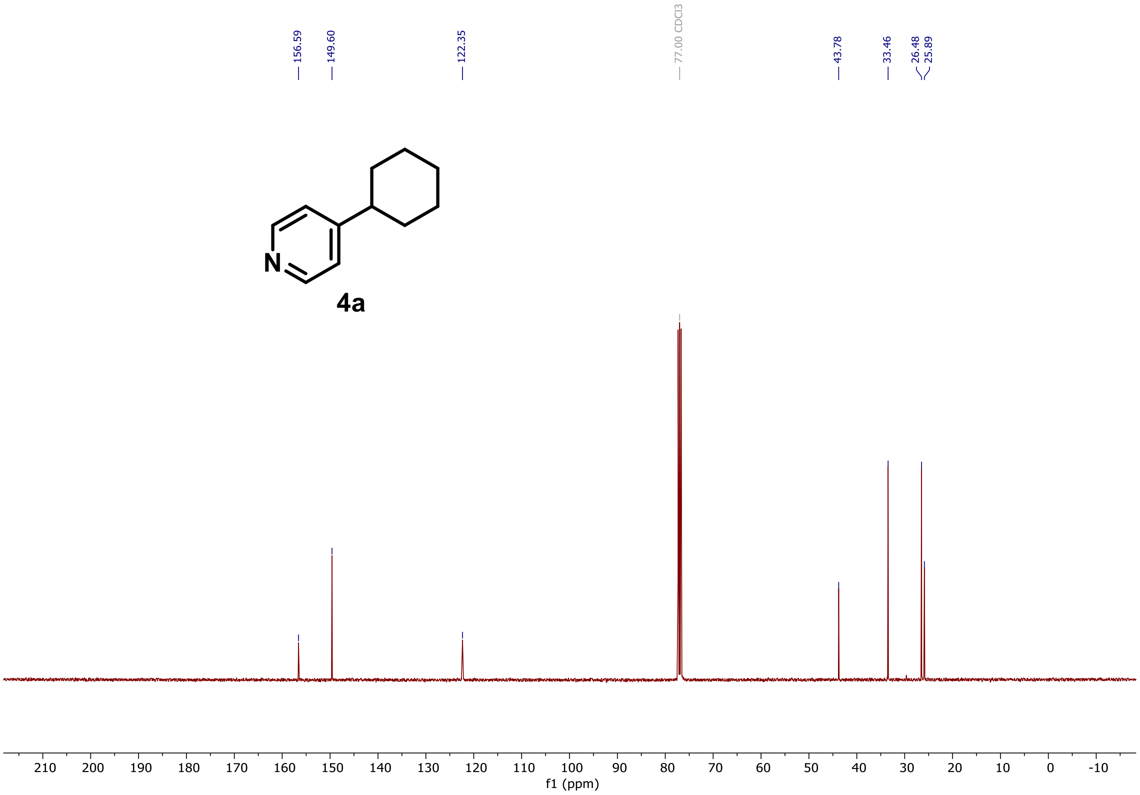

^{1}H NMR (400 MHz, $CDCl_3$) – **4b**

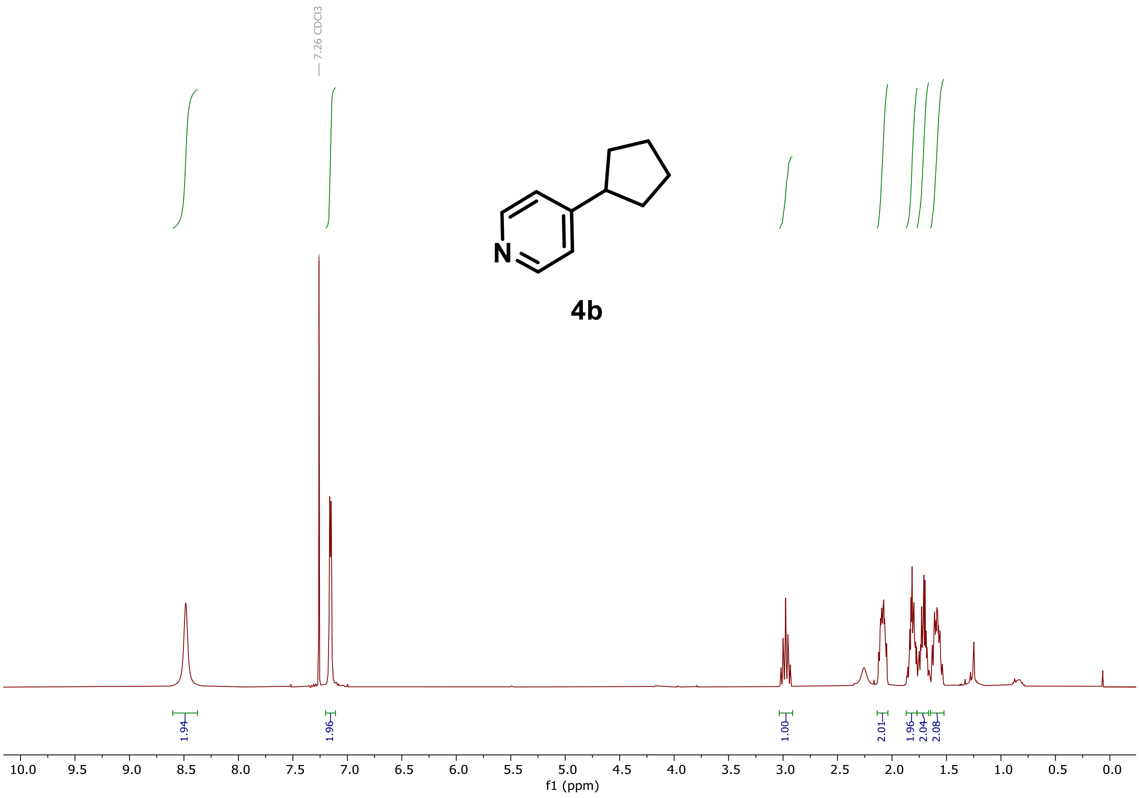

^{13}C NMR (101 MHz, $CDCl_3$) – **4b**

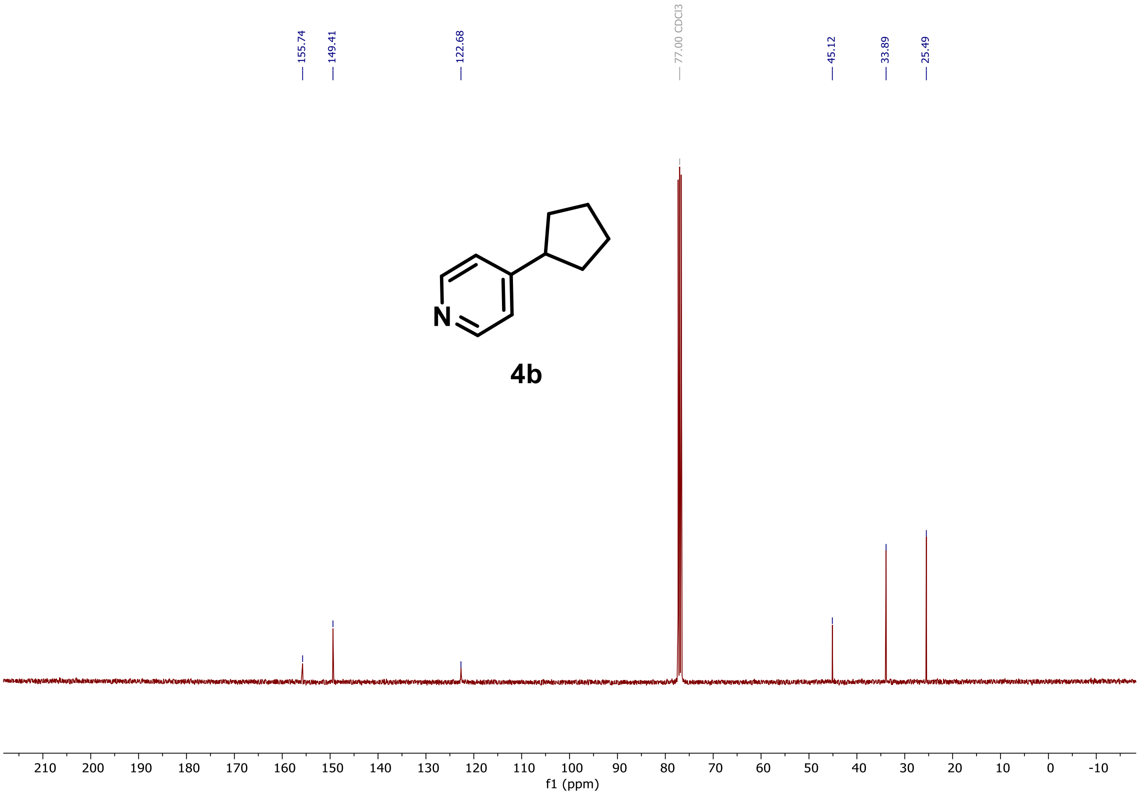

^{1}H NMR (400 MHz, $CDCl_3$) – **4c**

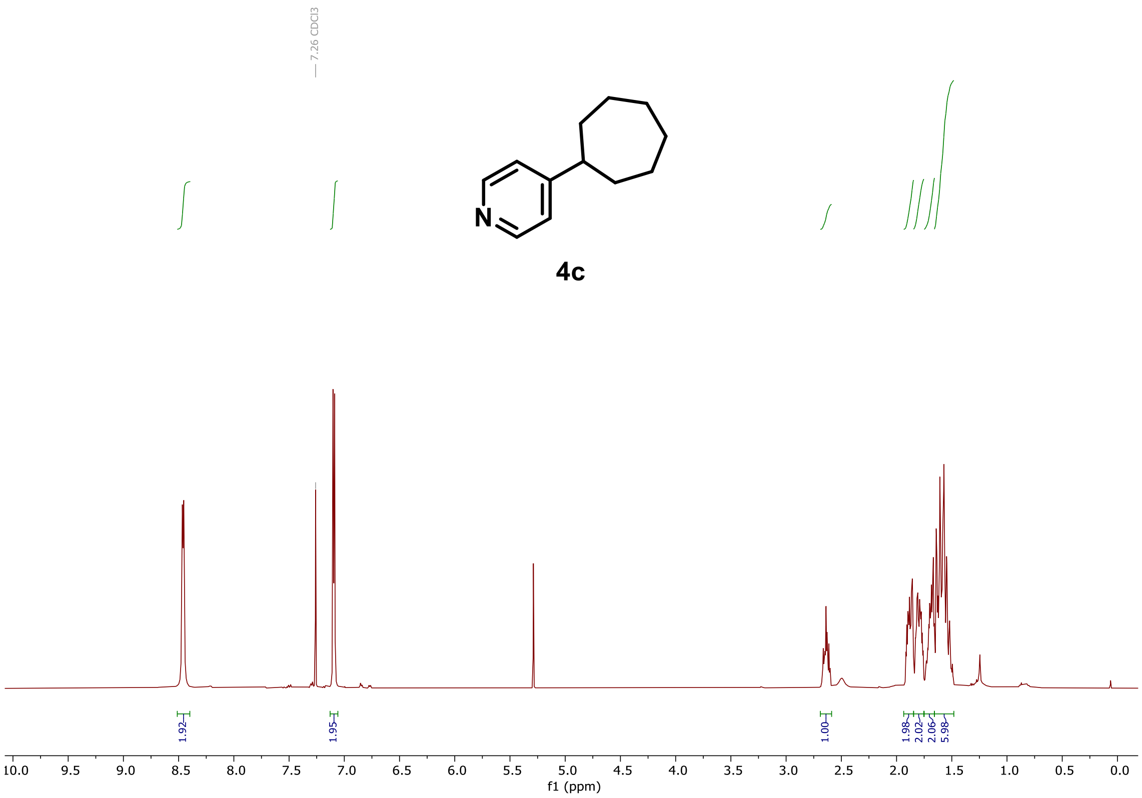

^{13}C NMR (101 MHz, $CDCl_3$) – **4c**

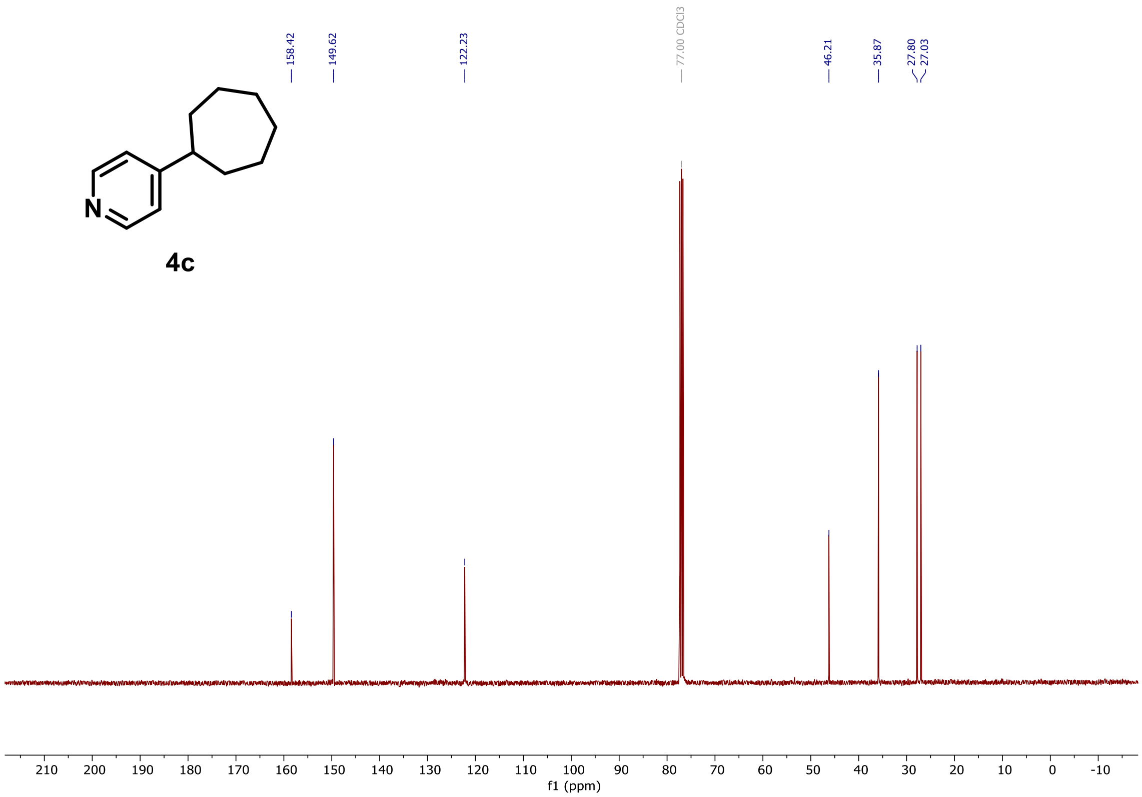

^1H NMR (400 MHz, $CDCl_3$) – **4d**

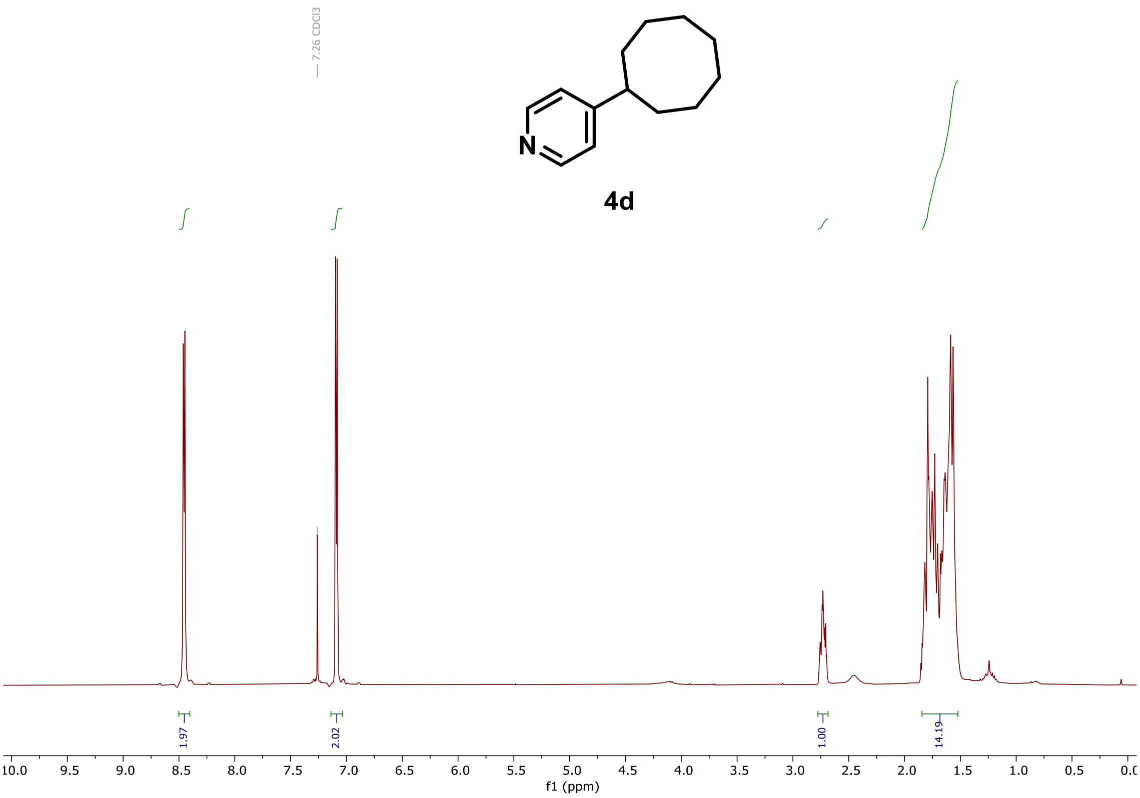

^{13}C NMR (101 MHz, $CDCl_3$) – **4d**

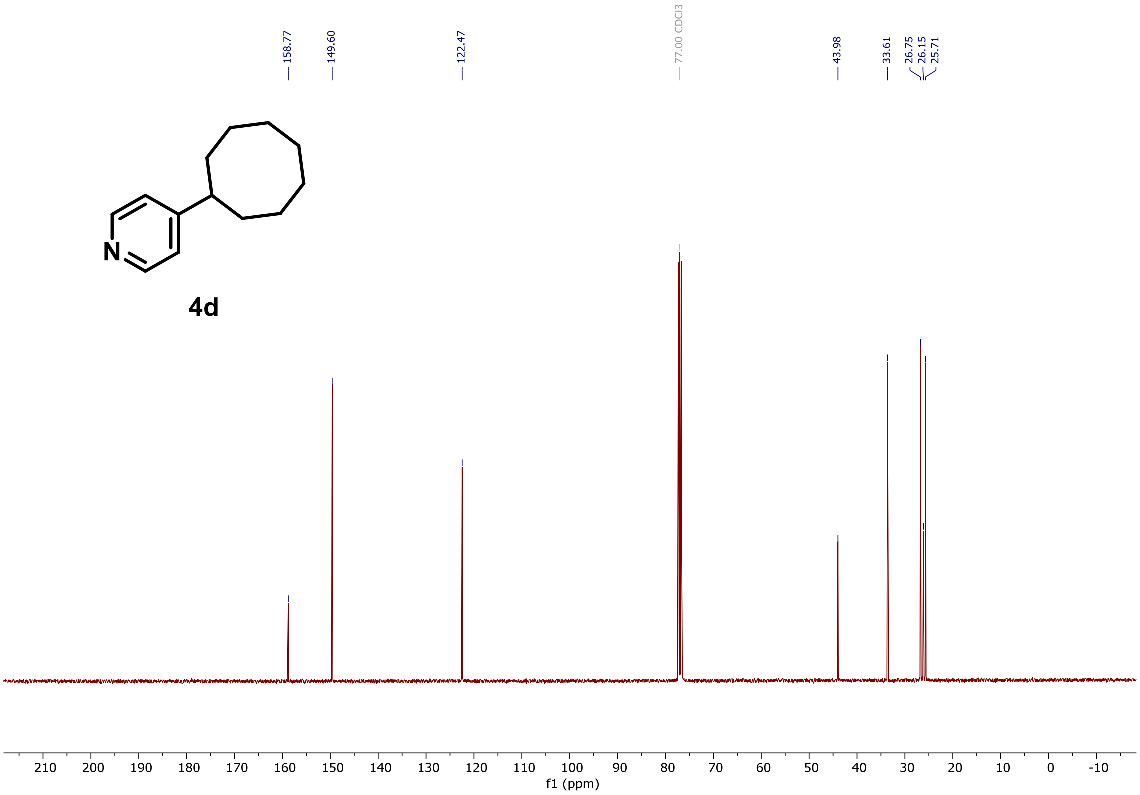

^{1}H NMR (400 MHz, $CDCl_3$) – **4e**

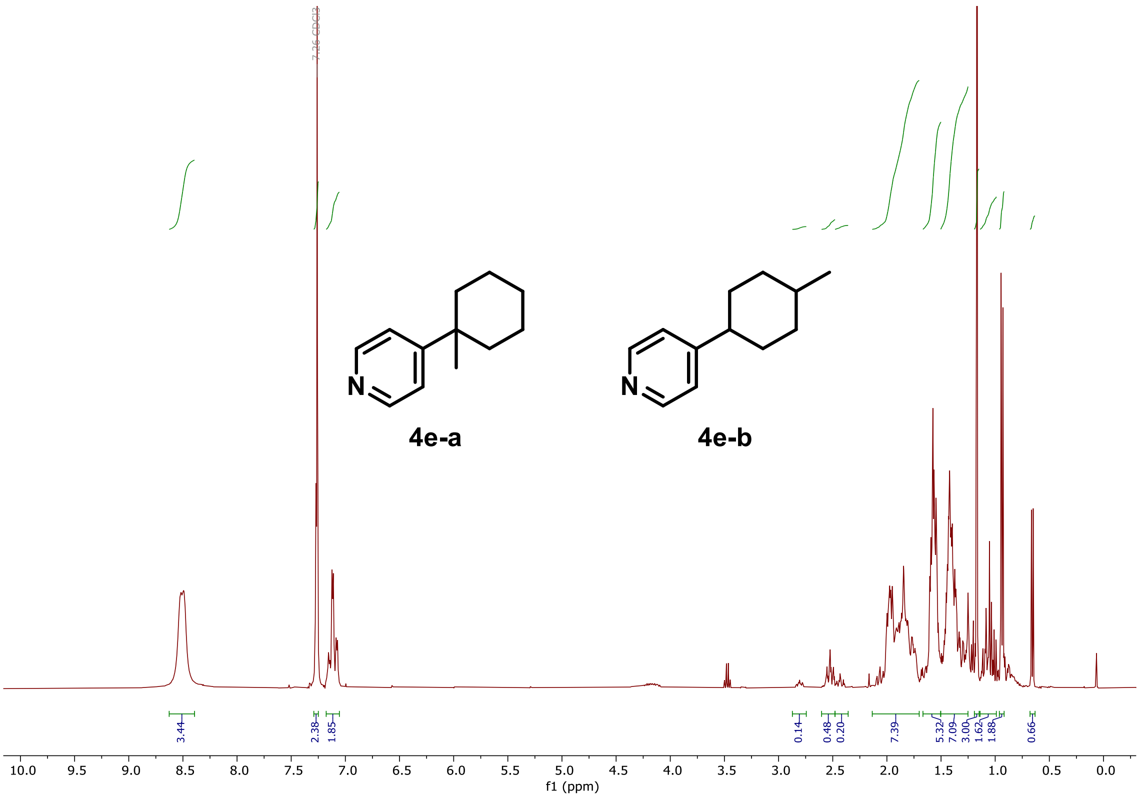

^{13}C NMR (101 MHz, $CDCl_3$) – **4e**

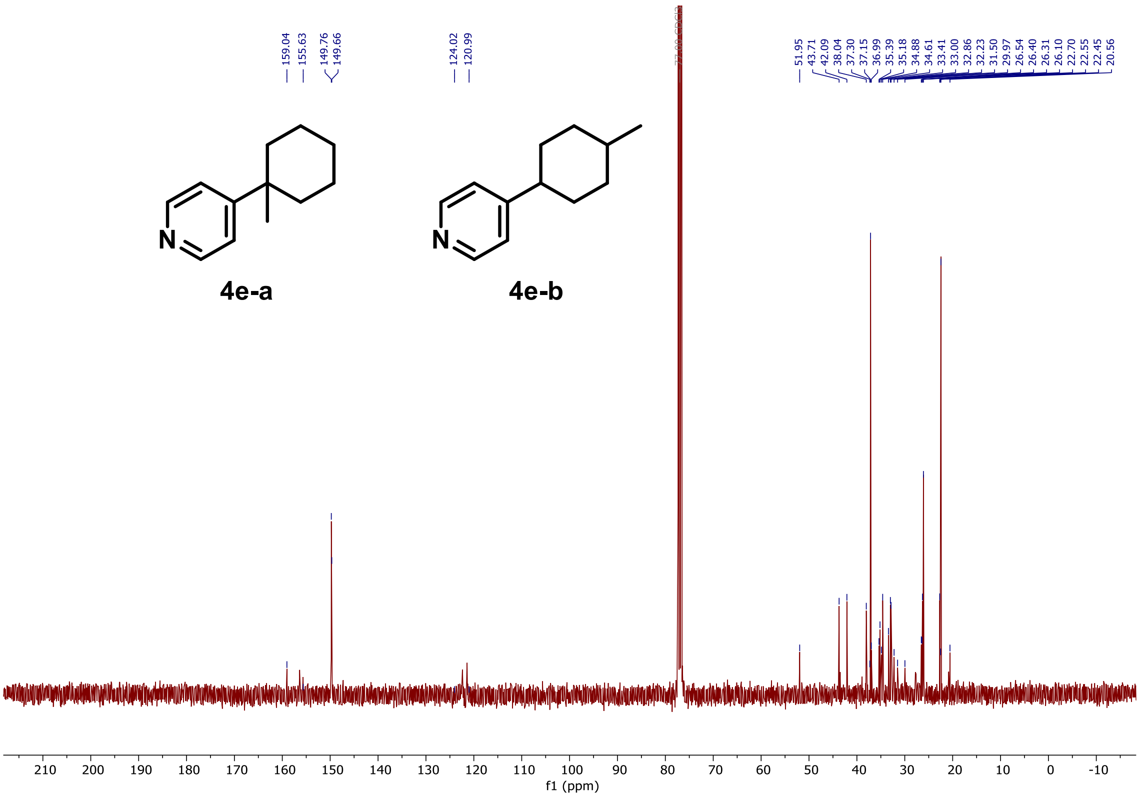

^{1}H NMR (400 MHz, $CDCl_3$) – **4f**

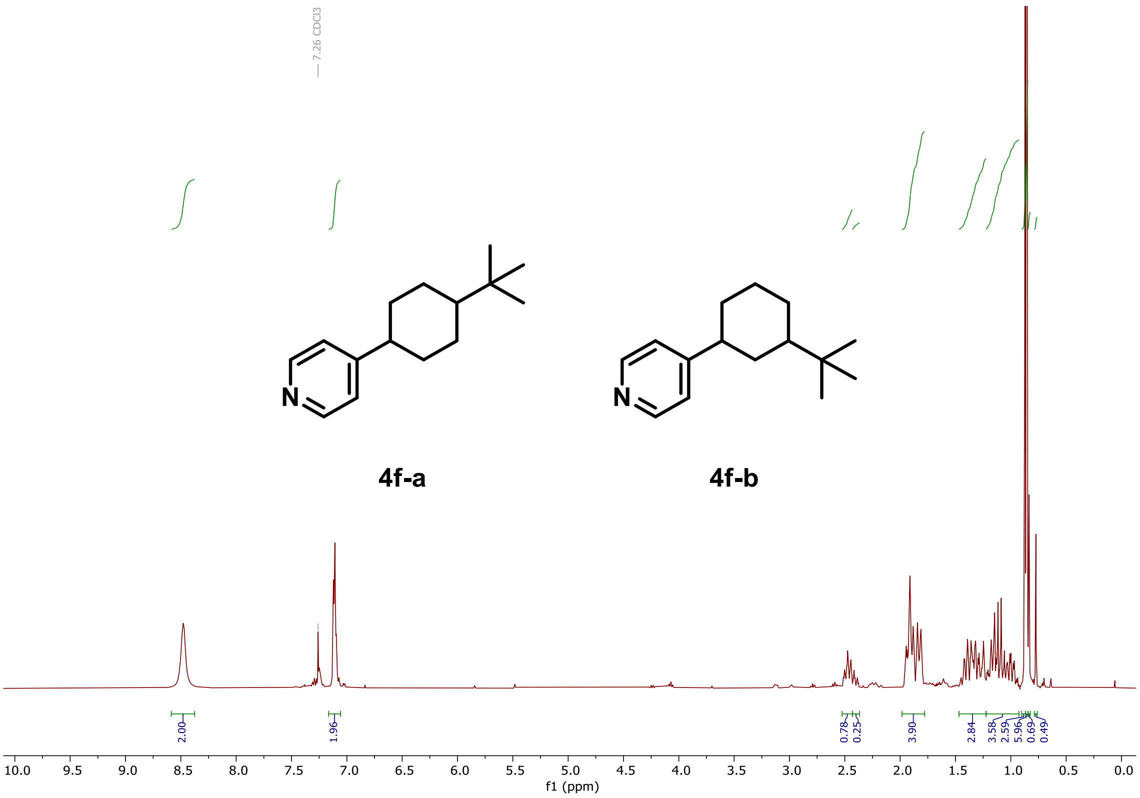

^{13}C NMR (101 MHz, $CDCl_3$) – **4f**

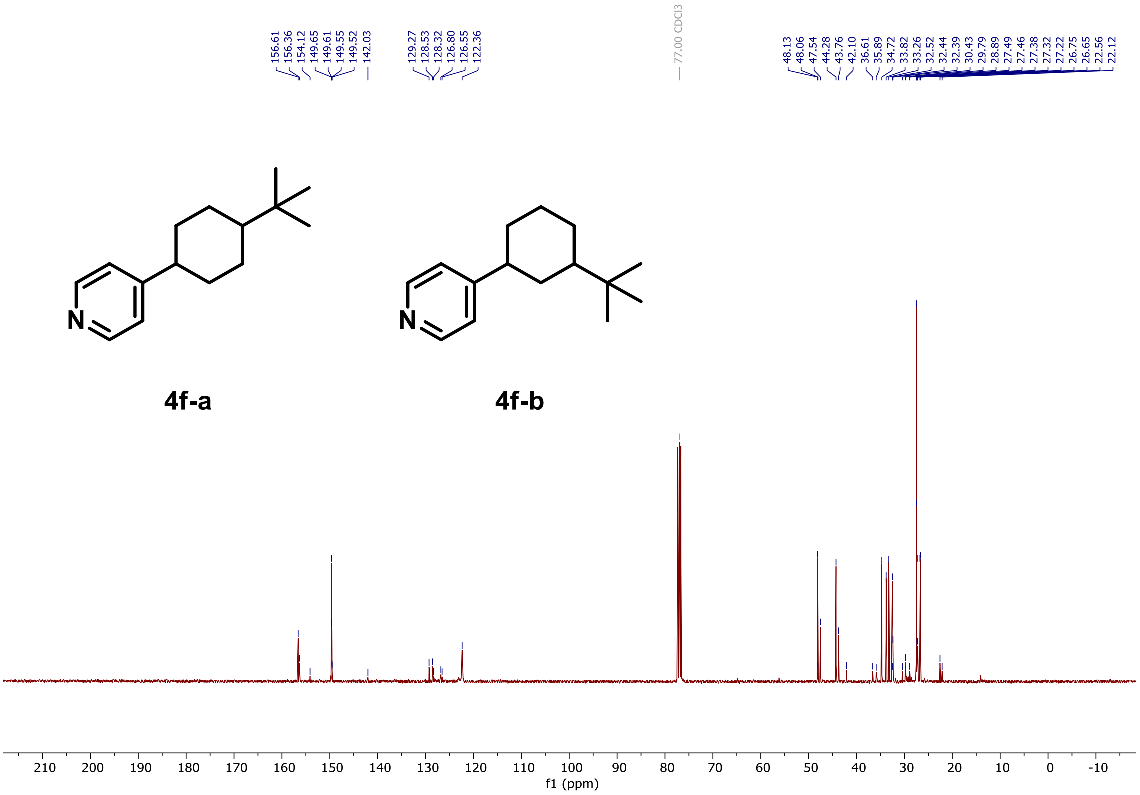

^{1}H NMR (400 MHz, $CDCl_3$) – **4g**

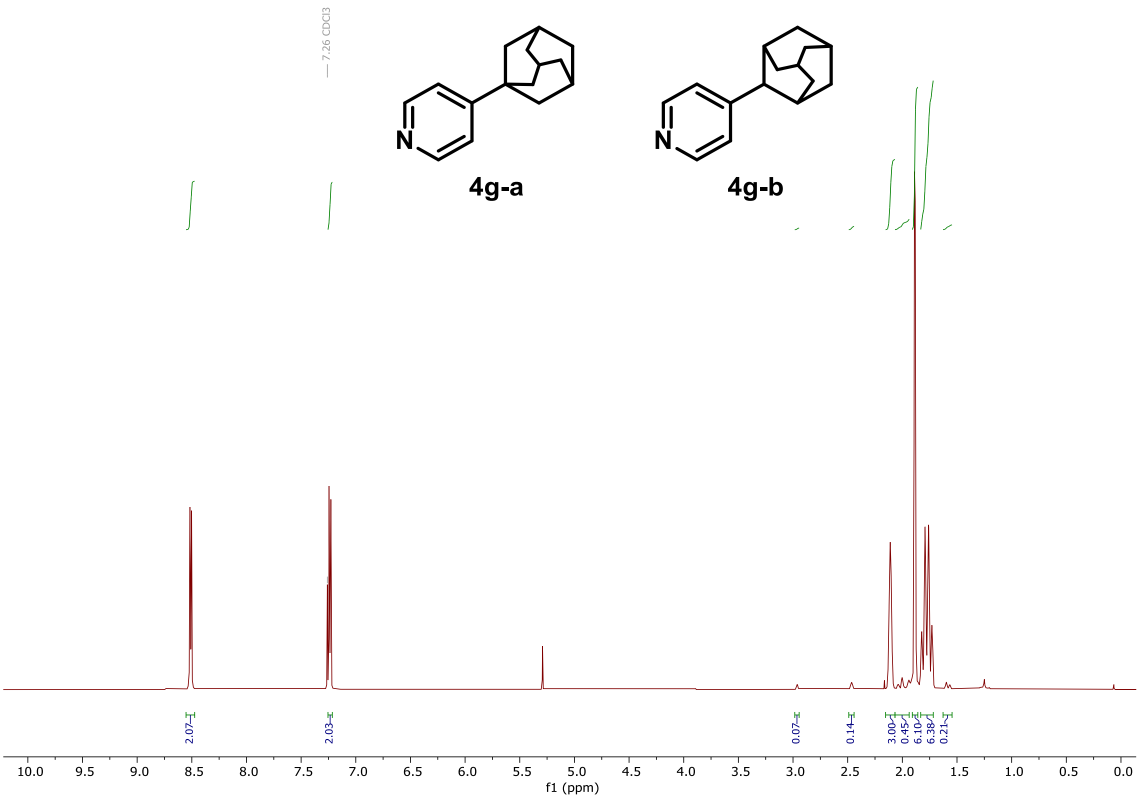

^{13}C NMR (101 MHz, $CDCl_3$) – **4g**

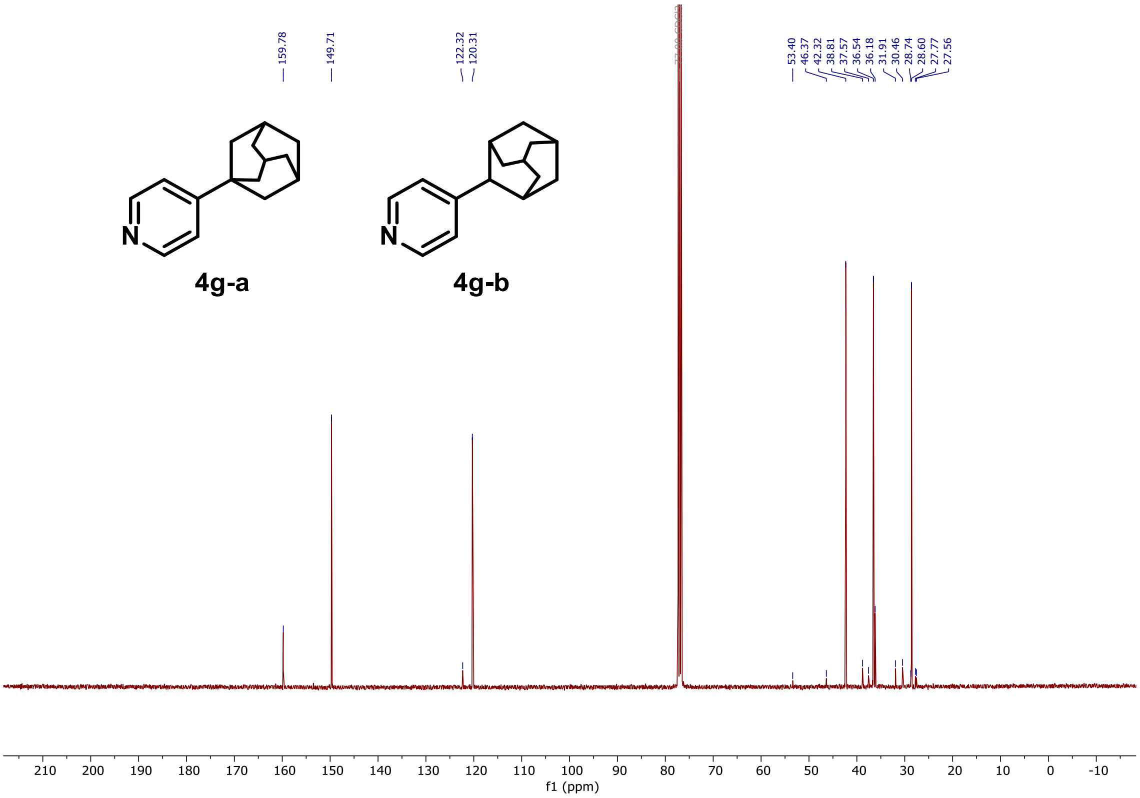

^{1}H NMR (400 MHz, $CDCl_3$) – **4h**

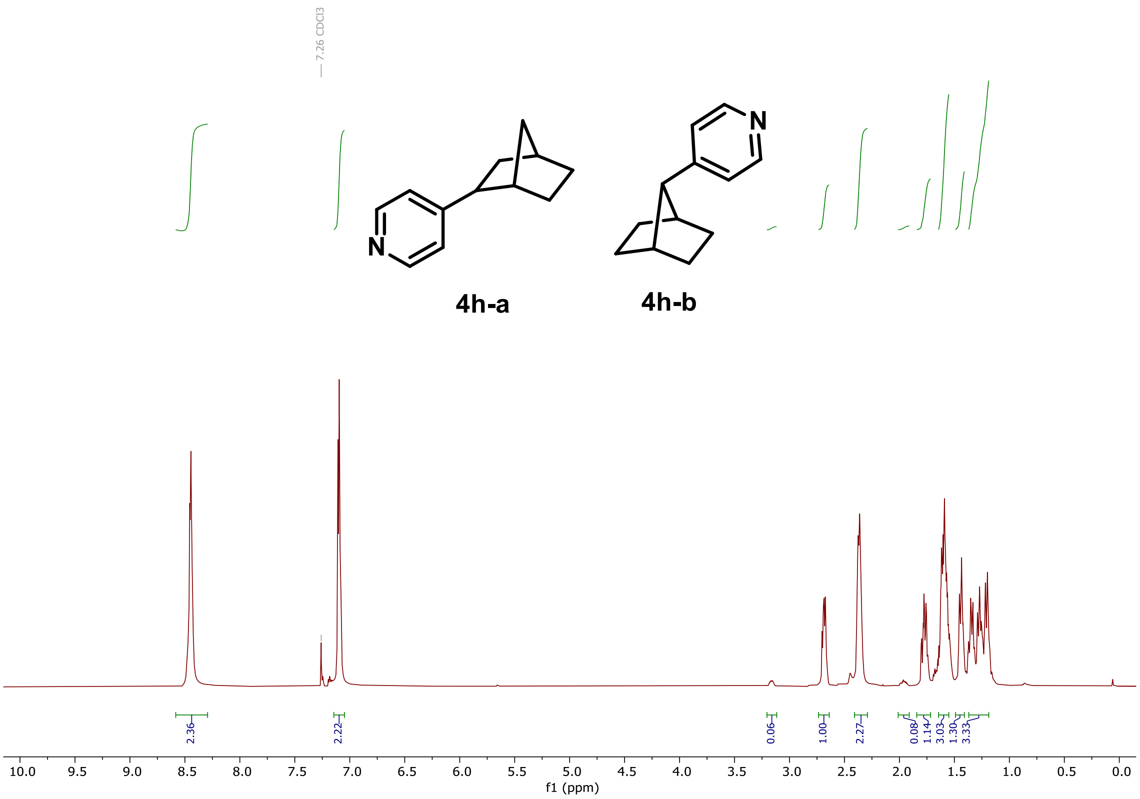

^{13}C NMR (101 MHz, $CDCl_3$) – **4h**

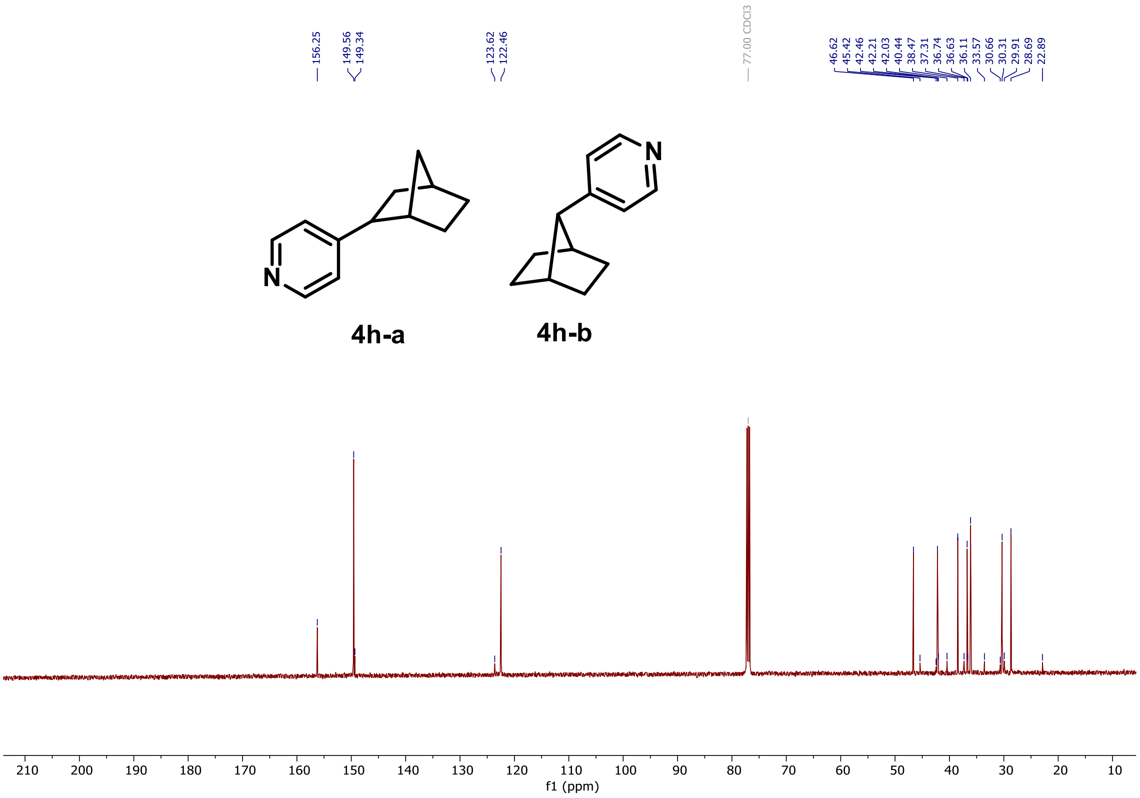

^{1}H NMR (400 MHz, $CDCl_3$) – **4i**

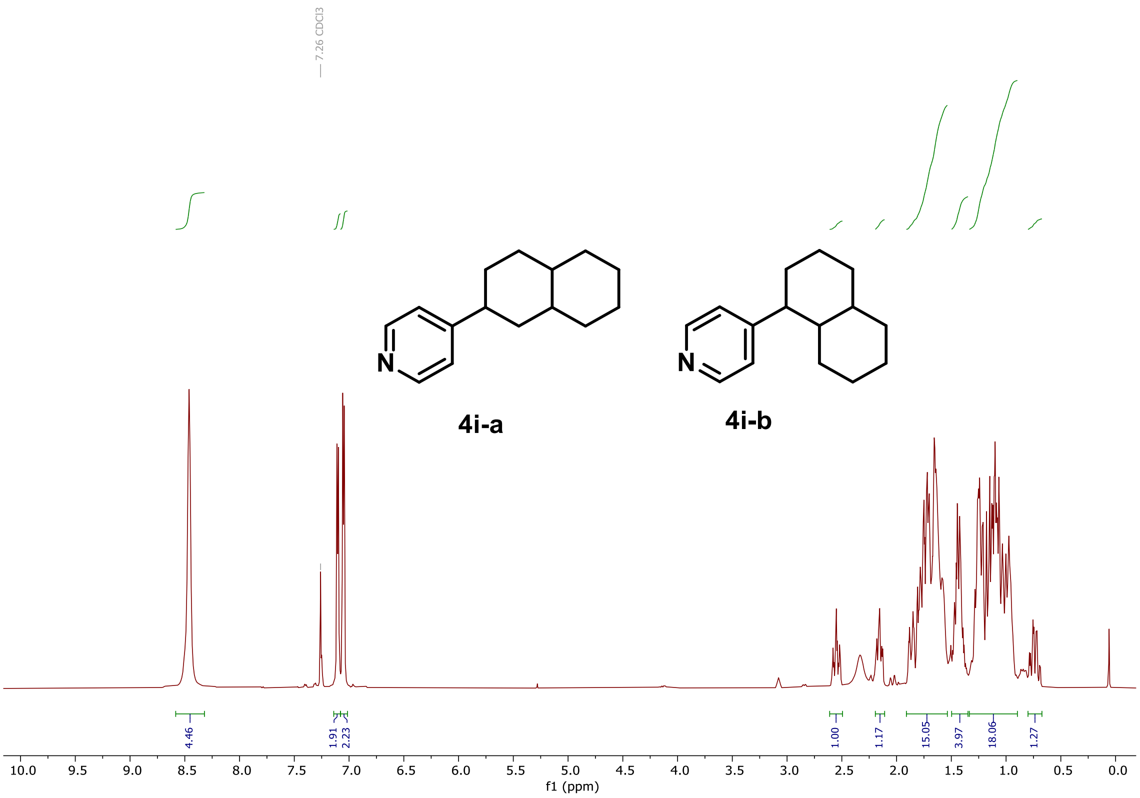

^{13}C NMR (101 MHz, $CDCl_3$) – **4i**

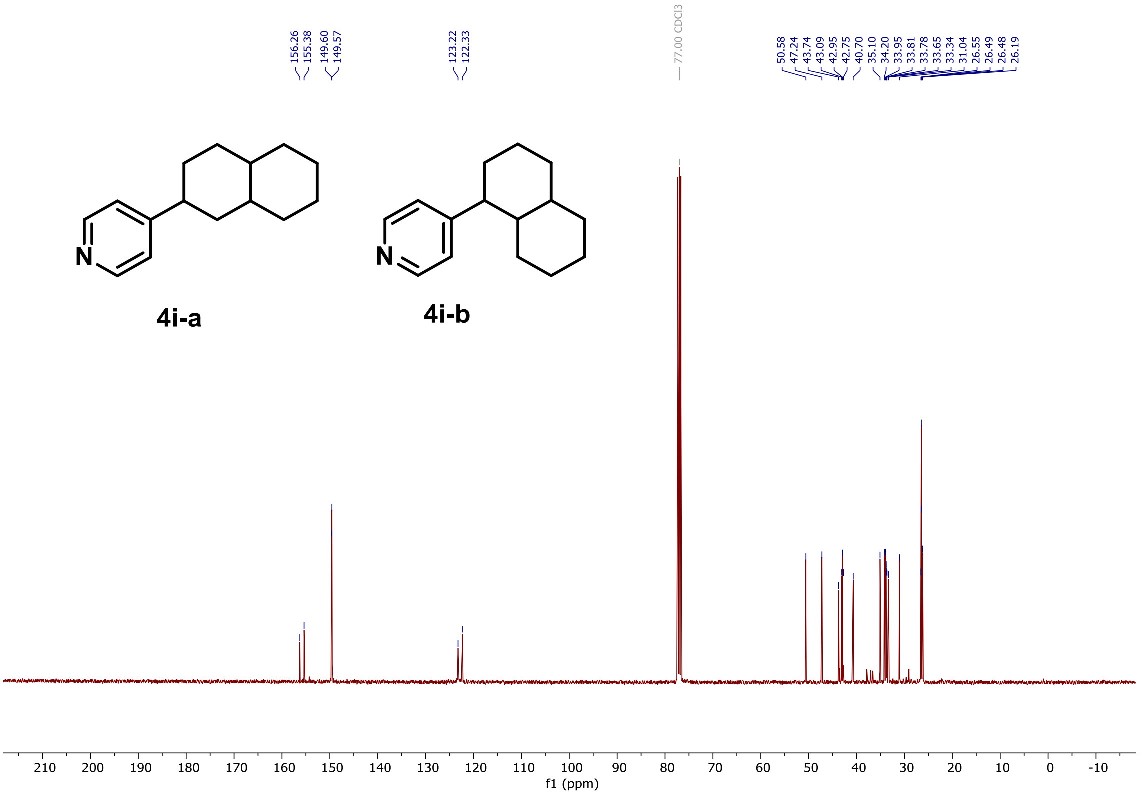

^{1}H NMR (400 MHz, $CDCl_3$) – **4j**

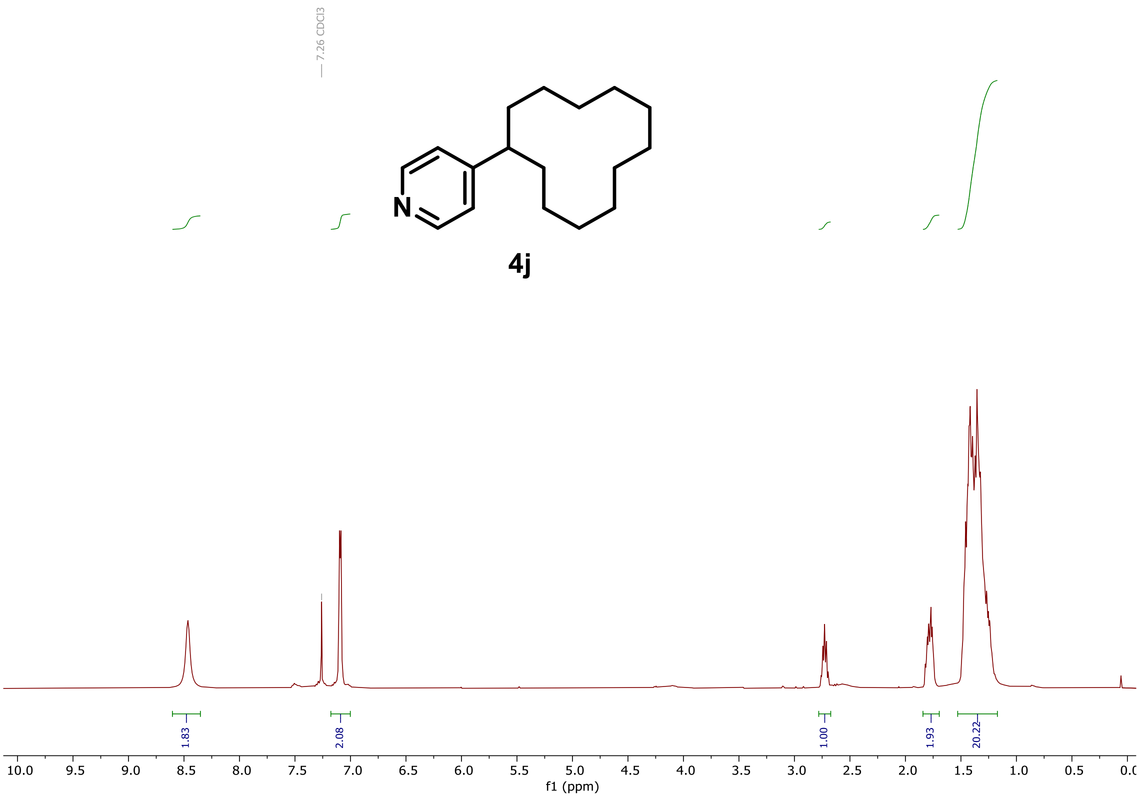

^{13}C NMR (101 MHz, $CDCl_3$) – **4j**

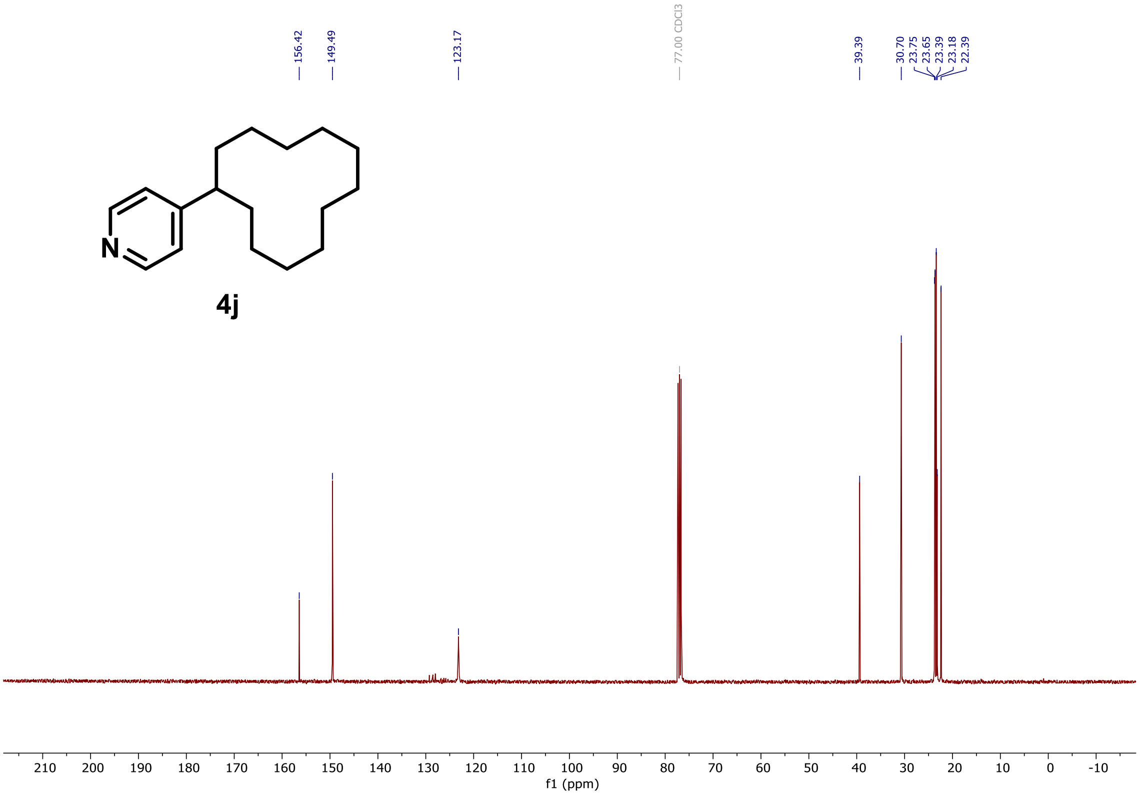

^{1}H NMR (400 MHz, $CDCl_3$) – **4k**

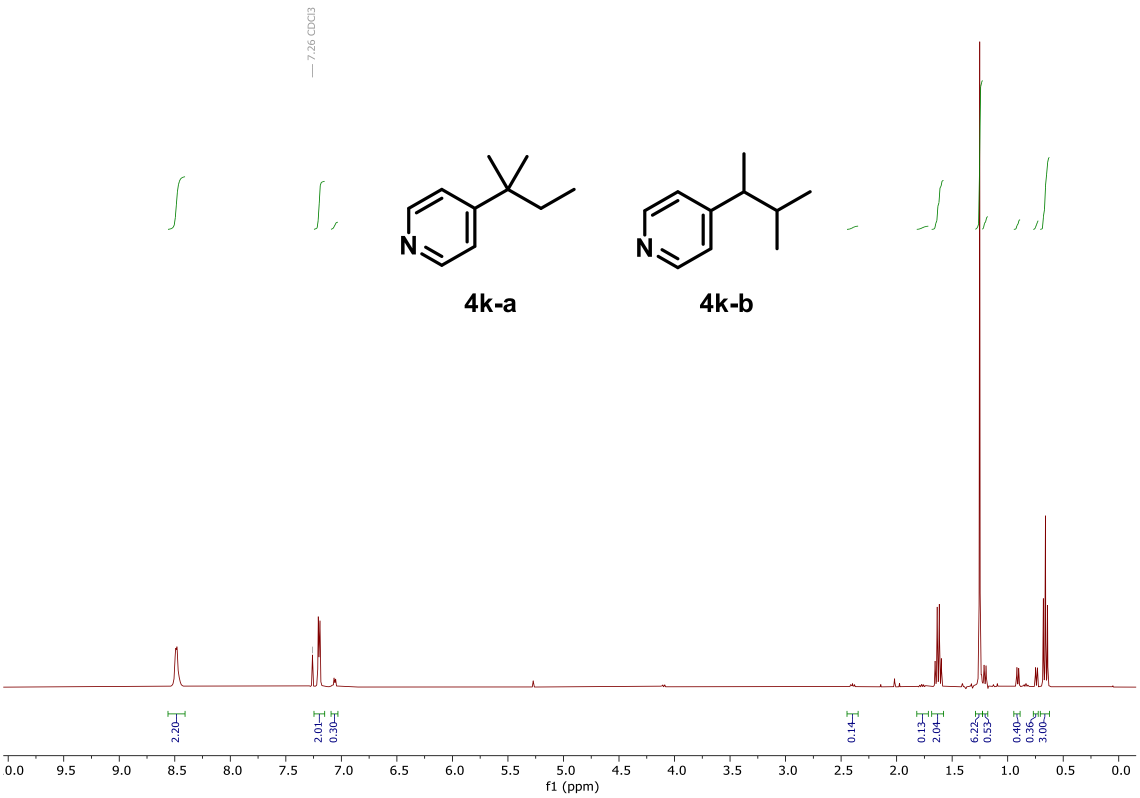

^{13}C NMR (101 MHz, $CDCl_3$) – **4k**

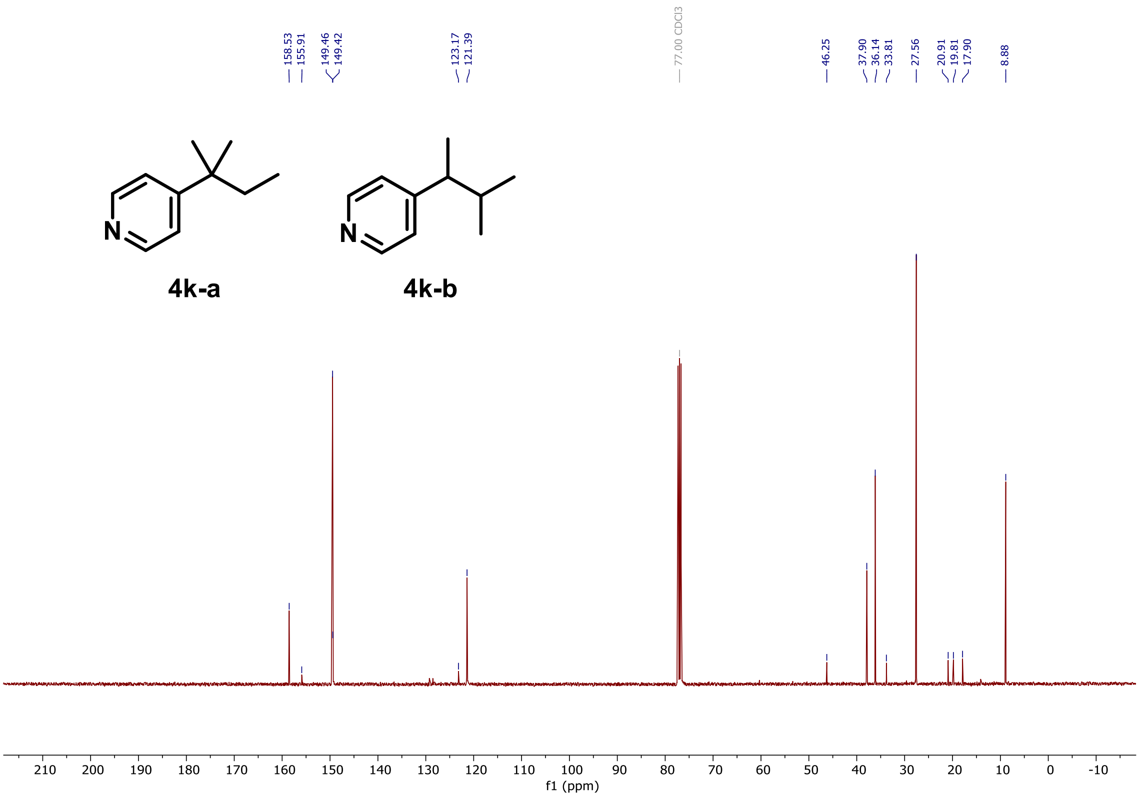

^{1}H NMR (400 MHz, $CDCl_3$) – **4l**

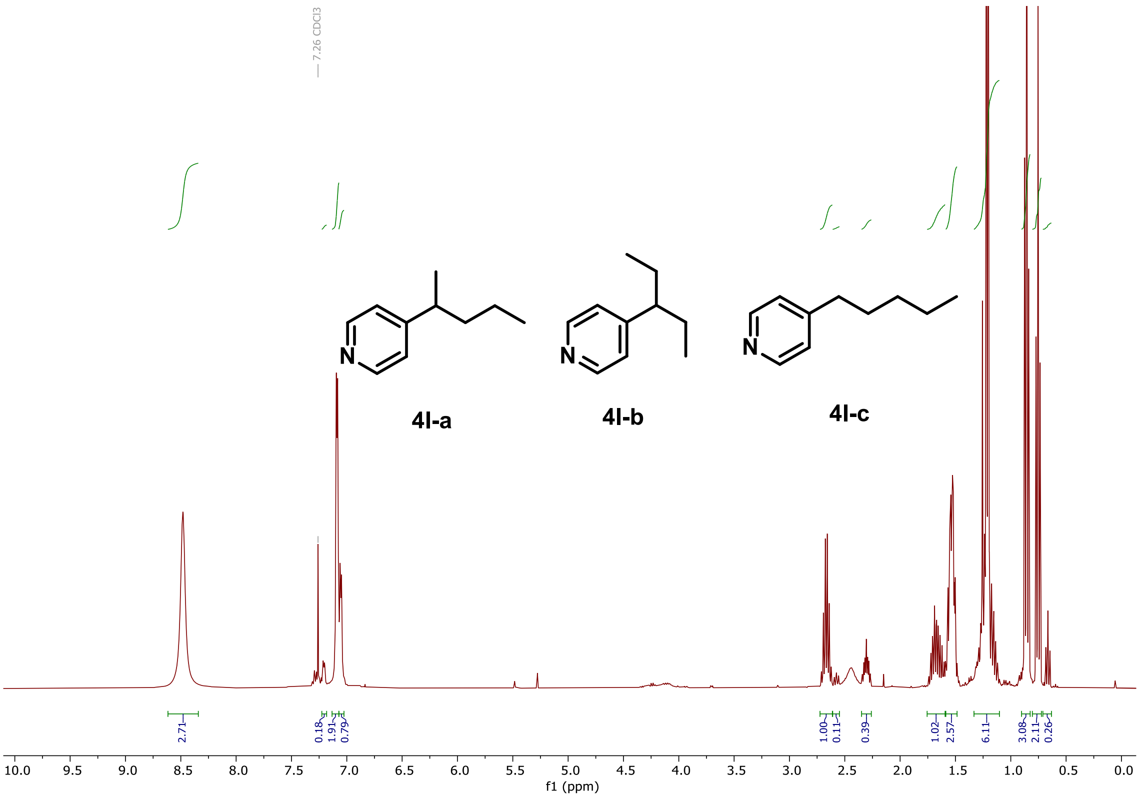

^{13}C NMR (101 MHz, $CDCl_3$) – **4l**

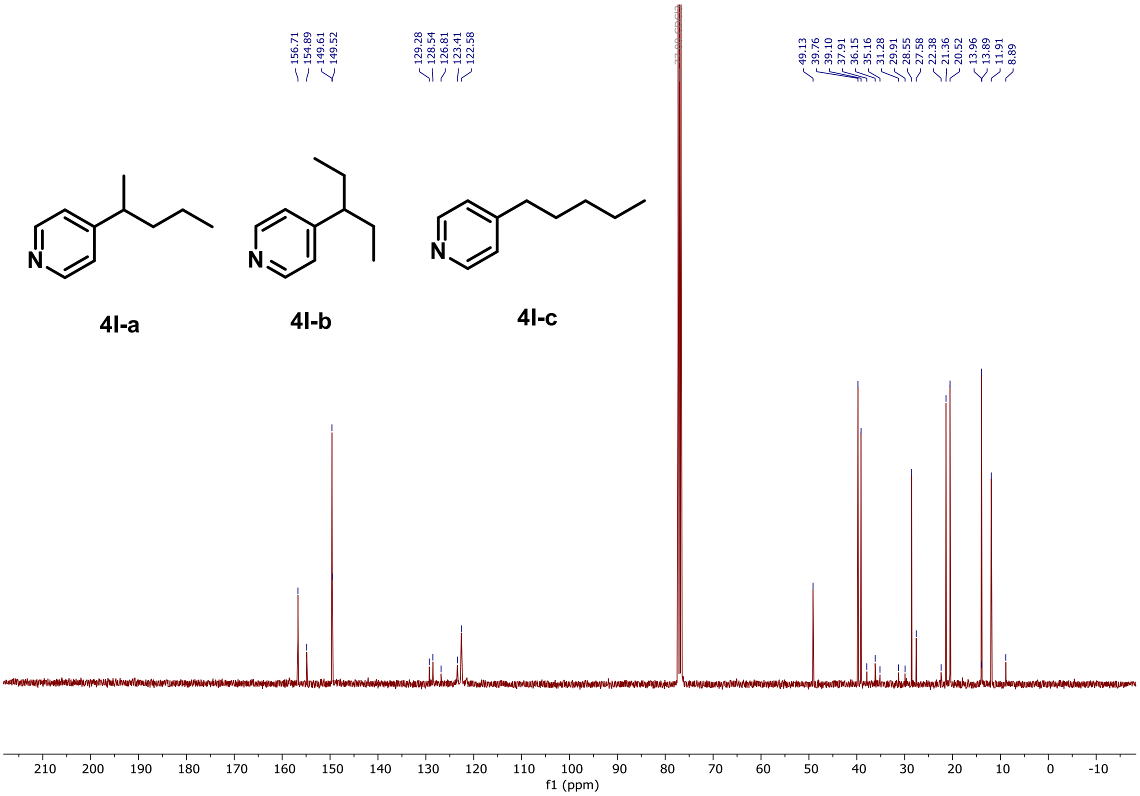

^{1}H NMR (400 MHz, $CDCl_3$) – **4m**

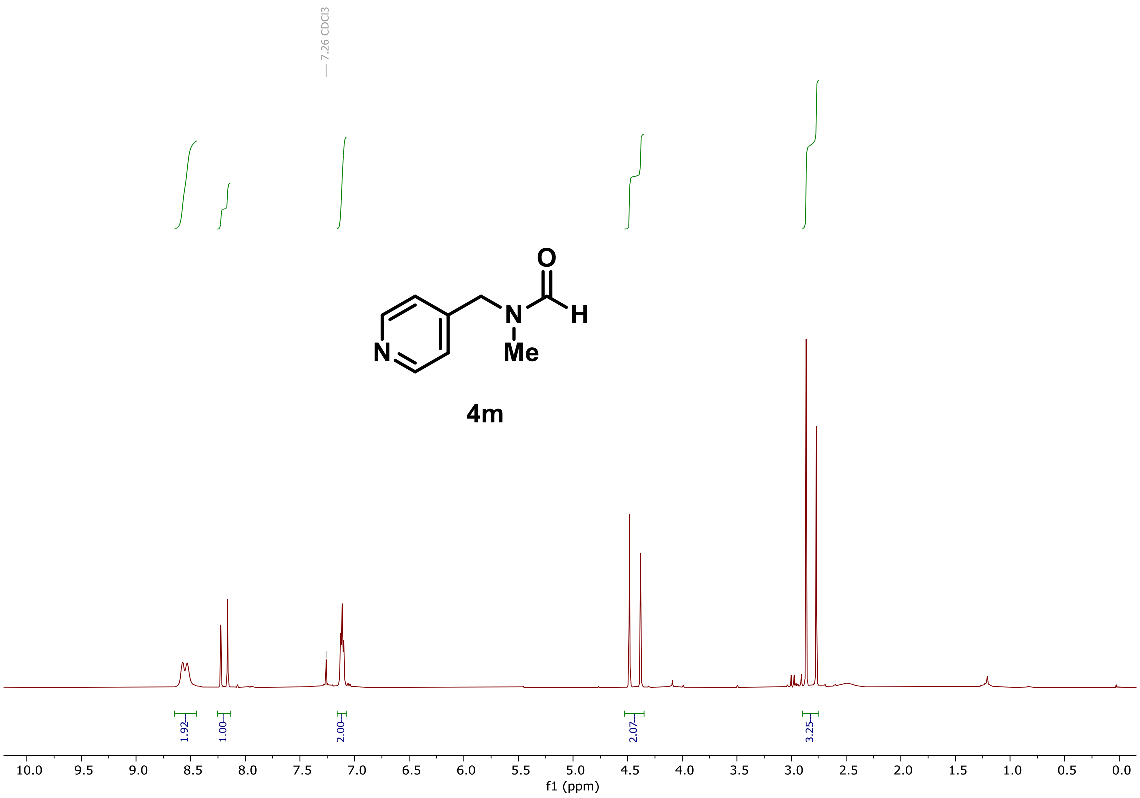

^{13}C NMR (101 MHz, $CDCl_3$) – **4m**

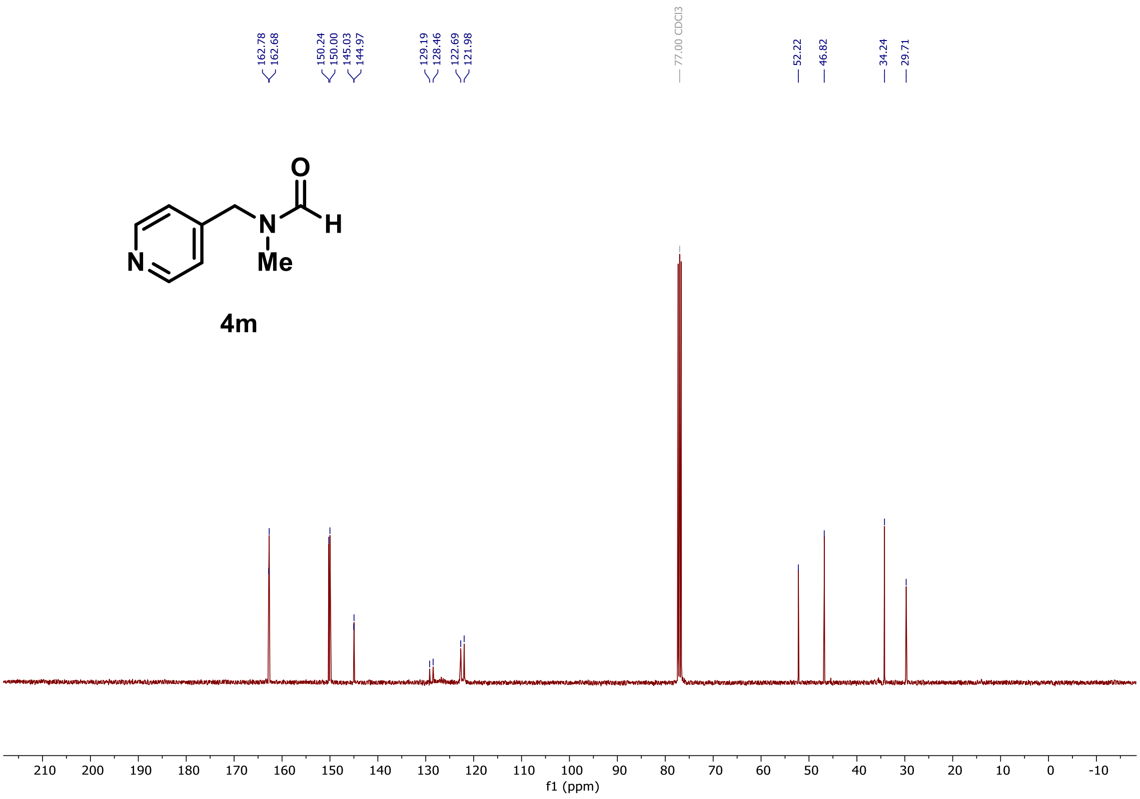

^{1}H NMR (400 MHz, $CDCl_3$) – **4n**

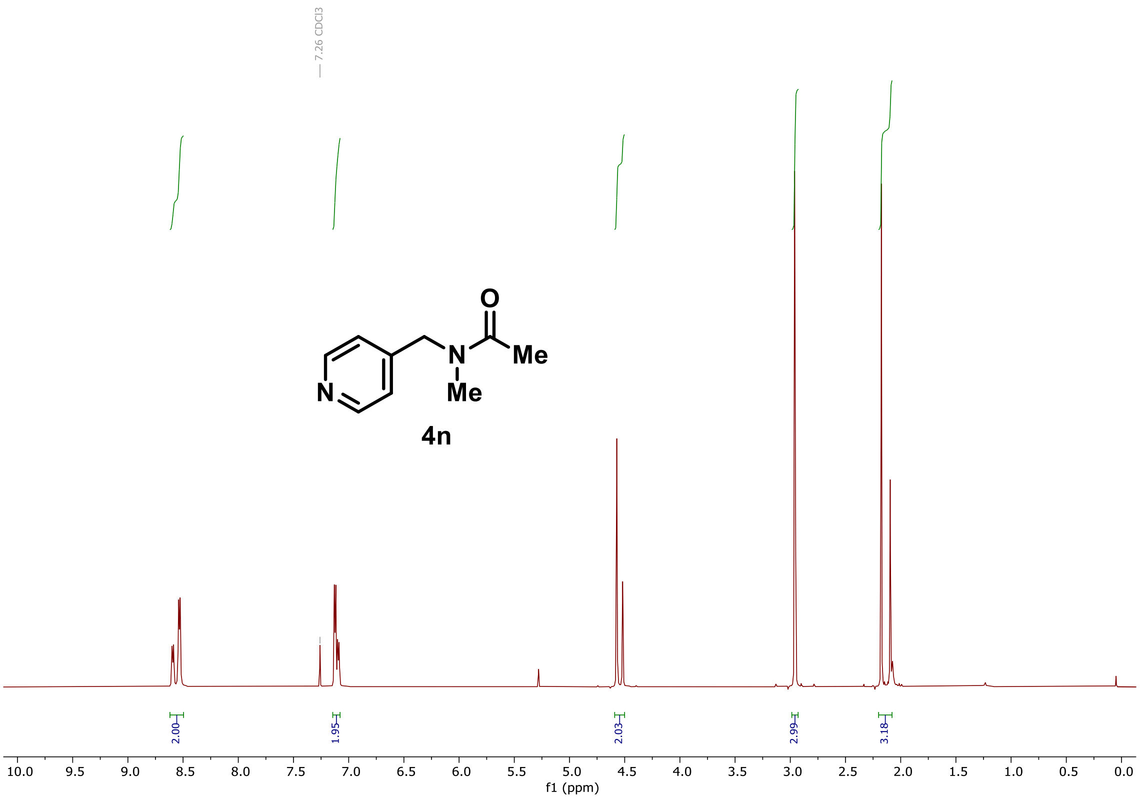

^{13}C NMR (101 MHz, $CDCl_3$) – **4n**

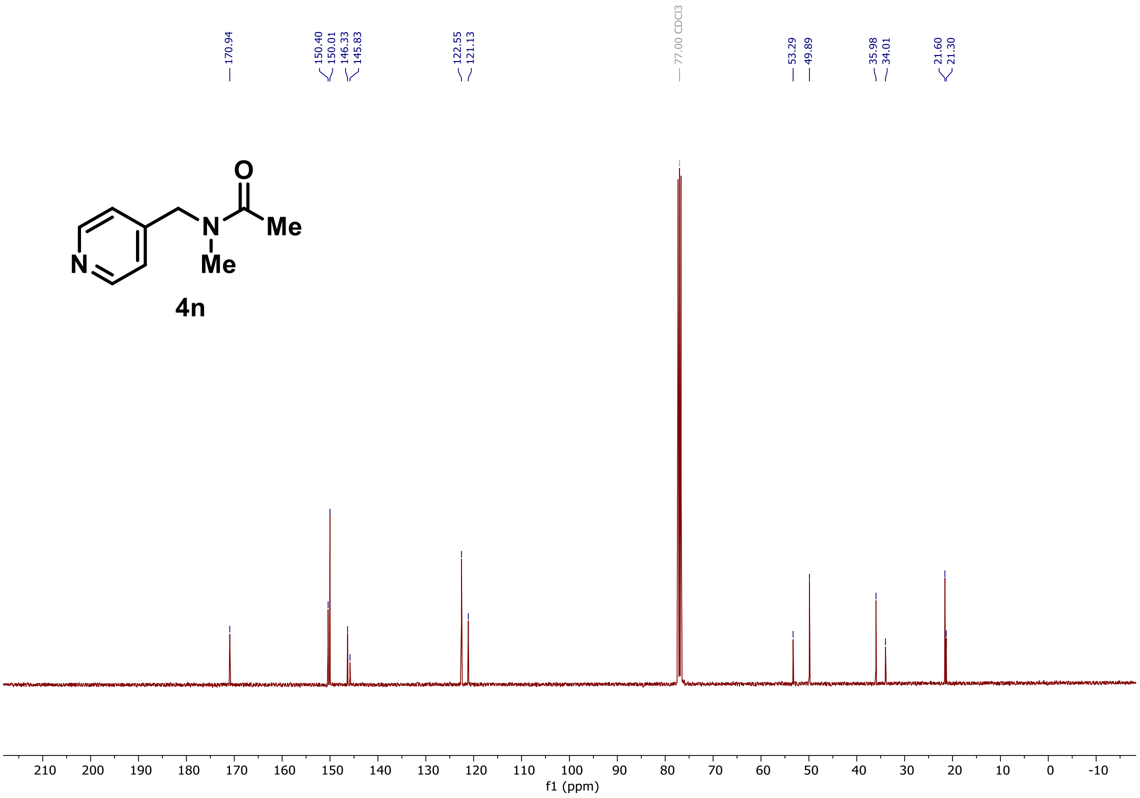

^{1}H NMR (400 MHz, $CDCl_3$) – **4o**

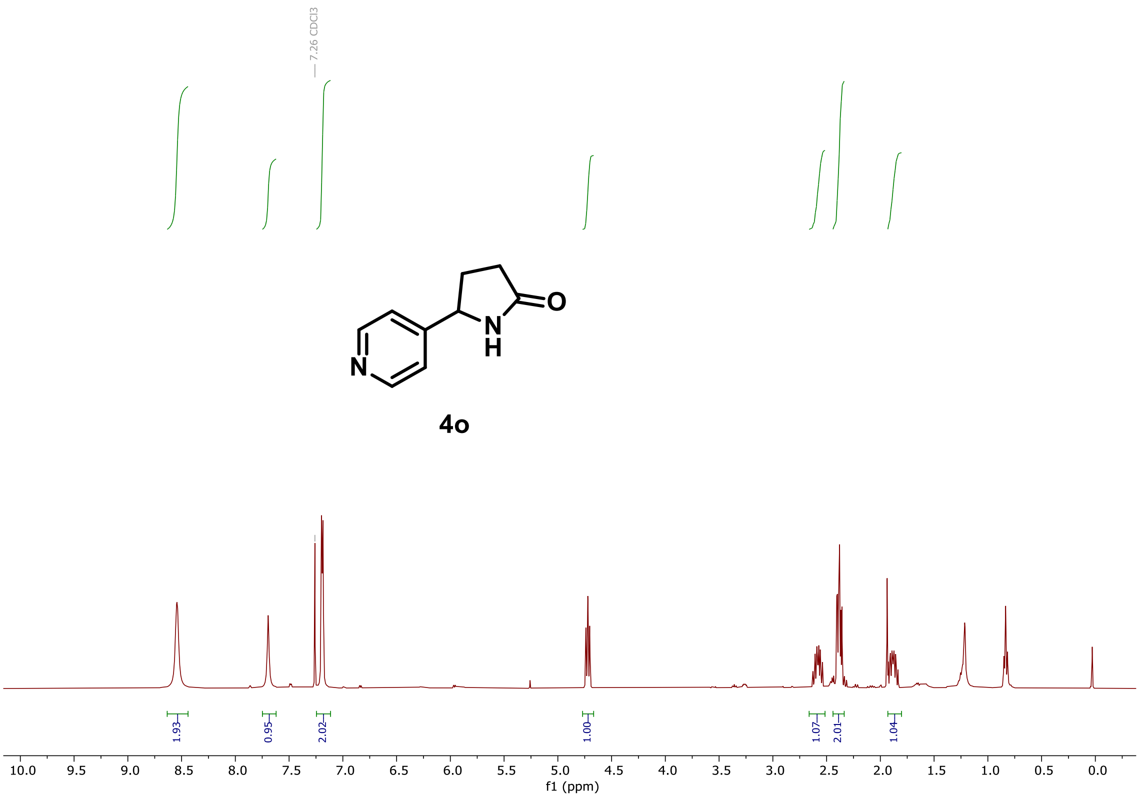

^{13}C NMR (101 MHz, $CDCl_3$) – **4o**

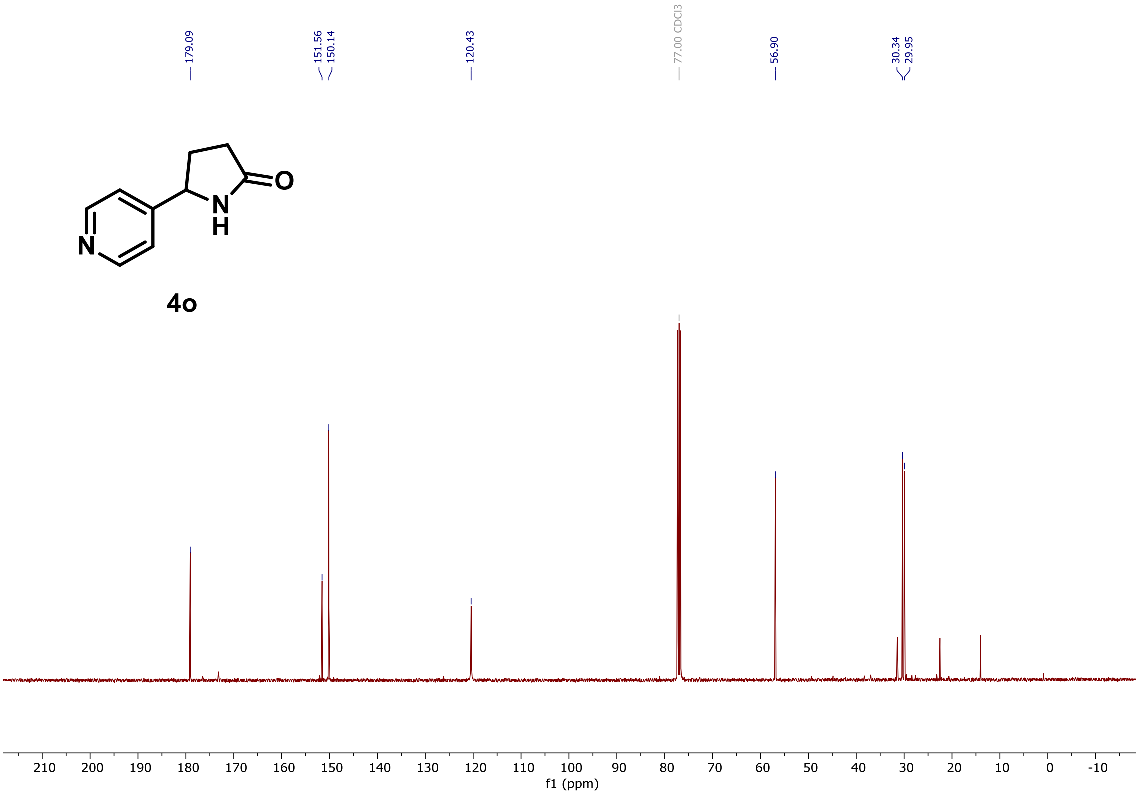

^{1}H NMR (400 MHz, $CDCl_3$) – **4p**

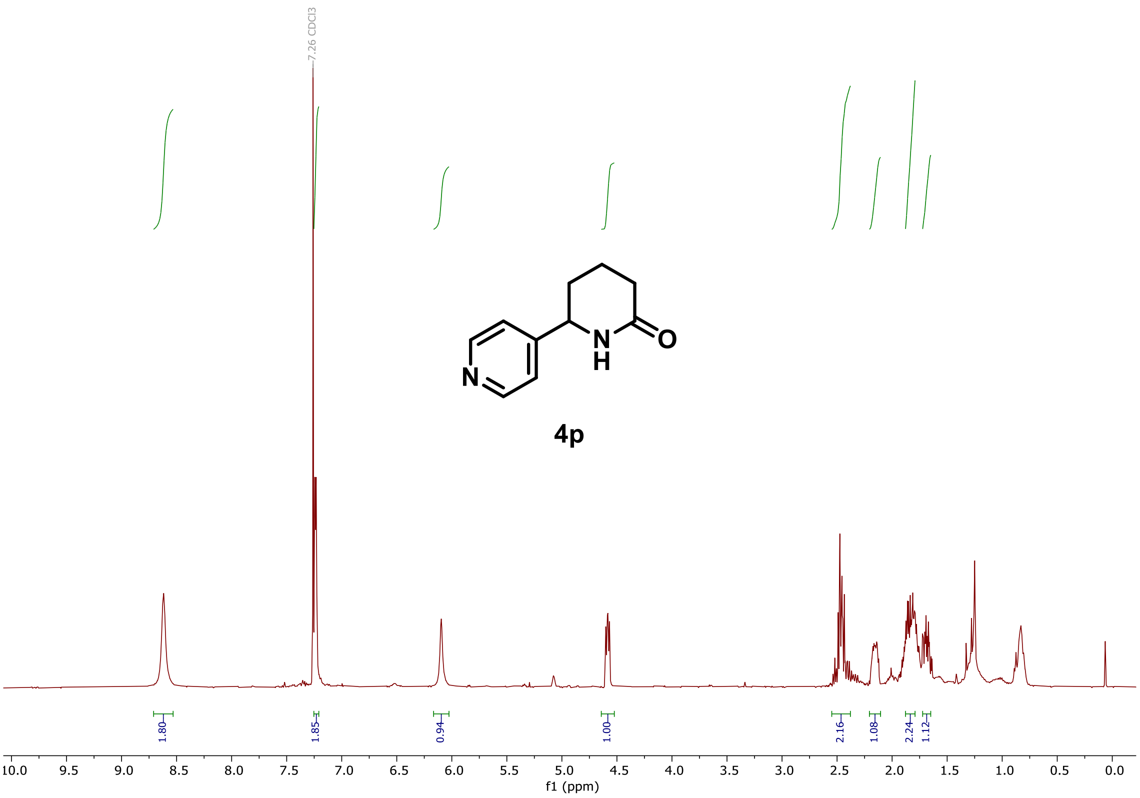

^{13}C NMR (101 MHz, $CDCl_3$) – **4p**

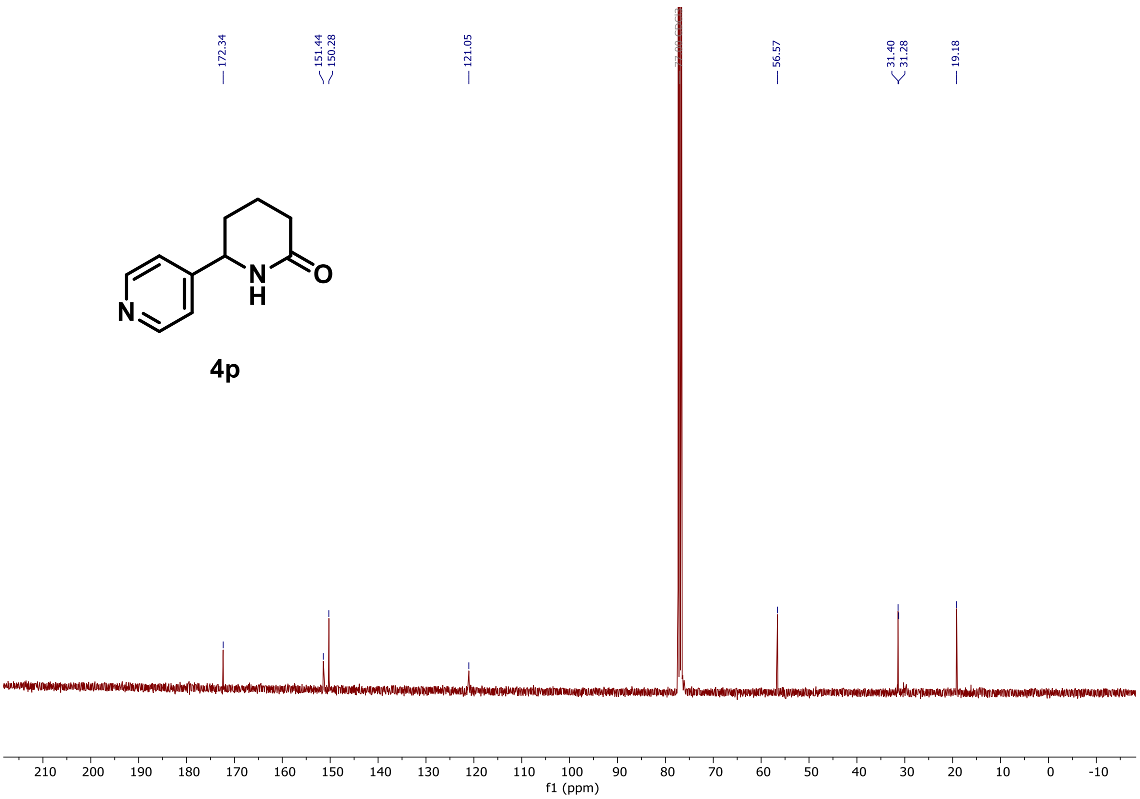

^{1}H NMR (400 MHz, $CDCl_3$) – **4q**

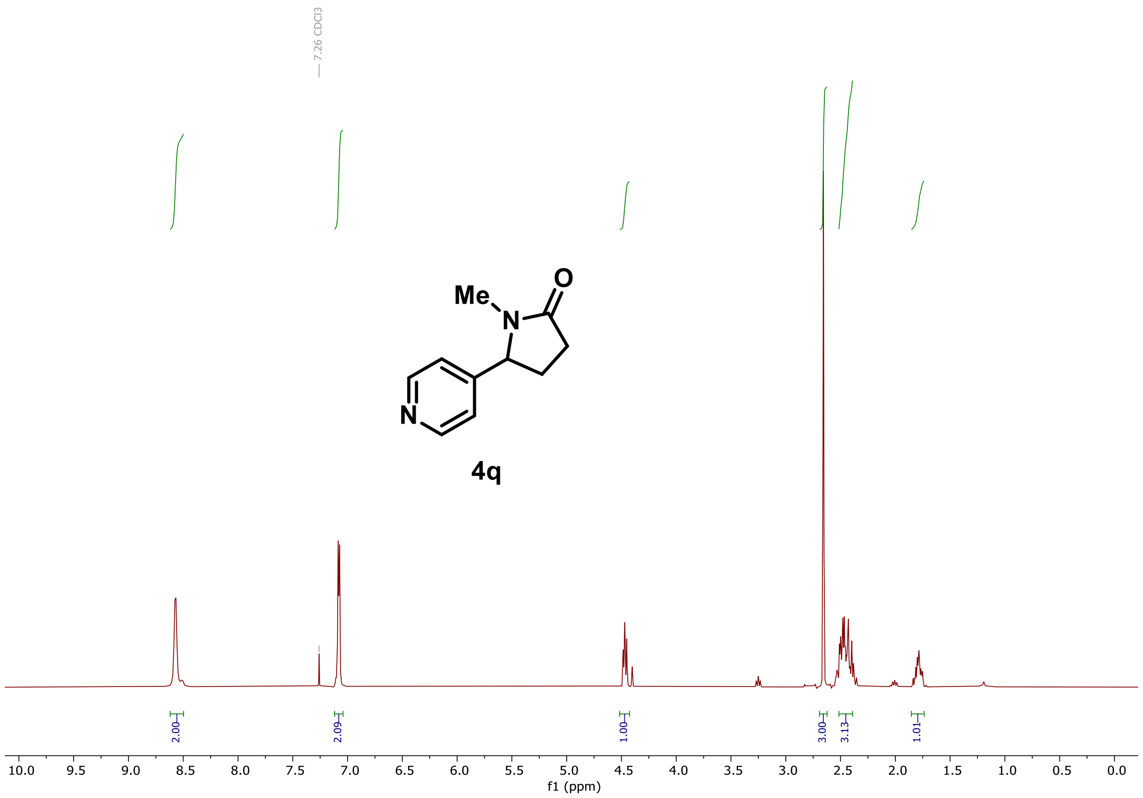

^{13}C NMR (101 MHz, $CDCl_3$) – **4q**

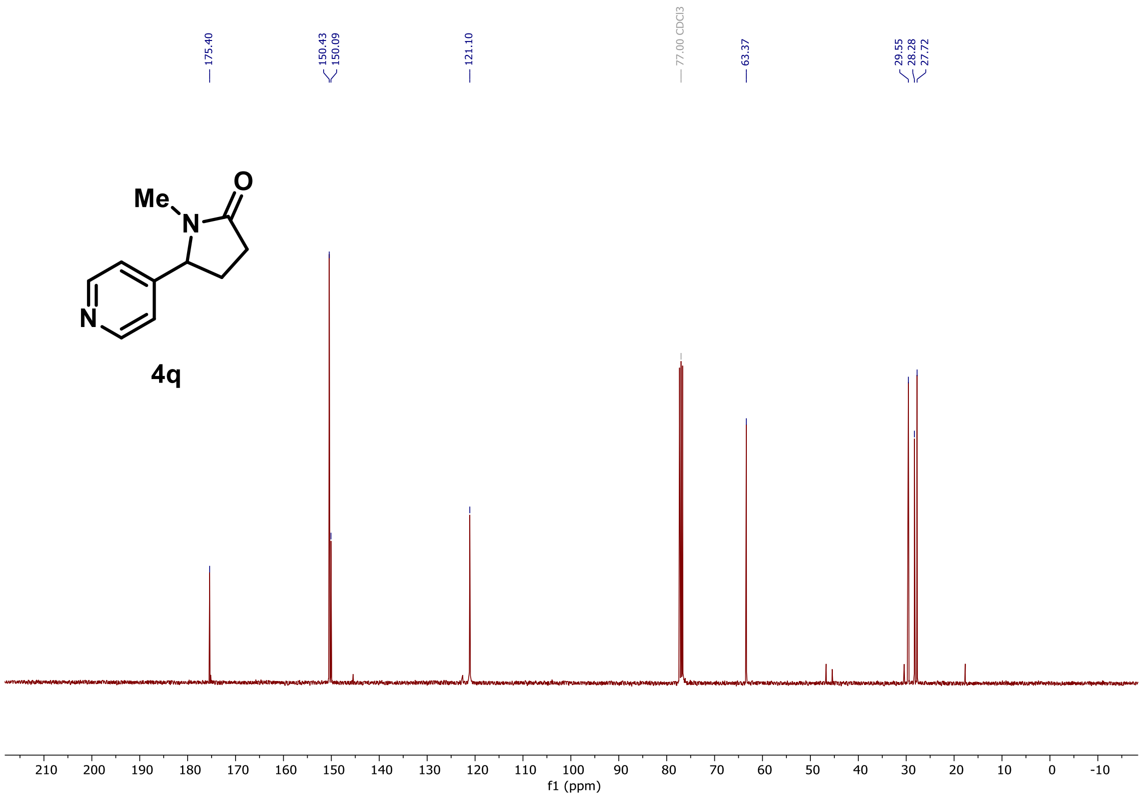

^{1}H NMR (400 MHz, $CDCl_3$) – **4r**

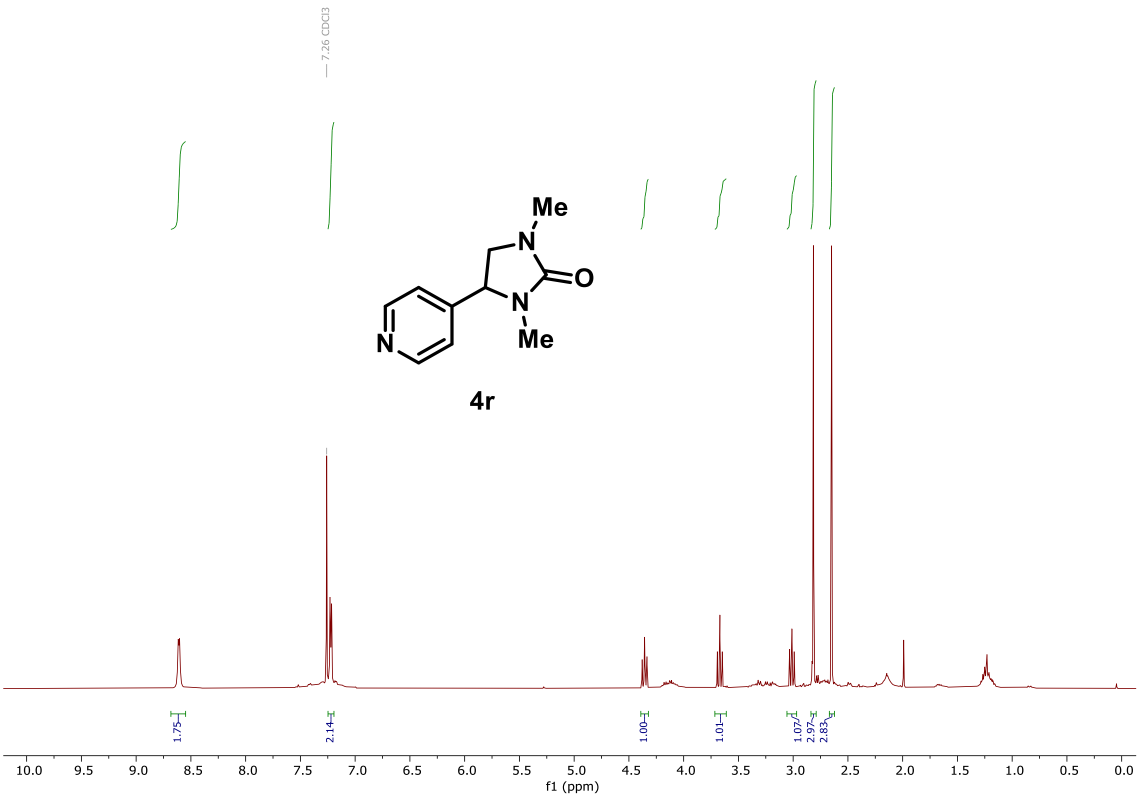

^{13}C NMR (101 MHz, $CDCl_3$) – **4r**

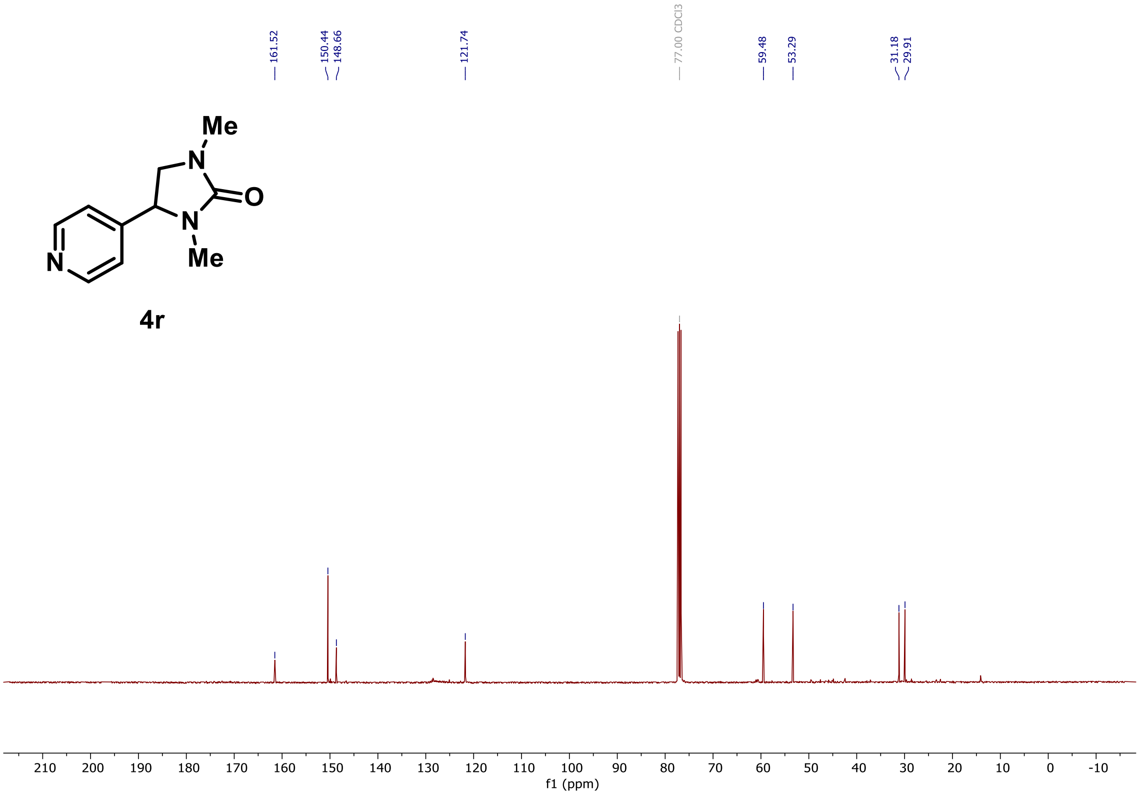

^{1}H NMR (400 MHz, $CDCl_3$) – **4s**

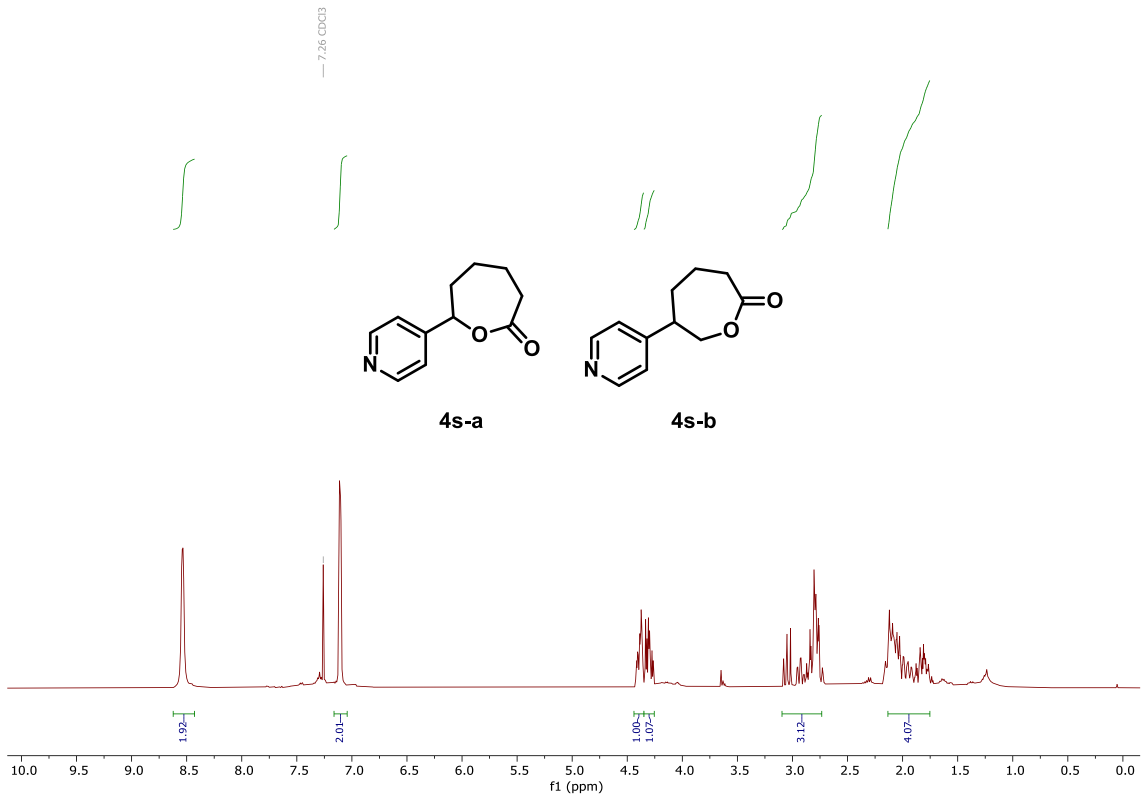

^{13}C NMR (101 MHz, $CDCl_3$) – **4s**

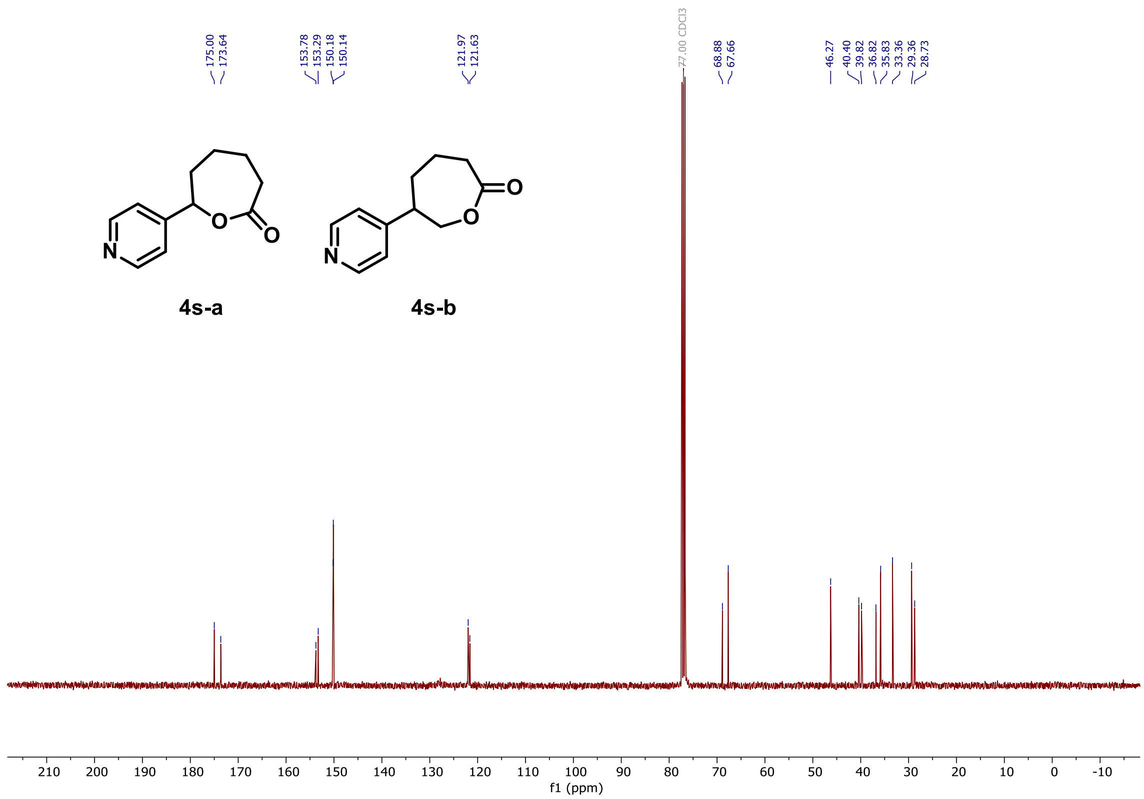

^{1}H NMR (400 MHz, $CDCl_3$) – **4t**

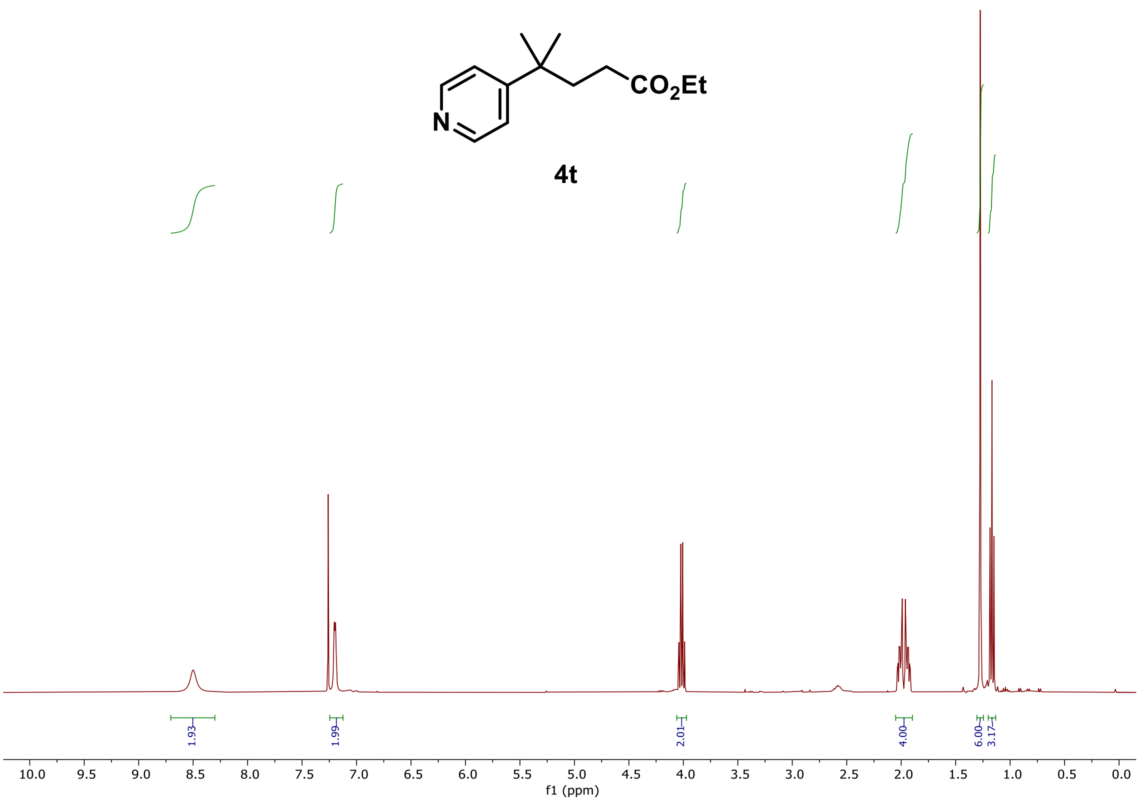

^{13}C NMR (101 MHz, $CDCl_3$) – **4t**

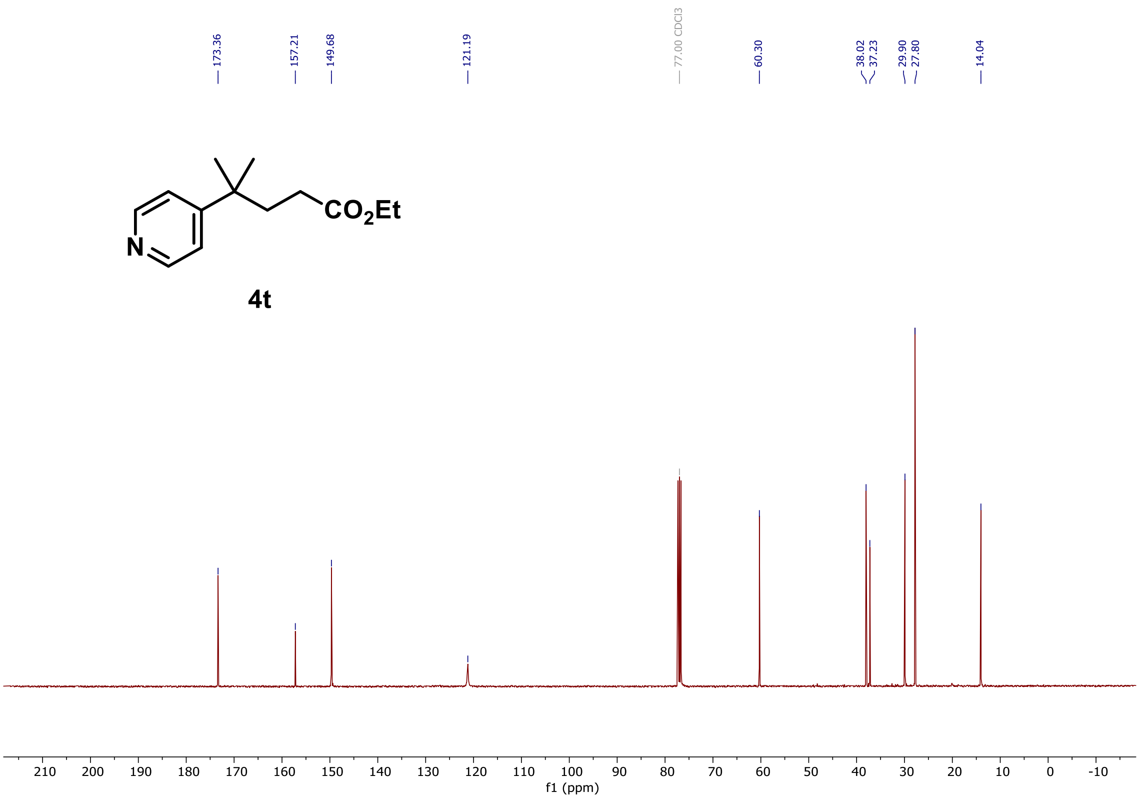

^{1}H NMR (400 MHz, $CDCl_3$) – **4u**

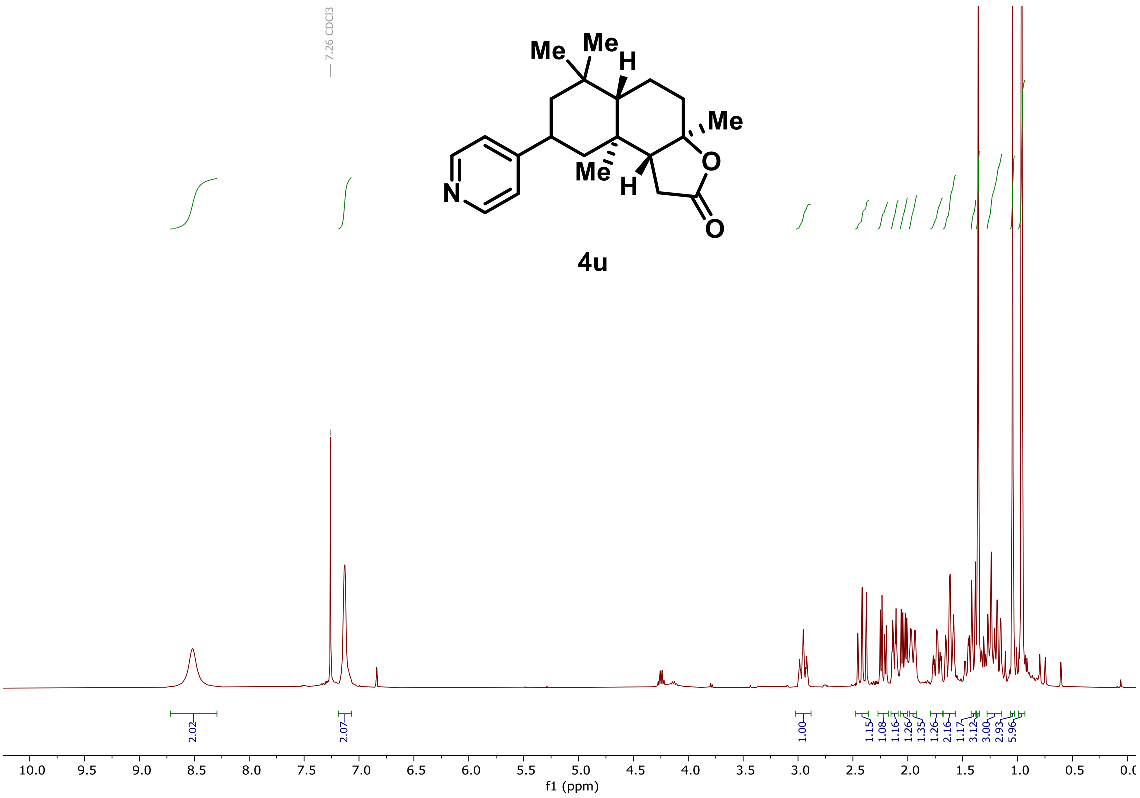

^{13}C NMR (101 MHz, $CDCl_3$) – **4u**

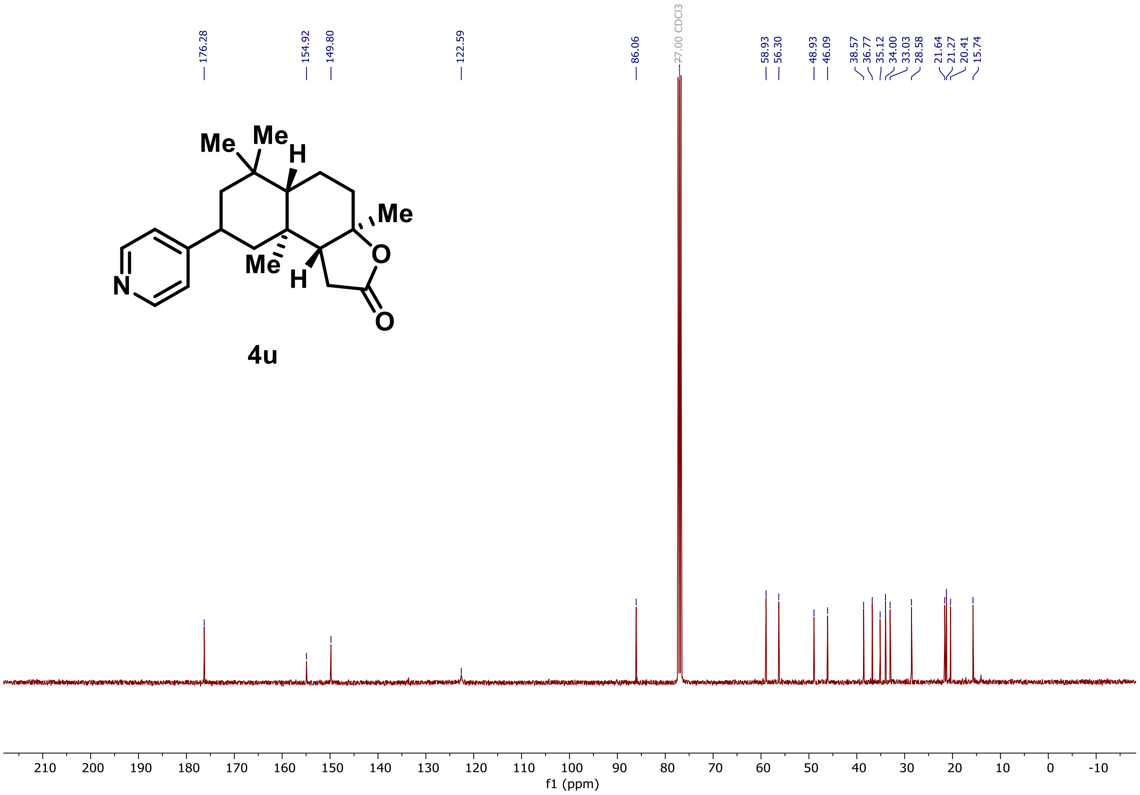

^1H NMR (400 MHz, $CDCl_3$) – **4v**

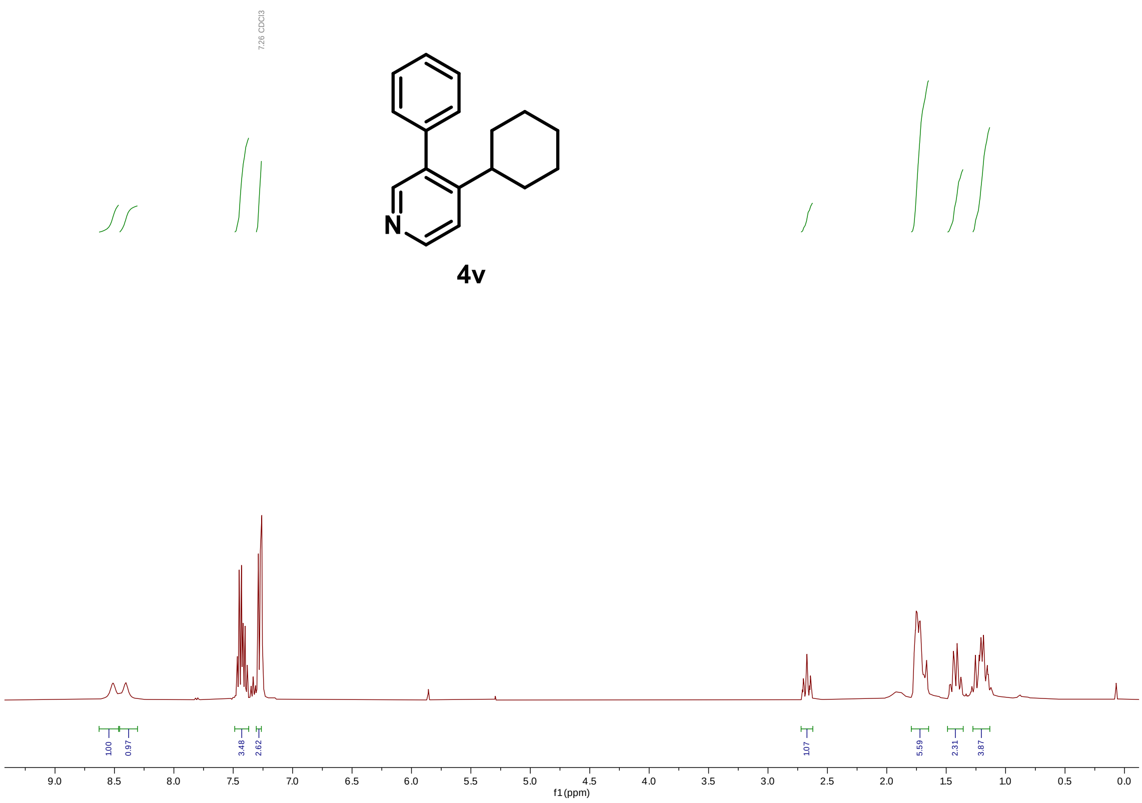

^{13}C NMR (101 MHz, $CDCl_3$) – **4v**

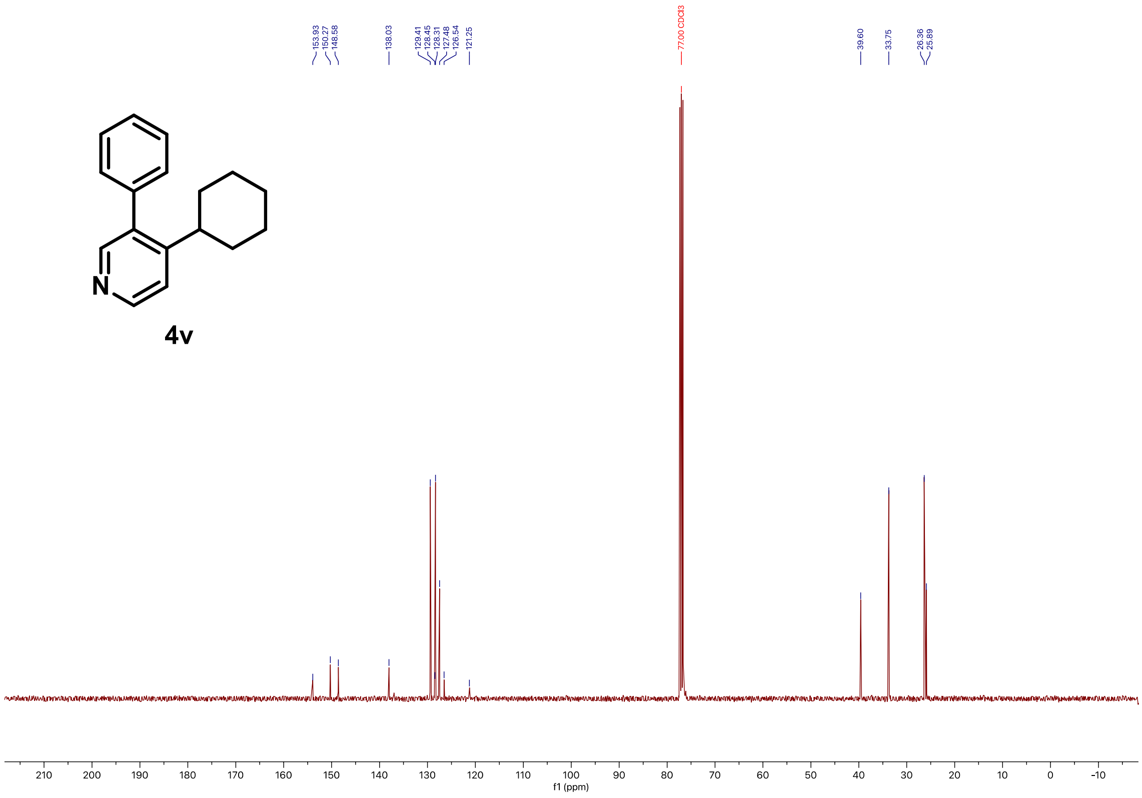

^{1}H NMR (400 MHz, $CDCl_3$) – **4w**

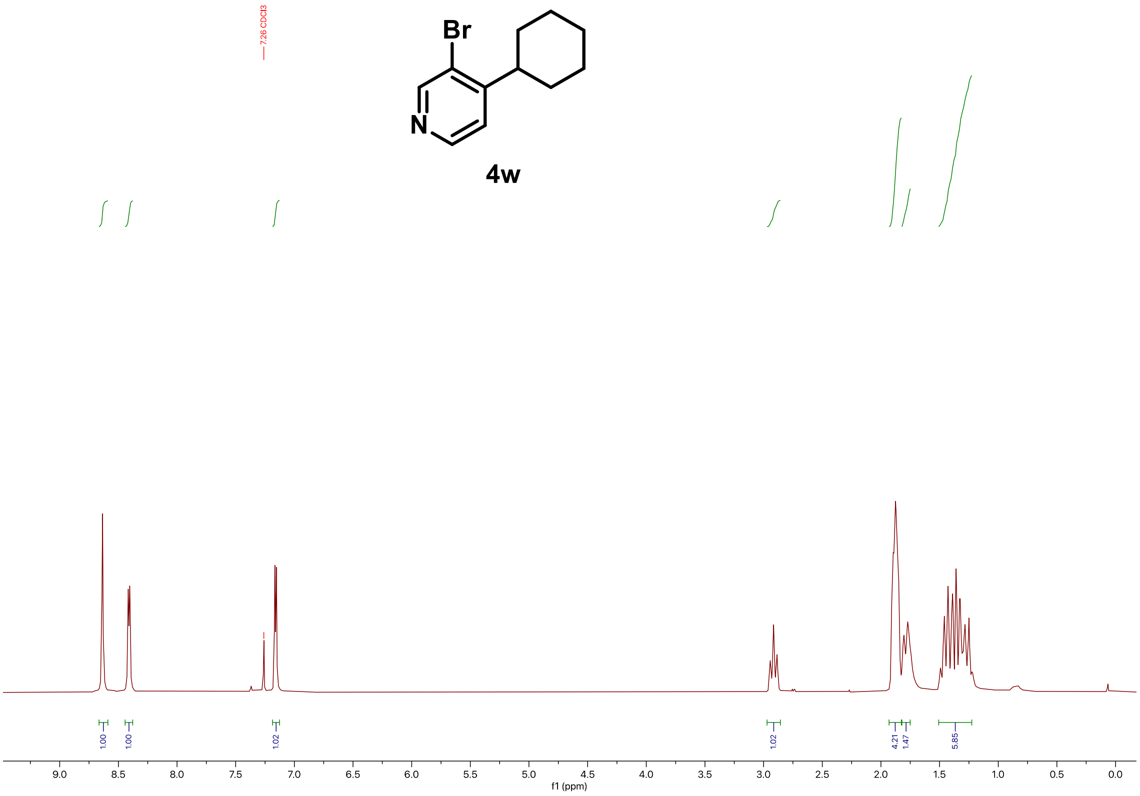

^{13}C NMR (101 MHz, $CDCl_3$) – **4w**

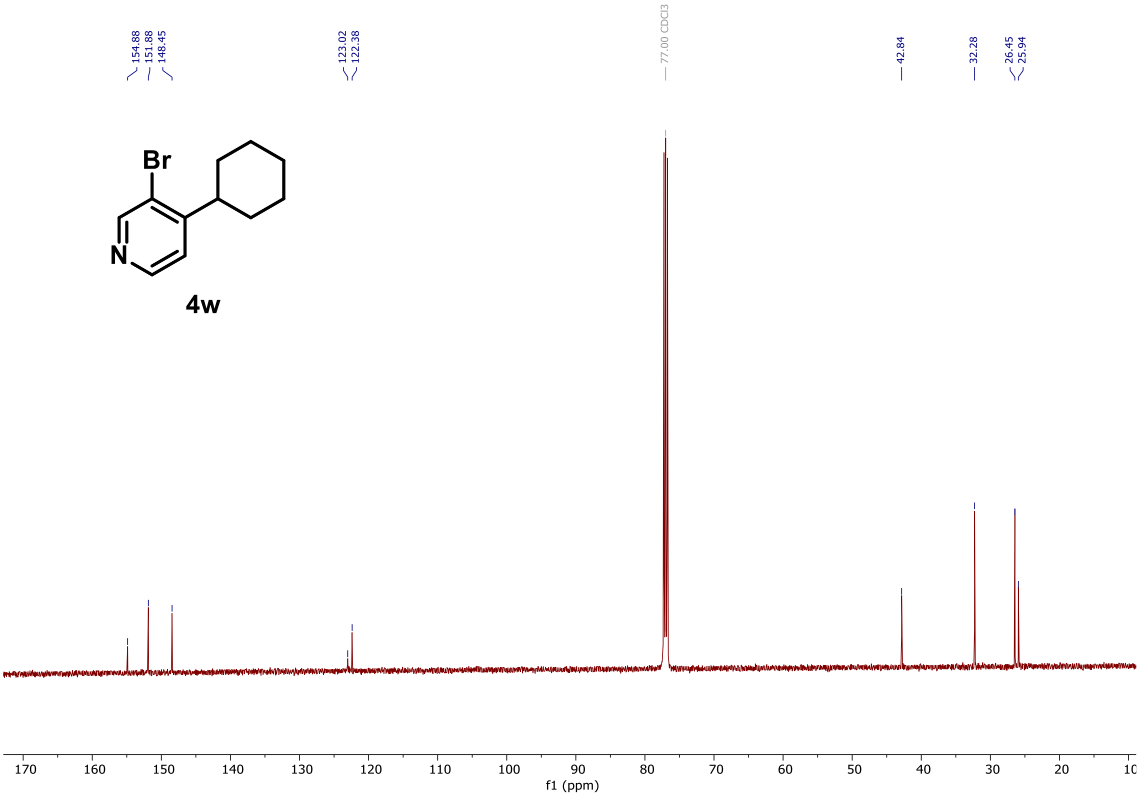

^{1}H NMR (400 MHz, $CDCl_3$) – **4x**

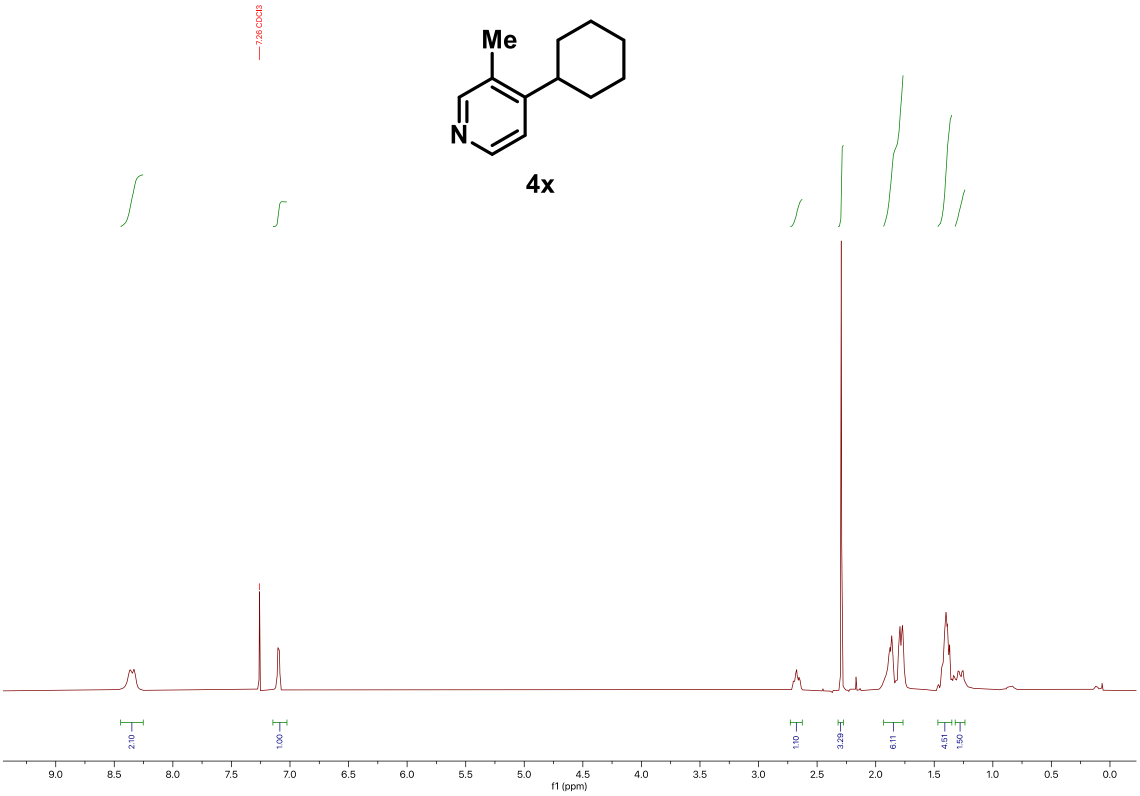

^{13}C NMR (101 MHz, $CDCl_3$) – **4x**

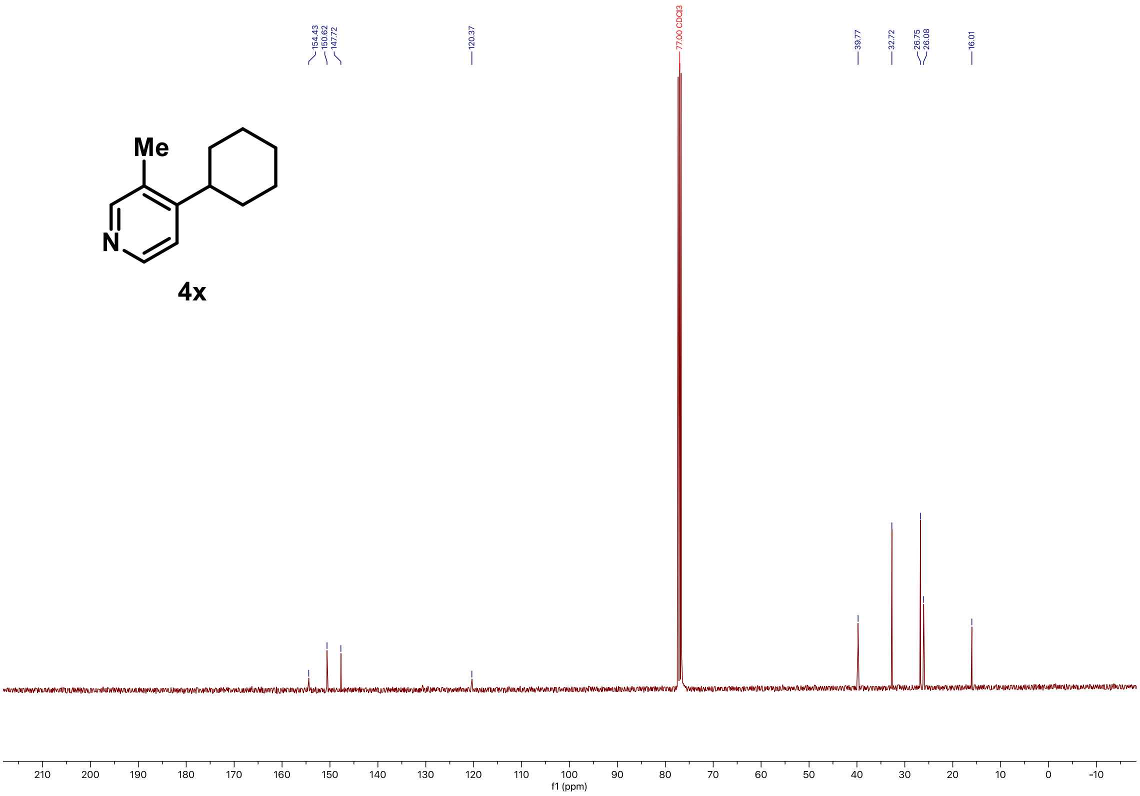

10 REFERENCES

1 J. Choi, G. Laudadio, E. Godineau and P. S. Baran, *J. Am. Chem. Soc.*, **2021**, *143*, 11927–11933.

2 Q. Chen, T. Leõn and P. Knochel, *Angew. Chem., Int. Ed.*, **2014**, *53*, 8746–8750.

3 G. A. Molander, O. A. Argintaru, I. Aron and S. D. Dreher, *Org. Lett.*, **2010**, *12*, 5783–5785.

4 T. Hoshikawa and M. Inoue, *Chem. Sci.*, **2013**, *4*, 3118.

5 J. A. Bristol and R. Brambilla, *J. Org. Chem.*, **1979**, *44*, 1889–1891.

6 L. Gao, G. Wang, J. Cao, H. Chen, Y. Gu, X. Liu, X. Cheng, J. Ma and S. Li, *ACS Catal.*, **2019**, *9*, 10142–10151.

7 S. Ohmiya, M. Tsuji, K. Higashiyama, T. Yamauchi and H. Kubo, *Heterocycles*, **2001**, *54*, 1027.

8 K. Matsumoto, M. Nakajima, T. Nemoto, *J. Org. Chem*. **2020**, *85*, 11802–11811.

9 L. Zhang, B. Pfund, O. S. Wenger, X. Hu, *Angew. Chem*. **2022**, *134*, e202202649; *Angew. Chem. Int. Ed*. **2022**, *61*, e202202649.

10 Gaussian 16, Revision B.01, M. J. Frisch, G. W. Trucks, H. B. Schlegel, G. E. Scuseria, M. A. Robb, J. R. Cheeseman, G. Scalmani, V. Barone, G. A. Petersson, H. Nakatsuji, X. Li, M. Caricato, A. V. Marenich, J. Bloino, B. G. Janesko, R. Gomperts, B. Mennucci, H. P. Hratchian, J. V. Ortiz, A. F. Izmaylov, J. L. Sonnenberg, D. Williams-Young, F. Ding, F. Lipparini, F. Egidi, J. Goings, B. Peng, A. Petrone, T. Henderson, D. Ranasinghe, V. G. Zakrzewski, J. Gao, N. Rega, G. Zheng, W. Liang, M. Hada, M. Ehara, K. Toyota, R. Fukuda, J. Hasegawa, M. Ishida, T. Nakajima, Y. Honda, O. Kitao, H. Nakai, T. Vreven, K. Throssell, J. A. Montgomery, Jr., J. E. Peralta, F. Ogliaro, M. J. Bearpark, J. J. Heyd, E. N. Brothers, K. N. Kudin, V. N. Staroverov, T. A. Keith, R. Kobayashi, J. Normand, K. Raghavachari, A. P. Rendell, J. C. Burant, S. S. Iyengar, J. Tomasi, M. Cossi, J. M. Millam, M. Klene, C. Adamo, R. Cammi, J. W. Ochterski, R. L. Martin, K. Morokuma, O. Farkas, J. B. Foresman, and D. J. Fox, Gaussian, Inc., Wallingford CT, 2016.

11 J.-D. Chai, M. Head-Gordon, *Phys. Chem. Chem. Phys*. **2008**, *10*, 6615–6620.

12 P. C. Hariharan, J. A. Pople, *Theor. Chim. Acta* **1973**, *28*, 213–222.

13 M. Cossi, N. Rega, G. Scalmani, V. Barone, *J. Comput. Chem*. **2003**, *24*, 669–681.

14 H. Fuse, Y. Irie, M. Fuki, Y. Kobori, K. Kato, A. Yamakata, M. Higashi, H. Mitsunuma, M. Kanai, *J. Am. Chem. Soc.* **2022**, *144*, 6566−6574.

15 M. Álvarez-Moreno, C. de Graaf, N. López, F. Maseras, J. M. Poblet, C. Bo, *J. Chem. Inf. Model*. **2015**, *55*, 95−103.
